# Combining Experimental and Computational Methods to Produce Conjugates of Anticholinesterase and Antioxidant Pharmacophores with Linker Chemistries Affecting Biological Activities Related to Treatment of Alzheimer’s Disease

**DOI:** 10.3390/molecules29020321

**Published:** 2024-01-09

**Authors:** Galina F. Makhaeva, Nadezhda V. Kovaleva, Elena V. Rudakova, Natalia P. Boltneva, Sofya V. Lushchekina, Tatiana Y. Astakhova, Elena N. Timokhina, Igor V. Serkov, Alexey N. Proshin, Yuliya V. Soldatova, Darya A. Poletaeva, Irina I. Faingold, Viktoriya A. Mumyatova, Alexey A. Terentiev, Eugene V. Radchenko, Vladimir A. Palyulin, Sergey O. Bachurin, Rudy J. Richardson

**Affiliations:** 1 Institute of Physiologically Active Compounds at Federal Research Center of Problems of Chemical Physics and Medicinal Chemistry, Russian Academy of Sciences, Chernogolovka 142432, Russia; gmakh@ipac.ac.ru (G.F.M.); kovalevanv@ipac.ac.ru (N.V.K.); rudakova@ipac.ac.ru (E.V.R.); boltneva@ipac.ac.ru (N.P.B.); sofya.lushchekina@gmail.com (S.V.L.); serkoviv@mail.ru (I.V.S.); proshin@ipac.ac.ru (A.N.P.); genie@qsar.chem.msu.ru (E.V.R.); vap@qsar.chem.msu.ru (V.A.P.); bachurin@ipac.ac.ru (S.O.B.); 2 Emanuel Institute of Biochemical Physics Russian Academy of Sciences, Moscow 119334, Russia; 3 Federal Research Center of Problems of Chemical Physics and Medicinal Chemistry, Russian Academy of Sciences, Chernogolovka 142432, Russia; soldatovayv@gmail.com (Y.V.S.); dapol@icp.ac.ru (D.A.P.); ifaingold@mail.ru (I.I.F.); mumyatova@icp.ac.ru (V.A.M.); alexei@icp.ac.ru (A.A.T.); 4 Department of Chemistry, Lomonosov Moscow State University, Moscow 119991, Russia; 5 Department of Environmental Health Sciences, University of Michigan, Ann Arbor, MI 48109, USA; 6 Department of Neurology, University of Michigan, Ann Arbor, MI 48109, USA; 7 Center of Computational Medicine and Bioinformatics, University of Michigan, Ann Arbor, MI 48109, USA; 8 Michigan Institute for Computational Discovery and Engineering, University of Michigan, Ann Arbor, MI 48109, USA

**Keywords:** Alzheimer’s disease (AD), 4-amino-2,3-polymethylenequinolines, butylated hydroxytoluene (BHT), acetylcholinesterase (AChE), butyrylcholinesterase (BChE), antioxidants, ADMET, β-amyloid, molecular docking, quantum-chemical calculations

## Abstract

Effective therapeutics for Alzheimer’s disease (AD) are in great demand worldwide. In our previous work, we responded to this need by synthesizing novel drug candidates consisting of 4-amino-2,3-polymethylenequinolines conjugated with butylated hydroxytoluene via fixed-length alkylimine or alkylamine linkers (spacers) and studying their bioactivities pertaining to AD treatment. Here, we report significant extensions of these studies, including the use of variable-length spacers and more detailed biological characterizations. Conjugates were potent inhibitors of acetylcholinesterase (AChE, the most active was **17d** IC_50_ 15.1 ± 0.2 nM) and butyrylcholinesterase (BChE, the most active was **18d**: IC_50_ 5.96 ± 0.58 nM), with weak inhibition of off-target carboxylesterase. Conjugates with alkylamine spacers were more effective cholinesterase inhibitors than alkylimine analogs. Optimal inhibition for AChE was exhibited by cyclohexaquinoline and for BChE by cycloheptaquinoline. Increasing spacer length elevated the potency against both cholinesterases. Structure–activity relationships agreed with docking results. Mixed-type reversible AChE inhibition, dual docking to catalytic and peripheral anionic sites, and propidium iodide displacement suggested the potential of hybrids to block AChE-induced *β*-amyloid (A*β*) aggregation. Hybrids also exhibited the inhibition of A*β* self-aggregation in the thioflavin test; those with a hexaquinoline ring and C8 spacer were the most active. Conjugates demonstrated high antioxidant activity in ABTS and FRAP assays as well as the inhibition of luminol chemiluminescence and lipid peroxidation in mouse brain homogenates. Quantum-chemical calculations explained antioxidant results. Computed ADMET profiles indicated favorable blood–brain barrier permeability, suggesting the CNS activity potential. Thus, the conjugates could be considered promising multifunctional agents for the potential treatment of AD.

## 1. Introduction

Considering the myriad possibilities of new products that could be produced by the judicious application of experimental and computational methodologies, we believe that one of the most pressing needs is to devise effective therapeutic agents for the prevention and/or treatment of Alzheimer’s disease (AD).

AD is a devastating disorder that kills neurons in areas of the brain involved in the retrieval and formation of memories, execution of other cognitive functions, and control of emotion and associated behaviors [1,2]. Apart from agents that provide mild cognition enhancement during the initial phases of the disease and palliative measures during the later severe stages, there are currently no effective preventions or treatments for AD. Moreover, given that the main risk factor for AD is advanced age, as the global elderly population increases, the negative impacts of the disease on afflicted individuals, their caregivers, and the economy are projected to intensify [3,4].

Intensive research has revealed pathological hallmarks of AD, but the cause and mechanism of the disease currently remain unknown. However, there is now general agreement that AD has multiple causes and that therapeutic agents will need to be designed to act on more than one target to be effective [5,6,7,8].

We have chosen to combine, in single molecules, the ability to modify three processes known to participate in AD pathogenesis, as summarized below:

1. Inhibition of acetylcholinesterase (AChE) and butyrylcholinesterase (BChE) help restore decreased levels of acetylcholine. Three cholinesterase inhibitors are already in clinical use (Donepezil (Aricept), Galantamine (Reminyl), and Rivastigmine (Exelon)) [9,10,11]. However, these drugs produce a range of unpleasant side effects from cholinergic hyperstimulation in the peripheral and/or central nervous systems, indicating a need for refinement of the pharmacokinetic and pharmacodynamic profiles of new cholinesterase inhibitors intended as AD therapeutics [12,13].
2. Inhibition of oxidants and free radicals to limit oxidative stress can both promote and be fueled by pathological states associated with neurodegeneration, such as the dysfunction of mitochondria, disruption of metal ion homeostasis, and formation and deposition of Aβ aggregates [14,15,16]. AD therapies employing antioxidants are currently under active investigation [17,18].
3. Inhibition of β-amyloid (Aβ) aggregation, which can form pathogenic Aβ plaques in the brain [19,20]. Compounds that inhibit Aβ aggregation are thought to have an ameliorative disease-modifying effect [21,22].

Along with its main function to hydrolyze acetylcholine, AChE has proaggregant properties toward β-amyloid via involvement of its peripheral anionic site (PAS), which interacts with soluble β-amyloid peptides to promote their aggregation [23,24,25,26]. Moreover, dual-binding molecules that interact with both the catalytic active site (CAS) and the PAS of AChE can inhibit AChE activity and block its amyloidogenic properties as well. Such compounds could simultaneously improve cognitive function and exert positive disease-modifying properties [27,28,29].

BChE has also been shown to participate in one or more steps leading to Aβ aggregation [30,31]. Consequently, from the standpoints of cognition enhancement by elevating ACh levels as well as disease modification by blocking Aβ aggregation, it makes sense to search for compounds capable of inhibiting both AChE and BChE.

One of the promising paradigms in anti-AD drug development is the design of multi-target ligands, whereby two molecules with different pharmacological propensities are linked together through a spacer of varying length (throughout this article, we use the terms “spacer” and “linker” interchangeably). For example, a component exhibiting anticholinesterase activity could be joined with another moiety exerting antioxidant properties [32]. Tacrine is widely employed as an anticholinesterase pharmacophore to create multifunctional cholinesterase inhibitors possessing additional neuroprotective and disease-modifying properties [33,34,35,36,37,38]. In particular, hybrids of tacrine coupled with antioxidants have been a popular combination [38,39,40,41,42]. 

Recently, we applied the multifunctional approach to create new hybrid structures using the 4-amino-2,3-polymethylenequinoline scaffold, which included tacrine and its cyclic homologs, and the sterically hindered phenolic scaffold of butylated hydroxytoluene (BHT) as the antioxidant pharmacophore [43]. The present research described in this article represents our continuing development and characterization of multifunctional hybrids.

**Scheme 1:**
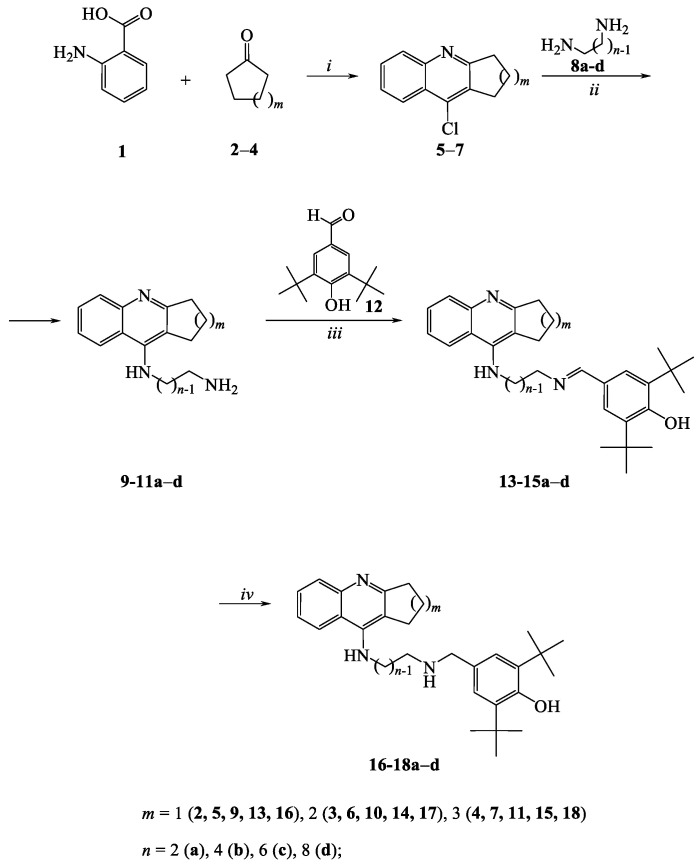
Synthesis of conjugates **13**–**15** and **16**–**18** showing variations in both ring size and spacer length. *i*: POCl_3_, reflux, 3 h; *ii*: diaminoalkanes **8a**–**d**, phenol, 180 °C, 3 h; *iii*: 3,5-di-tert-butyl- 4-hydroxybenzaldehyde **12**, toluene, 110 °C, 3 h; *iv*: NaBH_4_, MeOH, 25 °C, 3 h.

Here, we report significant extensions to our previously published work. The current investigation includes the expanded synthesis and more extensive biological activity evaluations of novel conjugates of 4-amino-2,3-polymethylenequinoline with various sizes of the aliphatic ring, which were connected to BHT by iminoalkyl or aminoalkyl spacers. Whereas our previous studies employed a fixed-length spacer, the present work involved spacers of increasing length (Scheme 1). Given that these compounds were produced as potential multifunctional agents for the treatment of AD, our biological evaluations were focused on endpoints relevant for this disease. First, we determined the esterase profile of the compounds, i.e., their inhibitory activities against AChE, BChE, and a structurally related off-target carboxylesterase (CES, EC 3.1.1.1). Second, we assessed the ability of the conjugates to inhibit Aβ self-aggregation and to displace propidium iodide from the PAS of AChE as a measure of their potential to block the AChE-induced aggregation of Aβ. Third, we determined their primary antioxidant activity and their antioxidant activity in rat brain homogenate. Fourth, the experimentally observed effects were analyzed using detailed quantum–mechanical (QM) calculations and computational molecular modeling. Finally, we carried out computational predictions of ADMET properties of the conjugates.

## 2. Results and Discussion

### 2.1. Chemistry

As shown in Scheme 1, at the first synthetic stage, tricyclic 4-chloro derivatives of quinoline with different cycloalkyl fragments **5**–**7** were synthesized. They were obtained by condensation of anthranilic acid **1** with cyclic ketones: cyclopentanone **2**, cyclohexanone **3** and cycloheptanone (suberone) **4**. The condensation was carried out by boiling the starting components in phosphorus oxychloride (POCl_3_) [44]. At the second stage of the synthesis, alkyl spacers with different carbon chain lengths were added to the obtained tricyclic derivatives of 4-chloropyridine **5**–**7** by reaction with diaminoalkanes **8a**–**d**. 

The addition of diamines **8a**–**d** was carried out by boiling the reaction mixture in pentanol [43], which afforded the aminoalkyl derivatives of 4-amino-2,3-polymethylene- quinoline **9**–**11a**–**d** with a free amino group necessary for the addition of the antioxidant phenolic fragment in the next step. At the second stage, conjugates of 4-amino-2,3-poly- methylenequinoline and an antioxidant were synthesized by adding a phenolic fragment to the free amino group of aminoalkyl derivatives of 4-amino-2,3-polymethylenequin- oline **9**–**11a**–**d**. First, by boiling in a mixture of toluene-methanol (5:1), 3,5-di-tert-butyl- 4-hydroxybenzaldehyde **12** was added as an antioxidant fragment with the formation of conjugates in which two pharmacophores were connected via an imine bond **13**–**15a**–**d**. The reduction in the obtained compounds with sodium borohydride in methanol led to the preparation of conjugates in which two pharmacophores were connected via an amine bond, **16**–**18a**–**d**.

### 2.2. Biological Studies

#### 2.2.1. Studies of AChE, BChE and CES Inhibition—Structure–Activity Relationships

We evaluated the esterase profile [45,46], of new potential anti-AD molecules that included the assessment of inhibition of cholinergic targets AChE and BChE, as well as the off-target CES, which hydrolyzes numerous ester-containing drugs [46]. The inhibitory activities of the conjugates against the esterases were characterized as IC_50_ values or as the inhibition percent at an inhibitor concentration of 20 µM for low-activity compounds. Tacrine, an effective AChE and BChE inhibitor, and bis-4-nitrophenyl phosphate (BNPP), a selective CES inhibitor, were used as positive controls. The results are shown in Table 1.

The study of the esterase profile of the synthesized conjugates **13**–**18** showed that the compounds effectively inhibit cholinesterases with predominant BChE inhibition (Table 1) and rather weakly inhibit the off-target CES.

**Effect of ring size**. As can be observed from Table 1, compounds with a cyclohexaquinoline ring (m = 2, i.e., a tacrine pharmacophore) showed the maximum activity against AChE; and compounds with a cycloheptaquinoline ring (m = 3) showed the maximum activity against BChE. This effect was observed for conjugates with different lengths (n) and types (imine or amine) of spacers.

**Effect of spacer length**. An increase in the length of the spacer led to a significant increase in anti-AChE activity, both for compounds with alkylimine (**13**–**15**) spacers (45- to 350-fold) and alkylamine (**16**–**18**) spacers (40- to 60-fold). Conjugates with spacer length *n* = 8 (**14d**, **15d**; **17d**, **18d**) showed the maximum inhibitory activity against AChE, among which tacrine derivatives (m = 2) had the highest activity—40 times higher than the activity of the basic pharmacophore tacrine.

An increase in the length of the spacer also led to an increase in anti-BChE activity. The most active BChE inhibitors with IC_50_ values in the nanomolar region were conjugates with spacer length *n* = 8 (**14d**, **15d**; **17d**, **18d**). Considering the influence of the ring size, the cycloheptaquinoline (m = 3) conjugates **15d** and **18d** exhibited the maximum activity in this series with an IC_50_ of 6 nM. 

**Effect of spacer chemistry.** The structure of the spacer also affected activity: in general, conjugates with an alkylamine spacer (**16**–**18**) were more effective at inhibiting AChE and BChE than their analogs with an alkylimine spacer (**13**–**15**), and the effect of replacing the imine spacer with an amine spacer was more pronounced for BChE inhibition.

#### 2.2.2. Kinetic Studies of AChE and BChE Inhibition

**Figure 1:**
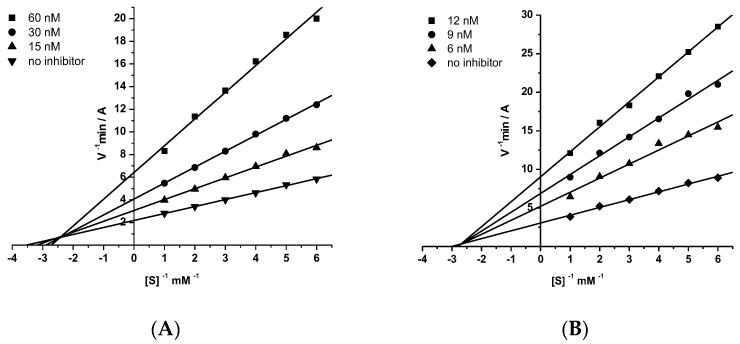
Steady state inhibition of AChE (**A**) and BChE (**B**) by compound **15d**.

The mechanism of inhibition of AChE and BChE by the conjugates was studied using compounds **15a**,**c**,**d** and **18c**,**d**. The graphical analysis of the kinetic data on AChE and BChE inhibition by compound **15d** (Figure 1A,B) with Lineweaver–Burk plots demonstrated changes in both *K*_m_ and *V*_max_ values—a result consistent with a mixed type of inhibition. The values obtained for the competitive (*K*i) and noncompetitive (α*K*i) components of the constants for AChE inhibition by compound **15d** were 19.9 ± 0.8 nM and 31.4 ± 2.4 nM, respectively; and for BChE 4.91 ± 0.42 nM and 5.68 ± 0.06 nM, respectively.

The obtained inhibition constants of cholinesterases by the studied conjugates are presented in Table 2.

#### 2.2.3. Molecular Docking to AChE and BChE

**Figure 2:**
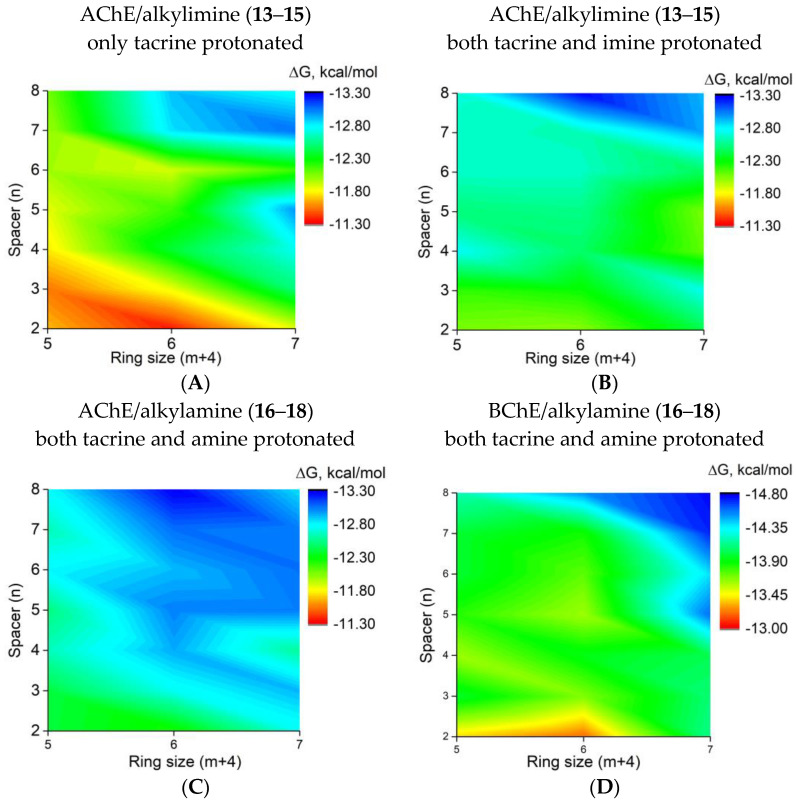
Estimated binding affinities from molecular docking to AChE of alkylimine (**13**–**15**) compounds with protonated tacrine group (**A**), and both tacrine and imine groups protonated (**B**) in comparison with results for alkylamine (**16**–**18**) derivatives, with both tacrine and the secondary amine groups protonated and docked to AChE (**C**) and BChE (**D**). Plots A–C have the same energy scale, which differ from the energy scale for BChE in panel D.

Results of molecular docking were in good agreement with the experimentally observed effects of the ring size and linker length (Figure 2). According to the p*K*_a_ estimations, the 4-amino-2,3-polymethylenequinoline fragment and secondary amine group of the linker would be protonated at the experimental conditions (pH 7.5) [38,47]. 

In contrast, for the imine group, calculated p*K*a values were close to 7. This indicates the possibility of co-existence of protonated and non-charged imino-groups at the experimental pH value; therefore, both possibilities were considered. The binding of alkylimine compounds with the protonated imine group was stronger than for the non-protonated compounds (Figure 2A,B). Due to their higher flexibility, the binding of compounds with the alkylamino group was stronger than that of the protonated alkylimine derivatives (Figure 2C).

**Figure 3:**
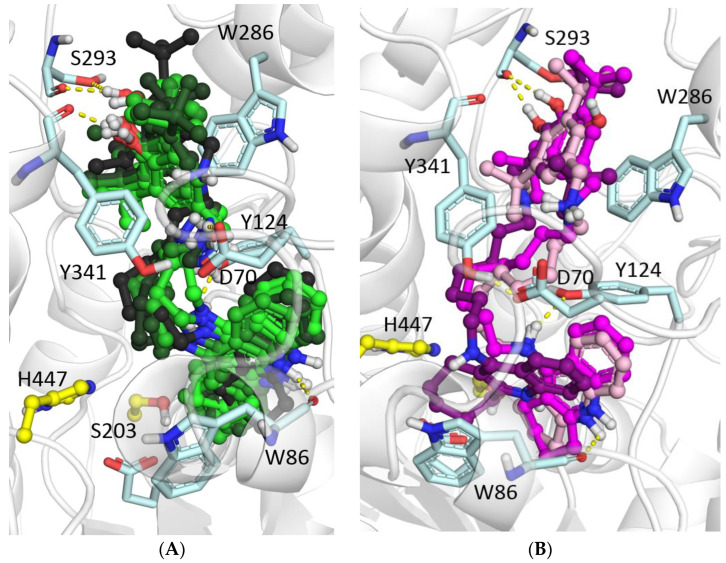
Binding poses inside AChE for alkylamine derivatives with (**A**) increasing linker length (**17a**–**17d**; carbon atoms are colored with different shades of green, from the lightest for *n* = 2 to the darkest for *n* = 8) and (**B**) increasing ring size (**16d**, carbon atoms are shown as violet, **17d**—purple, and **18d**—deep purple).

The increase in the length of the linker in all considered cases led to a better binding affinity due to the increased occupancy of the PAS, which is typical of tacrine-based AChE inhibitors [37,38]. While the tacrine fragment interacted with the active site at the bottom of the gorge, the BHT fragment interacted with the PAS. For compounds with shorter linkers, their hydroxyl groups formed a hydrogen bond with the Tyr341 main chain oxygen atom. In contrast, for compounds with the longest linker (*n* = 8), hydrogen bonding with polar atoms of Ser293 was possible (Figure 3A). The positively charged amine groups of the shorter linkers interacted with Asp70 and Tyr341 side chains. For the longest linkers, there were π-cation interactions with the Trp286 side chain. At the same time, the cyclohexaquinoline ring (m = 2) was optimal for AChE active site binding, while a further increase in the ring size led to displacement of the tacrine fragment (Figure 3B, [37,43]). This optimal structural binding was mirrored by minimal binding free energies (binding affinities) obtained from the molecular docking simulations. In the case of binding to BChE, which has a wider gorge than that of AChE, the best binding was achieved for the compound with the maximum spacer length (*n* = 8) and ring size (C-7, m = 3) (Figure 2D).

#### 2.2.4. Displacement of Propidium from the PAS of *Ee*AChE

The results of the study of propidium displacement from the AChE PAS by conjugates **13**–**18** are presented in Table 1.

As can be observed from Table 1, all studied conjugates with alkylimine (**13**–**15**) and alkylamine (**16**–**18**) spacers at a concentration of 20 μM reduced the fluorescence intensity by 12–20%. Accordingly, they displaced propidium from PAS of AChE almost at the level of the reference compound donepezil or exceeded it in efficiency. In addition, in the group of conjugates with an alkylimine spacer, the decrease in fluorescence intensity was somewhat stronger than for compounds with an alkylamine spacer and amounted to 14–20% and 12–17%, respectively.

These displacement results, along with the mixed type of AChE inhibition demonstrated by enzyme kinetics and the results of molecular docking, which showed a binding of the conjugates to both the CAS and PAS of AChE, all support the ability of these compounds to block the AChE-induced aggregation of Aβ_42_.

#### 2.2.5. Inhibition of β-Amyloid (1–42) (Aβ_42_) Self-Aggregation

The most active cholinesterase inhibitors with spacers of 6 and 8 methylene groups were studied as inhibitors of Aβ_42_ self-aggregation (Table 3).

The results presented in Table 3 demonstrate that the studied conjugates exhibited inhibitory activity against Aβ_42_ self-aggregation in the range from 43.4% to 71.4%. Moreover, the degree of inhibition of Aβ_42_ self-aggregation depended on both the size of the aliphatic ring in the tacrine fragment (m = 1–3) and the length of the spacer (*n* = 6 or 8).

The most active compounds were the conjugates of BHT and 4-amino-2,3-poly- methylenequinoline containing a hexaquinoline ring (m = 2), combined with the alkylimine **14d** and alkylamine spacer **17d** (*n* = 8), which exhibited inhibitory activity against the Aβ_42_ self-aggregation comparable to the level of the reference compound Myricetin (73.2 ± 5.8%). Note that tacrine on its own exhibited minimal inhibition, and the inhibition by BHT alone was not detectable 

#### 2.2.6. Molecular Docking to Aβ_42_

**Figure 4:**
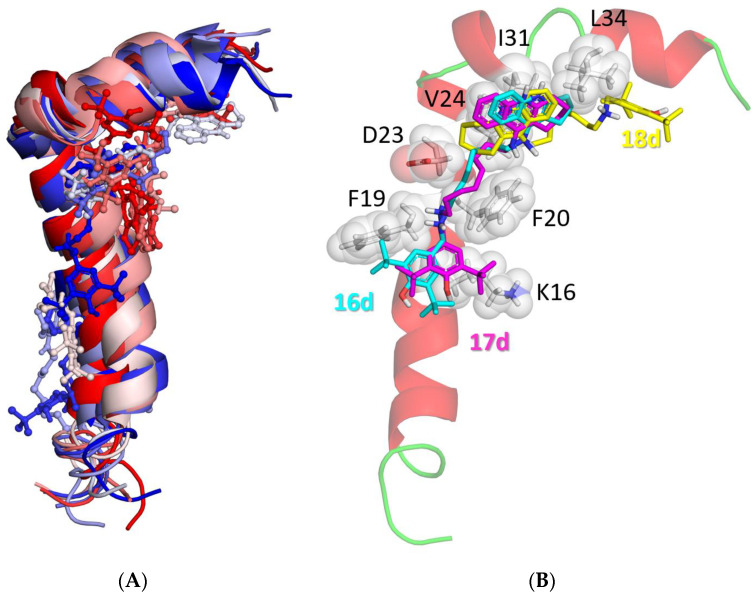
Molecular docking to Aβ_42._ (**A**) All conformers of PDB ID 1IYT and compound **17d** (the color gradually changes from red through white to blue from the first to the tenth Aβ_42_ conformer); (**B**) Aβ_42_ conformer #10 with the best energy of binding of compound **17d** (carbon atoms colored in magenta) in comparison with positions of compounds **16d** (carbon atoms colored in cyan), and **18d** (carbon atoms colored in yellow).

Our previous studies demonstrated that the results of docking ligands to Aβ_42_ depend significantly on the conformation of the peptide [38,48]. Accordingly, as the docking target, we have used all 10 conformers of Aβ_42_ that are available in the NMR solution structure, PDB ID 1IYT. Among the different binding poses (Figure 4A), the majority was in the hydrophobic area of the turn segment and the C-terminal part. This region is crucial for the conformational transition at the initial phase of Aβ_42_ nucleation during fibril formation [49]. A long linker in the ligand ensures the binding of one of the pharmacophores (BHT) to hydrophobic residues Lys16, Phe19, and Phe20 on one side (Figure 4B, compounds **16d, 17d**), while the other pharmacophore (tacrine) binds with Val24, Ile31, and Leu34 at the turn segment and the C-terminal part of the peptide. The larger tacrine ring (m = 2 for **18d**) did not fit well between residues Ile31 and Leu34, which changed the position of the compound relative to the peptide.

#### 2.2.7. Antioxidant Activity

Primary antioxidant activity of the conjugates was determined using two spectrophotometric tests: the ABTS radical-scavenging assay and the Fe^3+^-reducing antioxidant power (FRAP) assay.

##### ABTS Assay

All synthesized conjugates exhibited high ABTS^•+^-scavenging activity, close to or exceeding the activity of the standard antioxidant Trolox and the basic antioxidant pharmacophore BHT (TEAC = 0.78–1.5) (Table 4). The structure of the spacer influenced the ABTS^•+^-binding activity. Thus, the replacement of an alkylimine spacer (conjugates **13**–**15**) with an alkylamine one (conjugates **16**–**18**) generally led to an increase in the radical-scavenging activity, with a maximum difference of 1.5-fold. Most alkylamino analogs (**16**–**18**) demonstrated a radical-scavenging activity that was noticeably higher than that of Trolox (TEAC = 1.2–1.5).

For an adequate assessment of the antiradical activity of tested compounds and for revealing structure–activity relationships, it is also important to consider the initial reaction rate of ABTS radical scavenging [50,51]. Our experiments showed that the lead conjugates with alkylamine spacers (**16c**, **17b**, **17c**, **17d**, **18b**, **18d**) had a rather high initial rate of ABTS radical scavenging. For these compounds, the time to reach the activity level of Trolox when used in a concentration equal to its IC_50_ (20 μM) was less than 1 min, whereas for the other alkylamine derivatives, this interval increased to 3 to 5 min.

Alkylimine analogs (**13**, **14**, **15**) exhibited antiradical activity at the level of Trolox (TEAC = 0.78–1.13). However, they demonstrated a lower initial rate of binding of the ABTS radical: the time to reach the degree of radical binding at the Trolox level ranged from 5 to 60 min.

An increase in the size of the aliphatic ring of the 4-amino-2,3-polymethylene- quinoline (“tacrine”) fragment, as well as a change in the length of the spacer, did not substantially affect the antiradical activity of conjugates with either alkylimine or alkylamine spacers.

Thus, the results showed that the synthesized conjugates of cyclic homologs of tacrine and BHT exhibited high radical-scavenging activity in the ABTS test. Conjugates with an alkylamine spacer were more active compared to their alkylimine analogs. Maximum activity, exceeding the activity of the standard antioxidant Trolox, was demonstrated by conjugates **16c**, **17b**, **17c**, **17d**, **18b**, **18d**.

##### FRAP Assay

As can be observed from Table 4, conjugates **13**–**15** and **16**–**18** had a high iron-reducing ability, which, however, was somewhat lower than that of Trolox and the basic pharmacophore BHT. In general, conjugates with an alkylimine spacer were somewhat more active than their alkylamine analogs. 

The size of the aliphatic “tacrine” ring had practically no effect on the ability of the conjugates to reduce Fe^3+^. The activity somewhat increased with the elongation of the spacer, which was more evident in the case of conjugates with an alkylimine spacer.

#### 2.2.8. Antioxidant Activity of Conjugates in a Biological System

To obtain information about the antioxidant activity of conjugates in biological systems, we estimated their free radical scavenging activity in a mouse brain homogenate by a chemiluminescence (CL) method and studied their ability to suppress spontaneous lipid peroxidation (LP) by the TBARS assay. Three pairs of conjugates with an imine and amine fragment in the spacer and tacrine moiety ring 6 (m = 2) and 7 (m = 3) were selected for study. For conjugates with a 7-membered ring in the tacrine moiety, compounds with spacer lengths (CH_2_)_6_ and (CH_2_)_8_ were studied. The results are presented in Table 5.

##### Radical Scavenging Activity in Mouse Brain Homogenate—Luminol CL Assay

In this method, luminol is used as a CL enhancer. The CL assay of the radical-scavenging capacity of the compounds was based on assessing the reduction in the luminol CL mediated by its interaction with free radicals, whose formation in the mouse brain homogenate was initiated by tert-butyl hydroperoxide (TBHP) [52]. Luminol allows detecting hydrogen peroxide (H_2_O_2_), hydroxyl radicals (OH), hypochlorite (ClO–), peroxynitrite (ONOO^–^), and lipid peroxyl radicals. 

The radical scavenging capacity of the tested compounds was characterized by IC_50_ values. As can be observed from Table 5, the conjugates demonstrated a high radical-scavenging capacity, which was markedly higher than that of the basic pharmacophore BHT. In addition, the radical-scavenging capacity was significantly higher for compounds with an alkylimine rather than an alkylamine spacer.

##### Inhibition of Spontaneous LP in Mouse Brain Homogenate—TBARS Assay

The reaction of malondialdehyde (MDA) with thiobarbituric acid (TBA) has been widely used as a sensitive method for LP assay in animal tissues [53]. We studied the impact of three pairs of conjugates on the formation of TBARS by following the reaction of oxidized lipids with TBA under the conditions of spontaneous LP in the mouse brain homogenate. The inhibition of LP was characterized by IC_50_ values (Table 5).

As can be observed from Table 5, conjugates effectively inhibited the process of spontaneous LP, and compounds with an amine-containing spacer were more effective than those with an imine-containing spacer. However, their activity was lower than that of the basic BHT pharmacophore.

#### 2.2.9. Quantum–Chemical Calculations 

Quantum–chemical methods were used to assess the primary antioxidant activity (AOA) of conjugates with imine- and amine-containing spacers. For this purpose, two compounds, **14b** (imine) and **17b** (amine), were chosen as examples. 

An analysis of all experimental conditions and quantum–chemical calculations, taking into account the p*K_a_* predictions described in Section 3.2.3, shows that in all tests for assessing AOA (ABTS test with pH ≈ 4.5, FRAP test with pH = 3.5 and CL and LP with pH = 7.4), both imines and amines would have been doubly protonated in the tacrine moiety and spacer (for details, see Supplementary Materials). Below, the state of protonation in the tacrine moiety and spacer is denoted by the subscripts t and s, respectively.

The compounds under consideration demonstrate different results in different antioxidant tests. In the ABTS test, **14b** has a lower AOA than **17b** (1.0 for **14b** vs. 1.4 for **17b**; see Table 4). Whereas in the FRAP test, the AOA of **14b** is higher than that of **17b** (0.72 for **14b** vs. 0.46 for **17b**; see Table 4). In the CL and LP tests, **14b** and **17b** were not investigated. However, the results for other compounds from corresponding series indicate that the CL radical-scavenging capacity of imines was much higher (3–6 times) than that of amines, and both imines and amines demonstrate markedly higher CL radical-scavenging capacity than the basic pharmacophore BHT (see Table 5). In the LP test, amines are more effective than imines. However, their LP activity is lower than that of the basic BHT pharmacophore (see Table 5).

Such different results of antioxidant tests can be explained by different mechanisms of AOA. To further characterize the AOA of the studied compounds on a theoretical basis by using quantum–chemical calculations, the bond dissociation enthalpy (BDE), vertical ionization potential (IP), vertical proton affinity (EA_v_) and proton dissociation enthalpy (PDE) were computed, as is commonly conducted [54,55,56] and displayed in Table 6. The solvation enthalpies of the proton and electron in water and ethanol were taken from the literature [57].

The lowest BDE value is given for each compound, which is for the OH bond in the BHT moiety. PDE values were also calculated for the OH bond in the BHT moiety (PDE^1^) and for NH bond in the spacer (PDE^2^). The mechanisms of AOA of the studied compounds in the CL, LP, ABTS and FRAP tests, as well as the main energetic characteristics describing these mechanisms, are discussed below.

##### Luminol Chemiluminescence

**Figure 5:**
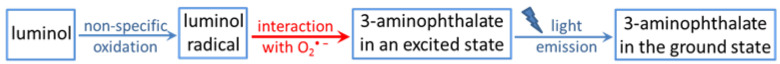
Main stages that control light emission in the CL test.

Superoxide anion O_2_^•−^ plays a key role in the CL assay [58]. It does not react directly with luminol. Instead, it reacts with the luminol radical anion (L^•−^) formed in the reactions of luminol with various radicals (e.g., HO^•^ and CO_3_^•−^). The reaction between the luminol radical and superoxide results in the formation of 3-aminophthalate in an excited state (see Figure 5). The luminescence of the latter is detected in the experiment [59]. Thus, in the CL test, the luminol luminescence can be reduced by quenching superoxide, which would be present in mouse brain homogenates [60]. The fundamentally different results obtained in CL and LP tests (see Table 5) suggest that it is the superoxide radical quenching that is responsible for the decrease in luminol luminescence in the CL assay.

An analysis of free energy changes in possible reactions for the superoxide quenching [61,62,63] (for details, see Supplemental Materials) shows that the superoxide quenching proceeds through proton donation by the antioxidant, which is similar to the dismutation of the superoxide radical (O_2_^•−^) into hydrogen peroxide (H_2_O_2_) and oxygen (O_2_) in biological systems, as depicted in Reaction (1) [64]:

2O_2_^•−^ + 2AH → H_2_O_2_ + O_2_ + 2A^−^ (1)

where AH is the antioxidant molecule and A^−^ is the deprotonated antioxidant. The energy benefit in Reaction (1) is determined by the PDE. As the PDE decreases, the ease of proton donation by the antioxidant molecule increases. BHT and **14b_ts_** can donate a proton from the OH bond in the BHT moiety with PDE values of 41.0 and 26.7 kcal/mol, respectively; compound **17b_ts_** donates a proton from the NH bond in the spacer with a PDE value of 32.9 kcal/mol. Thus, the AOA in the CL test is determined by the PDE; a difference in PDE values of 6 kcal/mol decreases the IC_50_ value in the CL test about six-fold.

##### Inhibition of Spontaneous Lipid Peroxidation

In spontaneous lipid peroxidation (LP), many different radicals are formed in brain homogenate. We estimated the ability to inhibit LP by the interaction of the considered antioxidants with the most active hydroxyl radical HO^•^. The direct pathway for hydroxyl radical quenching is the H-atom transfer from the antioxidant to the radical [65,66,67]:

AOH + HO^•^ → AO^•^ + H_2_O (2)

The energy benefit in reaction (2) is determined by the BDE. The lower the BDE, the easier it is for the antioxidant molecule to donate an H-atom. The BDE value of **17b_ts_** is lower than that of **14b_ts_**, resulting in a higher AOA of **17b_ts_** than that of **14b_ts_**. The BDE value of BHT is lower than that of either **14b_ts_** or **17b_ts_**, which correlates with the higher activity of BHT in the LP test.

In LP, a mixture of many different radicals is formed, the quenching mechanism of which may differ from the hydroxyl radical. Despite this, the BDE can be considered a rough but adequate characterization of AOA [68]. 

##### ABTS and FRAP Tests

In both the ABTS and FRAP tests, specially pre-generated cation-radicals/cations (ABTS^•+^ in the ABTS test and Fe^3+^[TPTZ]_2_ in the FRAP test) are reduced to their parent form by acquiring an electron because of the interaction with an antioxidant molecule. Regardless of the antioxidant mechanism, the pre-generated cation acquires an electron because of the antioxidant reaction. ABTS^•+^ and Fe^3+^[TPTZ]_2_ cations serve as markers. The decrease in their concentration characterizes the antioxidant capacity of the tested compounds. The decrease in the concentration of ABTS^•+^ and the increase in the concentration of Fe^2+^[TPTZ]_2_ are measured by the intensity of the characteristic bands in the UV-vis spectrum (734 and 593 nm in the ABTS and FRAP tests, respectively) [69]. 

The electron transfer (ET) from one molecule (donor) to another molecule (acceptor) proceeds via the formation of a reaction complex [70]. In antioxidant reactions, the antioxidant and cation molecules in the reaction complex are linked either by hydrogen bonding [71] or by Coulomb interaction. This is the case with the outer-sphere ET mechanism.

**Figure 6:**
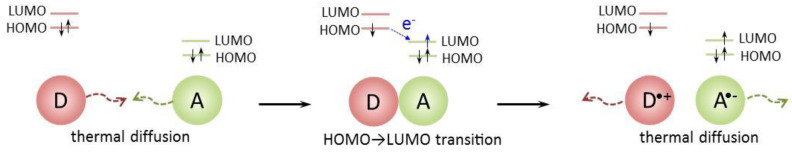
Three stages of the outer-sphere ET mechanism.

The ET process is illustrated in Figure 6 for a neutral donor (D) and acceptor (A). The process occurs in three stages. At the first stage, the precursor complex [D, A] is formed during thermal diffusion. 

At the second rate-limiting stage, the intra-complex ET results in the successor complex [D^+^, A^−^] formation. The electron passes from the donor HOMO to the acceptor LUMO. The ET occurs only if the donor HOMO lies energetically above the acceptor LUMO. The efficiency of this stage depends on the energy characteristics of the donor and acceptor. The greater the difference between the energies of the donor HOMO and the acceptor LUMO, the faster the ET.

The third stage of the ET is a diffusive separation of the [D^+^, A^−^] successor complex. 

Thus, ET efficiency is generally determined by two factors. The first is the energy benefit of the ET from a donor to an acceptor. For the same acceptor, ET is more efficient for a donor with a higher HOMO energy, i.e., a lower IP. 

The second factor is the efficiency of the precursor complex formation during thermal diffusion. The probability of precursor complex formation depends on the geometric structure (mutual orientation of molecules suitable for ET) and diffusion characteristics (probability of contact) of the donor and acceptor. Obviously, imines and amines have different diffusion characteristics due to the different spacer rigidity resulting in different probabilities of the precursor complex formation. (The same applies to the marker cations ABTS^•+^ and Fe^3+^[TPTZ]_2_.) The donor and acceptor in the complex should be oriented so that the electron can move from the donor HOMO to the acceptor LUMO. For the sterically hindered propeller-shaped Fe^3+^[TPTZ]_2_, complex formation is more difficult than for the quasi-one-dimensional ABTS^•+^. 

In addition, the presence of other species, such as cations and anions (buffer acetate anion CH_3_COO^−^ in the FRAP test, ammonium cation NH_4_^+^ and sulfate anion SO_4_^2−^ in the ABTS test, and some others), should influence the diffusion due to their interaction with both charged (protonated) antioxidants and the marker cations ABTS^•+^ and Fe^2+^[TPTZ]_2_. 

Thus, the same order of AOA of imines and amines in the ABTS and FRAP tests are explained by their nearly equivalent IP values (113.9 and 114.0 kcal/mol for **14_bt_** and **17_bt_**, respectively, see Table 6) characterizing the ability to donate an electron. Some difference in AOA is associated with the different thermal diffusion behavior due to the different spacer rigidity of both the antioxidant molecules and the marker cations ABTS^•+^ and Fe^3+^[TPTZ], as well as their interaction with other cations and anions in a reaction solution, which leads to different efficiencies of precursor complex formation.

### 2.3. Predicted ADMET Profiles and PAINS Analysis

The results of the computational evaluation of several important physicochemical and ADMET properties for compounds **13**–**18** are shown in Table 7. According to the predictions, all compounds should have high intestinal absorption and could be administered orally. Moreover, reasonable CNS bioavailability and activity could be expected based on the rather high predicted blood–brain barrier permeability (brain concentration is expected to be about 1.7- to 8-fold greater than the plasma concentration). Both parameters of the hERG-mediated cardiac toxicity risk (p*K_i_* and pIC_50_) for all the analyzed compounds varied from 5.7 to 7.4 log units, corresponding to the middle part of their possible range (3–9 log units). 

Following the commonly accepted drug-likeness recommendations, the molecular weights of the compounds, their predicted lipophilicities and aqueous solubilities were within or close to the ranges that are desirable for potential drug compounds, although the LogP values exceeded the original Rule-of-5 limits and the solubilities were in the micromolar or nanomolar range. However, these predicted values cannot be considered fully reliable, since some of the compounds were outside of the model applicability domain. The integral QED (quantitative estimate of drug-likeness) parameters are in the 0.2–0.4 range (based on the data for oral drugs, QED > 0.2 is desirable). No alerts were detected by the Pan Assay INterference compoundS (PAINS) filter check.

By and large, the predicted physicochemical, ADMET, and PAINS properties of the compounds seem quite acceptable for potential lead compounds in the drug discovery phase, although additional studies and optimization efforts could help to enhance their pharmacokinetic profile and maximize safety. 

## 3. Materials and Methods

### 3.1. Chemistry 

Melting points were recorded on the Stuart SMP10 Melting Point Apparatus (Stuart, Staffordshire, UK). 1 H-NMR (200 MHz) spectra were recorded on a DPX-200 NMR spectrometer (Bruker, Karlsruhe, Germany); chemical shifts, δ, are given in parts per million (ppm). CHN analysis was performed on the ER-20 analyzer (Carlo-Erba, Val-de-Reuil, France). All solvents, chemicals and reagents were obtained commercially.

#### 3.1.1. General Procedure for the Preparation of Derivatives **13**–**15a**–**d** and **16**–**18a**–**d**

A mixture of aminoquinoline **9**–**11a**–**d** (1.0 mmol) and 3,5-di-*tert*-butyl-4-hydroxy-benzaldehyde **12** (234 mg, 1.0 mmol) in toluene (10 mL) was stirred and refluxed for 3 h. Then, the solution was evaporated under a vacuum and the residue was washed with ether, yielding the target conjugates **13**–**15a**–**d**.

To a solution of compounds **13**–**15a**–**d** (1.0 mmol) in 5 mL of methanol, 57 mg (1.0 mmol) of sodium borohydride was added and the mixture was stirred for 1 h at room temperature. Methanol was evaporated, and 15 mL of methylene chloride was added and washed with water (2 × 10 mL). The organic layer was dried over anhydrous sodium sulfate. The drying agent was filtered, the filtrate was evaporated, and the residue was recrystallized in a benzene-methanol (5:1) mixture to give target conjugates **16**–**18a**–**d**.

#### 3.1.2. Synthesis of Compounds

The compounds **13a**, **14a**, **15a**, **16a** and **18a** were synthesized and described earlier in [43].

2,6-Di-*tert*-butyl-4-{[4-(2,3-dihydro-1*H*-cyclopenta[b]quinolin-9-ylamino)-butylimino]-methyl}-phenol (**13b**). Yellow solid; Yield 67%, m.p. 91–93 °C. ^1^H-NMR (CDCl_3_), δ: 1.45 (s, 18H, 6 × CH_3_), 1.66–1.86 (m, 4H, 2 × CH_2_), 2.02–2.23 (m, 2H, CH_2_), 3.08 (t, 2H, *J* = 7.4 Hz, CH_2_), 3.19 (t, 2H, *J* = 7.4 Hz, CH_2_), 3.50–3.82 (m, 4H, 2 × CH_2_), 5.11 (br.s, 1H, OH), 7.12–7.42 (m, 2H, 2 × H_ar_), 7.26 (s, 2 × 1H, 2 × H_ar_), 7.77 (d, 1H, *J* = 8.6 Hz, H_ar_), 7.94 (d, 1H, *J* = 8.6 Hz, H_ar_), 8.19 (s, 1H, =CH). Anal. Calcd. for C_31_H_41_N_3_O: C, 78.94; H, 8.76; N, 8.91. Found: C, 78.82; H, 8.84; N, 8.82.

2,6-Di-*tert*-butyl-4-{[6-(2,3-dihydro-1*H*-cyclopenta[b]quinolin-9-ylamino)-hexylimino]-methyl}-phenol (**13c**). Light brown solid; Yield 72%, m.p. 92–94 °C. ^1^H-NMR (CDCl_3_), δ: 1.15–1.30 (m, 2H, CH_2_), 1.44 (s, 18H, 6 × CH_3_), 1.56–1.82 (m, 6H, 3 × CH_2_), 2.12 (pent, 2H, *J* = 7.2 Hz, CH_2_), 3.07 (t, 2H, *J* = 7.6 Hz, CH_2_), 3.19 (t, 2H, *J* = 7.1 Hz, CH_2_), 3.41–3.72 (m, 4H, 2 × CH_2_), 5.08 (br.s, 1H, OH), 7.28–7.43 (m, 1H, H_ar_), 7.43–7.65 (m, 3H, 3 × H_ar_), 7.79 (d, 1H, *J* = 8.1 Hz, H_ar_), 7.92 (d, 1H, *J* = 8.1 Hz, H_ar_), 8.07 (s, 1H, =CH). ^13^C-NMR (CDCl_3_), δ: 22.43, 23.20, 26.13, 26.56, 26.97, 30.12 (6), 30.93, 31.14, 34.36, 34.77, 45.61, 113.93, 118.75, 119.75, 123.96, 125.34, 125.42, 125.77, 127.61, 128.30, 128.73, 136.72, 146.65, 147.78, 168.19. Anal. Calcd. for C_33_H_45_N_3_O: C, 79.31; H, 9.08; N, 8.41. Found: C, 79.43; H, 9.00; N, 8.50.

2,6-Di-*tert*-butyl-4-{[8-(2,3-dihydro-1*H*-cyclopenta[b]quinolin-9-ylamino)-octylimino]-methyl}-phenol (**13d**). Light brown solid; Yield 71%, m.p. 75–78 °C. ^1^H-NMR (CDCl_3_), δ: 1.29–1.40 (m, 6H, 3 × CH_2_), 1.45 (s, 18H, 6 × CH_3_), 1.53–1.78 (m, 6H, 3 × CH_2_), 2.13 (pent, 2H, *J* = 7.2 Hz, CH_2_), 3.07 (t, 2H, *J* = 7.5 Hz, CH_2_), 3.21 (t, 2H, *J* = 7.2 Hz, CH_2_), 3.40–3.76 (m, 4H, 2 × CH_2_), 4.72 (br.s, 1H, NH), 5.45 (br.s, 1H, OH), 7.36 (s, 2 × 1H, 2 × H_ar_), 7.45–7.63 (m, 2H, 2 × H_ar_), 7.74 (d, 1H, *J* = 8.1 Hz, H_ar_), 7.92 (d, 1H, *J* = 8.1 Hz, H_ar_), 8.11 (s, 1H, =CH). ^13^C-NMR (CDCl_3_), δ: 22.44, 23.23, 26.13, 26.72, 27.30, 29.46, 30.28 (6), 30.81, 31.19, 34.26, 35.00, 45.72, 54.48, 114.02, 118.82, 119.56, 123.82, 124.81, 125.04, 128.10, 129.17, 131.07, 135.77, 146.28, 148.35, 152.68, 168.63. Anal. Calcd. for C_35_H_49_N_3_O: C, 79.65; H, 9.36; N, 7.96. Found: C, 79.53; H, 9.27; N, 8.05.

2,6-Di-*tert*-butyl-4-{[4-(1,2,3,4-tetrahydro-acridin-9-ylamino)-butylimino]-methyl}-phenol (**14b**). Yellow solid; Yield 67%, m.p. 90–93 °C. ^1^H-NMR (CDCl_3_), δ: 1.45 (s, 18H, 6 × CH_3_), 1.64–1.83 (m, 4H, 2 × CH_2_), 1.83–2.07 (m, 4H, 2 × CH_2_), 2.56–2.80 (m, 2H, CH_2_), 2.91–3.18 (m, 2H, CH_2_), 3.36–3.75 (m, 4H, 2 × CH_2_), 4.11 (br.s, 1H, NH), 5.51 (br.s, 1H, OH), 7.21–7.32 (m, 1H, H_ar_), 7.41–7.70 (m, 1H, H_ar_), 7.55 (s, 2 × 1H, 2 × H_ar_), 7.90 (d, 1H, *J* = 8.8 Hz, H_ar_), 7.96 (d, 1H, *J* = 8.8 Hz, H_ar_), 8.19 (s, 1H, =CH). ^13^C-NMR (CDCl_3_), δ: 22.77, 23.04, 24.86, 28.47, 29.51, 30.16 (6), 34.04, 34.33 (2), 49.25, 61.15, 115.81, 120.19, 122.82, 123.53, 125.25 (2), 127.64, 128.20, 128.71, 136.14 (2), 147.45, 150.68, 156.27, 158.41, 161.73. Anal. Calcd. for C_32_H_43_N_3_O: C, 79.13; H, 8.92; N, 8.65. Found: C, 79.28; H, 8.84; N, 8.53.

2,6-Di-*tert*-butyl-4-{[6-(1,2,3,4-tetrahydro-acridin-9-ylamino)-hexylimino]-methyl}-phenol (**14c**). Yellow solid; Yield 65%, m.p. 79–81 °C. ^1^H-NMR (CDCl_3_), δ: 1.30–1.40 (m, 4H, 2 × CH_2_), 1.46 (s, 18H, 6 × CH_3_), 1.66–1.87 (m, 4H, 2 × CH_2_), 1.83–2.10 (m, 4H, 2 × CH_2_), 2.54–2.82 (m, 2H, CH_2_), 2.96–3.16 (m, 2H, CH_2_), 3.37–3.78 (m, 4H, 2 × CH_2_), 4.11 (br.s, 1H, NH), 5.51 (br.s, 1H, OH), 7.23–7.32 (m, 1H, H_ar_), 7.44–7.75 (m, 1H, H_ar_), 7.58 (s, 2 × 1H, 2 × H_ar_), 7.91 (d, 1H, *J* = 8.8 Hz, H_ar_), 7.98 (d, 1H, *J* = 8.8 Hz, H_ar_), 8.18 (s, 1H, =CH). ^13^C-NMR (CDCl_3_), δ: 21.76, 22.94, 24.26, 27.11, 27.67, 28.57, 29.78, 30.11 (6), 33.93, 34.26 (2), 48.12, 60.12, 119.28, 120.51, 123.13, 124.37, 125.66, 127.62, 128.16, 128.55, 128.22, 137.13 (2), 146.81, 151.13, 160.02, 163.75. Anal. Calcd. for C_34_H_47_N_3_O: C, 79.49; H, 9.22; N, 8.18. Found: C, 79.38; H, 9.30; N, 8.27.

2,6-Di-*tert*-butyl-4-{[8-(1,2,3,4-tetrahydro-acridin-9-ylamino)-octylimino]-methyl}-phenol (**14d**). Yellow solid; Yield 65%, m.p. 68–69 °C. ^1^H-NMR (CDCl_3_), δ: 1.20–1.37 (m, 8H, 4 × CH_2_), 1.44 (s, 18H, 6 × CH_3_), 1.56–1.74 (m, 4H, 2 × CH_2_), 1.82–2.02 (m, 4H, 2 × CH_2_), 2.59–2.80 (m, 2H, CH_2_), 2.98–3.15 (m, 2H, CH_2_), 3.38–3.68 (m, 4H, 2 × CH_2_), 4.11 (br.s, 1H, NH), 5.45 (br.s, 1H, OH), 7.36 (s, 2 × 1H, 2 × H_ar_), 7.44–7.69 (m, 3H, =CH, 2 × H_ar_), 7.94 (t, 2H, *J* = 8.2 Hz, 2 × H_ar_). ^13^C-NMR (CDCl_3_), δ: 22.43, 22.69, 22.99, 24.61, 24.71, 26.85, 27.11, 29.30, 30.10 (6), 31.02, 31.73, 33.80, 34.38, 49.45, 115.59, 120.03, 122.87, 123.58, 125.30, 125.49, 127.61, 128.38, 136.69, 147.13, 150.94, 158.12, 161.45. Anal. Calcd. for C_36_H_51_N_3_O: C, 79.80; H, 9.49; N, 7.76. Found: C, 79.68; H, 9.39; N, 7.85.

2,6-Di-*tert*-butyl-4-{[4-(7,8,9,10-tetrahydro-6*H-*cyclohepta[b]quinolin-11-ylamino)-butylimino]-methyl}-phenol (**15b**). Light yellow solid; Yield 68%. m.p. 138–140 °C. ^1^H-NMR (CDCl_3_), δ: 1.45 (s, 18H, 6 × CH_3_), 1.59–1.96 (m, 10H, 5 × CH_2_), 2.79–3.00 (m, 2H, CH_2_), 3.08–3.23 (m, 2H, CH_2_), 3.35 (t, 2H, *J* = 6.6 Hz, CH_2_), 3.60 (t, 2H, *J* = 5.6 Hz, CH_2_), 5.50 (br.s, 1H, OH), 7.30–7.45 (m, 1H, H_ar_), 7.54 (s, 2 × 1H, 2 × H_ar_), 7.49–7.66 (m, 1H, H_ar_), 7.93 (t, 2H, *J* = 9.2 Hz, 2 × H_ar_), 8.16 (s, 1H, =CH). ^13^C-NMR (CDCl_3_), δ: 26.85, 27.68, 28.26, 28.58, 29.22, 30.14 (6), 31.97, 34.34, 39.88, 50.47, 65.82, 121.94, 120.00, 123.75, 124.70, 124.83, 125.29, 128.27, 128.83, 129.20, 136.31, 146.46, 149.88, 161.56, 165.16. Anal. Calcd. for C_33_H_45_N_3_O: C, 79.31; H, 9.08; N, 8.41. Found: C, 79.45; H, 9.15; N, 8.31.

2,6-Di-*tert*-butyl-4-{[6-(7,8,9,10-tetrahydro-6*H*-cyclohepta[b]quinolin-11-ylamino)-hexylimino]-methyl}-phenol (**15c**). Light yellow solid; Yield 68%, m.p. 62–65 °C. ^1^H-NMR (CDCl_3_), δ: 1.21–1.33 (m, 2H, CH_2_), 1.44 (s, 18H, 6 × CH_3_), 1.58–1.91 (m, 12H, 6 × CH_2_), 2.84–2.97 (m, 2H, CH_2_), 3.17–3.28 (m, 2H, CH_2_), 3.29–3.41 (m, 2H, CH_2_), 3.54 (t, 2H, *J* = 7.1 Hz, CH_2_), 4.27 (br.s, 1H, NH), 5.47 (br.s, 1H, OH), 7.41 (t, 1H, *J* = 7.5 Hz, H_ar_), 7.51 (s, 2 × 1H, 2 × H_ar_), 7.59 (t, 1H, *J* = 7.5 Hz, H_ar_), 7.93 (d, 1H, *J* = 8.4 Hz, H_ar_), 8.05 (d, 1H, *J* = 8.4 Hz, H_ar_), 8.07 (s, 1H, =CH). ^13^C-NMR (CDCl_3_), δ: 27.19, 27.25, 27.46, 28.03, 28.52, 30.58 (6), 31.39, 31.83, 32.33, 34.85, 39.55, 50.91, 60.87, 121.87, 122.50, 123.36, 125.36, 125.95, 127.61, 128.10, 128.35, 129.22, 137.23, 145.84, 151.03, 161.04, 164.85. Anal. Calcd. for C_35_H_49_N_3_O: C, 79.65; H, 9.36; N, 7.96. Found: C, 79.52; H, 9.45; N, 7.88.

2,6-Di-*tert*-butyl-4-{[8-(7,8,9,10-tetrahydro-6*H*-cyclohepta[b]quinolin-11-ylamino)-octylimino]-methyl}-phenol (**15d**). Light yellow solid; Yield 66%, m.p. 77–78 °C. ^1^H-NMR (CDCl_3_), δ: 1.18–1.39 (m, 6H, 3 × CH_2_), 1.44 (s, 18H, 6 × CH_3_), 1.55–1.97 (m, 12H, 6 × CH_2_), 2.85–3.00 (m, 2H, CH_2_), 3.13–3.34 (m, 4H, 2 × CH_2_), 3.52 (t, 2H, *J* = 7.1 Hz, CH_2_), 4.07 (br.s, 1H, NH), 5.49 (br.s, 1H, OH), 7.32–7.65 (m, 4H, 4 × H_ar_), 7.83–8.15 (m, 3H, 2 × H_ar_, =CH). ^13^C-NMR (CDCl_3_), δ: 26.84, 26.93, 27.13, 27.64, 28.23, 29.14, 29.27, 29.31, 30.11 (6), 31.02, 31.45, 31.95, 34.37, 39.85, 50.66, 121.87, 123.71, 124.70, 125.37, 128.30, 128.80, 146.45, 149.96, 160.32, 165.11. Anal. Calcd. for C_37_H_53_N_3_O: C, 79.95; H, 9.61; N, 7.56. Found: C, 79.85; H, 9.54; N, 7.68.

2,6-Di-*tert*-butyl-4-{[4-(2,3-dihydro-1*H*-cyclopenta[b]quinolin-9-ylamino)-butylamino]-methyl}-phenol (**16b**). Light yellow solid; Yield 65%, m.p. 65–67 °C. ^1^H-NMR (CDCl_3_), δ: 1.44 (s, 18H, 6 × CH_3_), 1.62–1.82 (m, 4H, 2 × CH_2_), 2.13 (pent, 2H, *J* = 7.1 Hz, CH_2_), 2.66 (t, 2H, *J* = 7.2 Hz, CH_2_), 3.05 (t, 2H, *J* = 6.8 Hz, CH_2_), 3.28 (t, 2H, *J* = 6.8 Hz, CH_2_), 3.62 (кв, 2H, *J* = 6.3 Hz, CH_2_), 3.68 (s, 2H, CH_2_), 4.64 (br.s, 1H, NH), 5.15 (br.s, 1H, OH), 7.10 (s, 2 × 1H, 2 × H_ar_), 7.55 (t, 1H, *J* = 7.2 Hz, H_ar_), 7.55 (t, 1H, *J* = 7.2 Hz, H_ar_), 7.75 (t, 1H, *J* = 8.1 Hz, H_ar_), 7.90 (d, 1H, *J* = 8.0 Hz, H_ar_). Anal. Calcd. for C_31_H_43_N_3_O: C, 78.60; H, 9.15; N, 8.87. Found: C, 78.72; H, 9.06; N, 8.78.

2,6-Di-*tert*-butyl-4-{[6-(2,3-dihydro-1*H*-cyclopenta[b]quinolin-9-ylamino)-hexylamino]-methyl}-phenol (**16c**). Light yellow solid; Yield 75%, m.p. 62–65 °C. ^1^H-NMR (CDCl_3_), δ: 1.16–1.33 (m, 4H, CH_2_), 1.42 (s, 18H, 6 × CH_3_), 1.51–1.75 (m, 6H, 3 × CH_2_), 2.11 (pent, 2H, *J* = 7.2 Hz, CH_2_), 2.65 (t, 2H, *J* = 5.9 Hz, CH_2_), 3.03 (t, 2H, *J* = 7.2 Hz, CH_2_), 3.18 (t, 2H, *J* = 6.7 Hz, CH_2_), 3.47–3.62 (m, 2H, CH_2_), 3.66 (s, 2H, CH_2_), 4.62 (br.s, 1H, NH), 5.11 (br.s, 1H, OH), 7.08 (s, 2 × 1H, 2 × H_ar_), 7.38 (t, 1H, *J* = 7.5 Hz, H_ar_), 7.57 (t, 1H, *J* = 7.4 Hz, H_ar_), 7.70 (d, 1H, *J* = 8.2 Hz, H_ar_), 7.88 (d, 1H, *J* = 8.2 Hz, H_ar_). ^13^C-NMR (CDCl_3_), δ: 22.42, 23.21, 26.10, 26.71, 27.13, 30.27 (6), 30.79, 34.25, 34.97, 45.66, 49.60, 54.48, 114.05, 118.84, 119.56, 123.82, 124.76, 128.08, 129.17, 131.04, 135.82, 146.26, 148.36, 152.69, 168.61. Anal. Calcd. for C_33_H_47_N_3_O: C, 79.00; H, 9.44; N, 8.37. Found: C, 79.13; H, 9.08; N, 8.45.

2,6-Di-*tert*-butyl-4-{[8-(2,3-dihydro-1*H*-cyclopenta[b]quinolin-9-ylamino)-octylamino]-methyl}-phenol (**16d**). Light grey solid; Yield 76%, m.p. 60–63 °C. ^1^H-NMR (CDCl_3_), δ: 1.27–1.38 (m, 8H, 4 × CH_2_), 1.44 (s, 18H, 6 × CH_3_), 1.53–1.77 (m, 4H, 2 × CH_2_), 2.13 (pent, 2H, *J* = 7.1 Hz, CH_2_), 2.65 (t, 2H, *J* = 7.4 Hz, CH_2_), 3.05 (t, 2H, *J* = 7.5 Hz, CH_2_), 3.21 (t, 2H, *J* = 7.1 Hz, CH_2_), 3.63 (кв, 2H, *J* = 6.2 Hz, CH_2_), 3.75 (s, 2H, CH_2_), 4.63 (br.s, 1H, NH), 5.14 (br.s, 1H, OH), 7.10 (s, 2 × 1H, 2 × H_ar_), 7.36 (t, 1H, *J* = 7.5 Hz, H_ar_), 7.55 (t, 1H, *J* = 7.5 Hz, H_ar_), 7.75 (d, 1H, *J* = 8.2 Hz, H_ar_), 7.90 (d, 1H, *J* = 8.1 Hz, H_ar_). Anal. Calcd. for C_35_H_51_N_3_O: C, 79.35; H, 9.70; N, 7.93. Found: C, 79.23; H, 9.78; N, 8.02.

2,6-Di-*tert*-butyl-4-{[2-(1,2,3,4-tetrahydro-acridin-9-ylamino)-ethylamino]-methyl}-phenol (**17a**). Yellow solid; Yield 76%. m.p. 66–68°C. ^1^H-NMR (CDCl_3_) δ: 1.45 (s, 18H, 6 × CH_3_), 1.83–2.02 (m, 4H, 2 × CH_2_), 2.70–2.84 (m, 2H, CH_2_), 2.86–2.99 (m, 2H, CH_2_), 3.00–3.13 (m, 2H, CH_2_), 3.53–3.66 (m, 2H, CH_2_), 3.74 (s, 2H, CH_2_), 5.18 (br.s, 1H, OH), 7.16 (s, 2H, 2 × H_ar_), 7.32 (t, 1H, *J* = 7.6 Hz, H_ar_), 7.54 (t, 1H, *J* = 7.6 Hz, H_ar_), 7.89 (d, 1H, *J* = 8.4 Hz, H_ar_), 8.03 (d, 1H, *J* = 8.4 Hz, H_ar_). ^13^C-NMR (CDCl_3_), δ: 22.82, 23.10, 24.82, 30.27 (6), 33.99, 34.28, 48.15, 49.39, 53.89, 116.02, 120.32, 122.89, 123.42, 124.76, 128.11, 128.64, 130.69, 135.92, 147.44, 150.96, 152.85, 158.38. Anal. Calcd. for C_30_H_41_N_3_O: C, 78.39; H, 8.99; N, 9.14. Found: C, 78.28; H, 8.91; N, 9.03.

2,6-Di-*tert*-butyl-4-{[4-(1,2,3,4-tetrahydro-acridin-9-ylamino)-butylamino]-methyl}-phenol (**17b**). Light grey solid; Yield 67%, m.p. 59–62 °C. ^1^H-NMR (CDCl_3_), δ: 1.42 (s, 18H, 6 × CH_3_), 1.55–1.80 (m, 4H, 2 × CH_2_), 1.81–2.02 (m, 4H, 2 × CH_2_), 2.49–2.85 (m, 4H, 2 × CH_2_), 2.90–3.19 (m, 2H, CH_2_), 3.34–3.60 (m, 2H, CH_2_), 3.67 (c, 2H, CH_2_), 4.02 (br.s, 1H, NH), 5.14 (br.s, 1H, OH), 7.08 (s, 2 × 1H, 2 × H_ar_), 7.31 (t, 1H, *J* = 7.8 Hz, H_ar_), 7.54 (t, 1H, *J* = 7.8 Hz, H_ar_), 7.77–8.03 (m, 2H, 2 × H_ar_). ^13^C-NMR (CDCl_3_), δ: 22.79, 23.06, 24.87, 27.58, 29.62, 30.28 (6), 34.09, 34.28 (2), 49.28, 49.44, 54.46, 115.91, 120.23, 122.78, 123.54, 124.77 (2), 128.19, 128.78 (2), 130.89, 135.82, 147.51, 150.65, 152.75, 158.46. Anal. Calcd. for C_32_H_45_N_3_O: C, 78.80; H, 9.30; N, 8.62. Found: C, 78.68; H, 9.39; N, 8.54.

2,6-Di-*tert*-butyl-4-{[6-(1,2,3,4-tetrahydro-acridin-9-ylamino)-hexylamino]-methyl}-phenol (**17c**). Light grey solid; Yield 68%, m.p. 68–70 °C. ^1^H-NMR (CDCl_3_), δ: 1.20–1.30 (m, 8H, 4 × CH_2_), 1.41 (s, 18H, 6 × CH_3_), 1.50–1.70 (m, 4H, 2 × CH_2_), 1.84–2.05 (m, 4H, 2 × CH_2_), 2.47–2.86 (m, 4H, 2 × CH_2_), 2.97–3.19 (m, 2H, CH_2_), 3.44–3.57 (m, 2H, CH_2_), 3.71 (s, 2H, CH_2_), 4.10 (br.s, 1H, NH), 5.12 (br.s, 1H, OH), 7.13 (s, 2 × 1H, 2 × H_ar_), 7.36 (t, H, *J* = 8.2 Hz, H_ar_), 7.54 (t, H, *J* = 6.8 Hz, H_ar_), 7.95 (t, 2H, *J* = 8.3 Hz, 2 × H_ar_). ^13^C-NMR (CDCl_3_), δ: 22.39, 23.26, 24.81, 27.19, 27.55, 28.51, 29.88, 30.27 (6), 34.19, 34.22 (2), 49.21, 49.54, 55.48, 118.92, 121.21, 122.76, 124.55, 123.71 (2), 128.34, 126.72 (2), 131.19, 136.12, 146.53, 151.05, 161.24, 165.05. Anal. Calcd. for C_34_H_49_N_3_O: C, 79.18; H, 9.58; N, 8.15. Found: C, 79.07; H, 9.49; N, 8.24.

2,6-Di-*tert*-butyl-4-{[8-(1,2,3,4-tetrahydro-acridin-9-ylamino)-octylamino]-methyl}-phenol (**17d**). Yellow solid; Yield 75%, m.p. 71–73 °C. ^1^H-NMR (CDCl_3_), δ: 1.23–1.37 (m, 8H, 4 × CH_2_), 1.43 (s, 18H, 6 × CH_3_), 1.53–1.75 (m, 4H, 2 × CH_2_), 1.82–2.04 (m, 4H, 2 × CH_2_), 2.49–2.89 (m, 4H, 2 × CH_2_), 2.99–3.17 (m, 2H, CH_2_), 3.41–3.58 (m, 2H, CH_2_), 3.70 (s, 2H, CH_2_), 4.12 (br.s, 1H, NH), 5.14 (br.s, 1H, OH), 7.12 (s, 2 × 1H, 2 × H_ar_), 7.34 (t, H, *J* = 8.1 Hz, H_ar_), 7.55 (t, H, *J* = 6.9 Hz, H_ar_), 7.95 (t, 2H, *J* = 8.3 Hz, 2 × H_ar_). ^13^C-NMR (CDCl_3_), δ: 22.40, 22.57, 22.91, 24.63, 26.79, 27.18, 29.21, 29.34, 29.62, 30.23 (6), 30.85, 31.64, 33.55, 34.24, 49.38, 115.35, 119.81, 122.90, 123.59, 124.99, 128.06, 128.48, 130.66, 135.33, 135.80, 146.74, 151.06, 157.82. Anal. Calcd. for C_36_H_53_N_3_O: C, 79.51; H, 9.82; N, 7.73. Found: C, 79.64; H, 9.90; N, 7.83.

2,6-Di-*tert*-butyl-4-{[4-(7,8,9,10-tetrahydro-6*H*-cyclohepta[b]quinolin-11-ylamino)-butylamino]-methyl}-phenol (**18b**). Light grey solid; Yield 64%, m.p. 65–67 °C. ^1^H-NMR (CDCl_3_), δ: 1.43 (s, 18H, 6 × CH_3_), 1.60–1.89 (m, 10H, 5 × CH_2_), 2.71 (t, 2H, *J* = 6.3 Hz, CH_2_), 2.82–2.99 (m, 2H, CH_2_), 3.10–3.23 (m, 2H, CH_2_), 3.30 (t, 2H, *J* = 6.8 Hz, CH_2_), 3.69 (c, 2H, CH_2_), 5.14 (br.s, 1H, OH), 7.10 (s, 2 × 1H, 2 × H_ar_), 7.33–7.46 (m, 1H, H_ar_), 7.57 (t, 1H, *J* = 7.8 Hz, H_ar_), 7.92 (t, 2H, *J* = 8.7 Hz, 2 × H_ar_). ^13^C-NMR (CDCl_3_), δ: 26.90, 27.70 (2), 28.31, 29.33, 30.29 (6), 32.00, 34.28, 40.13 (2), 49.33, 50.68, 54.47, 121.86, 124.02, 124.67, 124.81 (2), 125.04, 128.17, 129.12 (2), 130.87, 135.84, 146.79, 149.72, 152.77, 165.38. Anal. Calcd. for C_33_H_47_N_3_O: C, 79.00; H, 9.44; N, 8.37. Found: C, 79.14; H, 9.35; N, 8.43.

2,6-Di-*tert*-butyl-4-{[6-(7,8,9,10-tetrahydro-6*H*-cyclohepta[b]quinolin-11-ylamino)-hexylamino]-methyl}-phenol (**18c**). White powder; Yield 68%, m.p. 71–73 °C. ^1^H-NMR (CDCl_3_), δ: 1.12–1.32 (m, 4H, 2 × CH_2_), 1.43 (s, 18H, 6 × CH_3_), 1.55–1.98 (m, 10H, 5 × CH_2_), 2.66 (t, 2H, *J* = 6.7 Hz, H_ar_), 2.84–3.01 (m, 2H, CH_2_), 3.11–3.36 (m, 4H, 2 × CH_2_), 3.69 (s, 2H, CH_2_), 5.17 (br.s, 1H, OH), 7.11 (s, 2 × 1H, 2 × H_ar_), 7.41 (t, 1H, *J* = 7.7 Hz, H_ar_), 7.58 (t, 1H, *J* = 7.2 Hz, H_ar_), 7.89 (d, 1H, *J* = 8.4 Hz, H_ar_), 7.95 (d, 1H, *J* = 8.4 Hz, H_ar_). ^13^C-NMR (CDCl_3_), δ: 26.86, 27.14, 27.64, 28.25, 29.66, 30.26 (6), 31.40, 31.97, 34.27, 40.00, 49.33, 50.66, 54.22, 60.36, 121.85, 122.05, 123.97, 124.68, 124.98, 128.21, 128.98, 135.36, 135.82, 146.63, 149.75, 152.84, 165.26. Anal. Calcd. for C_35_H_51_N_3_O: C, 79.35; H, 9.70; N, 7.93. Found: C, 79.49; H, 9.59; N, 7.86.

2,6-Di-*tert*-butyl-4-{[8-(7,8,9,10-tetrahydro-6*H*-cyclohepta[b]quinolin-11-ylamino)-octylamino]-methyl}-phenol (**18d**). White powder; Yield 76%, m.p. 63–65 °C. ^1^H-NMR (CDCl_3_), δ: 1.16–1.38 (m, 8H, 4 × CH_2_), 1.44 (s, 18H, 6 × CH_3_), 1.57–1.99 (m, 10H, 5 × CH_2_), 2.66 (t, 2H, *J* = 7.2 Hz, CH_2_), 2.85–2.99 (m, 2H, CH_2_), 3.12–3.33 (m, 4H, 2 × CH_2_), 3.69 (s, 2H, CH_2_), 5.13 (br.s, 1H, OH), 7.15 (s, 2 × 1H, 2 × H_ar_), 7.41(t, 1H, *J* = 7.5 Hz, H_ar_), 7.57 (t, 1H, *J* = 7.5 Hz, H_ar_), 7.92 (t, 2H, *J* = 9.2 Hz, 2 × H_ar_),). ^13^C-NMR (CDCl_3_), δ: 26.85, 27.62, 28.26, 29.30, 29.40, 29.55, 30.33 (6), 30.84, 31.42, 31.95, 34.20, 40.08, 49.57, 50.70, 54.32, 58.32, 121.82, 123.96, 124.59, 124.84, 125.00, 128.10, 129.07, 130.71, 135.36, 135.80, 146.76, 149.69, 165.30. Anal. Calcd. for C_37_H_55_N_3_O: C, 79.66; H, 9.94; N, 7.53. Found: C, 79.78; H, 9.84; N, 7.62.

### 3.2. Biological Testing

#### 3.2.1. In Vitro AChE, BChE and CES Inhibition 

All experiments were carried out in accordance with the standard protocols approved by IPAC RAS.

Human erythrocyte AChE and equine serum BChE were purchased from Milamed (Perm, Russia). Porcine liver CES, substrates, and reference compounds were from Sigma-Aldrich (St. Louis, MO, USA). The activity of enzymes was measured spectrophotometrically, as described in detail in [72] using ATCh iodide, BTCh iodide, and 4-NPA as substrates for AChE, BChE, and CES, respectively. Experimental conditions: K,Na—phosphate buffer (100 mM), 25 °C, pH 7.5 for AChE and BChE and pH 8.0 for CES assay. Measurements were carried out on a FLUOStar Optima microplate reader (BMG Labtech, Ortenberg, Germany).

Test compounds were dissolved in DMSO; the final concentration of solvent in the incubation mixture was 2% (*v*/*v*). An initial assessment of inhibitory activity was carried out by determining the degree of enzyme inhibition at a compound concentration of 20 µM. For active compounds (inhibition ≥ 35%), IC_50_ values were determined.

A mechanism of AChE and BChE inhibition was assessed by a detailed analysis of enzyme kinetics with three increasing concentrations of inhibitor and six substrate concentrations, as described in detail in [72]. 

#### 3.2.2. Propidium Displacement from *Ee*AChE PAS

The ability of the test compounds to competitively displace propidium, a selective ligand of the PAS of AChE, was evaluated by the fluorescence method [73,74], as described in detail in [43]. Propidium iodide, donepezil, and Electric eel AChE (*Ee*AChE, type VI-S, lyophilized powder) were purchased from Sigma-Aldrich (Saint Louis, MO, USA). 

After 15 min of incubation of the test compounds at a concentration of 20 μM with a 7 μM solution of *Ee*AChE in 1 mM Tris-HCl buffer, pH 8.0, 25 °C, propidium iodide (final concentration 8 μM) was added. Then, the solutions were incubated for 15 min and the fluorescence spectrum was recorded (530 nm (excitation) and 600 nm (emission)). Donepezil and tacrine were the reference compounds. Measurements were performed in triplicate on a FLUOStar Optima microplate reader (BMG LabTech, Ortenberg, Germany).

#### 3.2.3. Effect on β-Amyloid Self-Aggregation

The inhibitory effect of the test compounds toward Aβ_42_ self-aggregation was determined using the thioflavin T (ThT) fluorescence method [24,27,75] with minor modifications, as described in detail in [38]. Lyophilized HFIP-pretreated Aβ_42_ from BACHEM (Bubendorf, Switzerland) was used.

For the measurement of Aβ_42_ self-aggregation and the assessment of the inhibition of amyloid fibril formation by the tested compounds, aliquots of 500 μM Aβ_42_ stock solution in DMSO were diluted in 215 mM Na-phosphate buffer pH 8.0 to a final concentration of 50 μM Aβ_42_ and incubated for 24 h at 37 °C in the absence or presence of the tested compounds at a concentration of 100 µM. After that, the samples were incubated with 5 μM ThT in 50 mM glycine-NaOH buffer pH 8.5 for 10 min and the fluorescence was measured at 440 nm (excitation) and 485 nm (emission). Myricetin and propidium iodide were used as reference compounds (positive controls). Analyses were performed with a FLUOStar Optima microplate reader (BMG LabTech, Ortenberg, Germany). 

#### 3.2.4. Antioxidant Activity

##### ABTS Radical Cation Scavenging Activity Assay

Radical scavenging activity of the compounds was evaluated using the ABTS radical cation (2,2′-azinobis-(3-ethylbenzothiazoline-6-sulfonic acid, ABTS^•+^) decolorization assay [76] with minor modifications, as described in detail in [77]. The reduction in absorbance was measured spectrophotometrically at 734 nm using a xMark UV/VIS microplate spectrophotometer (Bio-Rad, Hercules, CA, USA) for 1 h with an interval of 1–10 min compared to a standard synthetic antioxidant, Trolox (6-hydroxy-2,5,7,8- tetramethylchroman-2-carboxylic acid). 

The antioxidant activity of the compounds was reported as the Trolox equivalent antioxidant capacity (TEAC values)—the ratio of the slopes of the concentration−response curves, test compound/Trolox. The IC_50_ values for the test compounds (compound concentration required for 50% reduction of the ABTS radical) were also determined.

##### Ferric-Reducing Antioxidant Power (FRAP) Assay

The ferric-reducing antioxidant power (FRAP) assay proposed by Benzie and Strain [78,79] and modified to be performed in 96-well microplates, as described in detail in [43], was used. A total of 10 µL (0.5 mM) of the tested compound or reference compound were mixed with 240 µL of the FRAP reagent, and the absorbance of the mixture was measured spectrophotometrically (λ = 593 nm) with a FLUOStar OPTIMA microplate reader (BMG LabTech, Ortenberg, Germany) at 600 nm, after a 1 h incubation at 37 °C against a blank. Trolox was used as a reference compound. The results were expressed as Trolox equivalents (TE)—the ratio of the concentrations of Trolox and the test compound resulting in the same effect on ferric-reducing activity.

##### Tissue Preparation

All procedures on animals were carried out in accordance with the EU Directive 2010/63/EU. Hybrid BDF1 mice were killed by decapitation using a guillotine. Each brain was immediately removed, frozen and stored at −80 °C until use. The brains were homogenized using a Wisd WiseTis HG-15D homogenizer (Daihan Scientific, Wonju, South Korea) in 0.01 M PBS (pH 7.4) or 0.1 M Tris-HCl (pH 7.4). The Lowry assay was used for accurate protein determination [80].

##### Luminol Chemiluminescence Assay of Radical-Scavenging Activity of Conjugates in Mouse Brain Homogenate

Reactive oxygen species production was measured by luminol-dependent chemiluminescence in mouse brain homogenates using the Luminometer (model 1250, LKB Wallac, Turku, Finland) according to the known method [52], with minor changes [43]. The reaction mixture contained homogenate (protein concentration 0.1 mg/mL) in 0.1 M Tris-HCl (Sigma-Aldrich) with a pH of 7.4, luminol (Sigma-Aldrich, Merck KGaA, Darmstadt, Germany) (0.05 mmol/L), tert-butyl hydroperoxide (TBHP) (Sigma-Aldrich, Merck KGaA, Darmstadt, Germany) (0.073 mol/L) and tested compounds. Compounds were dissolved in DMSO (Sigma-Aldrich, Merck KGaA, Darmstadt, Germany). For a statistical analysis of chemiluminescence measurements, the light production was assessed as the area under the chemiluminescence curve and compared to control studies whose chemiluminescence response was set at 100%. All measurements were always conducted in triplicates and values expressed as the mean ± standard deviation (SD). IC_50_ values represent the concentration that caused a 50% reduction in luminescence.

##### TBARS Assay of the Evaluation of Spontaneous LP Level

The assay was carried out in accordance with the method of Ohkawa et al. [53], with minor changes as described in detail in [43]. Briefly, mouse brain homogenate (protein concentration 1 mg/mL) in PBS (0.1 M, pH 7.4) was incubated for 30 min at 37 °C with tested conjugates, and the reaction was terminated by adding 17% (*w*/*v*) trichloroacetic acid. The assay mixture was maintained at +4 °C to ensure the complete precipitation of all proteins; then, the samples were centrifuged for 20 min at 1300× *g* in a microcentrifuge Frontier Micro (OHAUS Europe GmbH, Greifensee, Switzerland). TBARs in supernatant were determined by adding 0.5 mL of 0.8% (*w*/*v*) of TBA (Sigma-Aldrich, Merck KGaA, Darmstadt, Germany) and heating for 30 min at 95 °C. The optical density was measured at 532 nm against a blank using a Cary 60 UV-Vis spectrophotometer (Agilent, Santa Clara, CA, USA); Trolox and BHT were used as reference antioxidants. Compounds were dissolved in DMSO (Sigma-Aldrich, Merck KGaA, Darmstadt, Germany) and tested in a concentration range of 0.01–100 μM. IC_50_ values represent the concentration that caused the 50% inhibition of LP.

### 3.3. Molecular Modeling Studies 

#### 3.3.1. Molecular Docking 

The Calculator Plugins of MarvinSketch 21.14.0, ChemAxon (https://www.chemaxon.com, accessed on 27 January 2023) and MolGpKa [81] (https://xundrug.cn/molgpka, accessed on 27 January 2023) were used to estimate the p*K*_a_ values of the ligands. The ligand compounds were optimized using a DFT quantum chemistry method (B3LYP/6-31G*, GAMESS-US [82] software, https://www.msg.chem.iastate.edu/gamess/, accessed on 1 November 2023). For molecular docking, the optimized structures of the ligands were employed, with partial atomic charges obtained from QM results based on the Löwdin scheme [83]. 

The protein targets used for docking included X-ray structures of human AChE co-crystallized with donepezil (PDB: 4EY7, chain A) [84], and an optimized X-ray structure of human BChE (PDB: 1P0I) [85]. For the modeling of conformational flexibility, all conformers in the NMR structure of the soluble α-helical form of Aβ_42_ PDB ID 1IYT [86] were used.

AutoDock 4.2.6 software [87] was applied to perform molecular docking. The docking grid box was set to cover the entire active site gorge of AChE (22.5 Å × 22.5 Å × 22.5 Å) and BChE (15 Å × 20.25 Å × 18 Å), as well as the entire Aβ_42_ molecule for all conformers (43.5 Å × 28.5 Å × 54.75 Å), with a grid spacing of 0.375 Å used in all cases. The Lamarckian Genetic Algorithm (LGA) [88] was used with 256 runs, 25 × 10^6^ evaluations, 27 × 10^4^ generations, and a population size of 3000. Figures were created using PyMol (www.pymol.org, accessed on 21 July 2016).

#### 3.3.2. QM Calculation of Antioxidant Activity

The quantum chemical calculations were performed by a density-functional theory (DFT) method using Gaussian 16 [89] and Priroda 19 [90,91] packages. The Priroda 19 package was used for a preliminary conformational search in the gas phase with the PBE0 functional [92] and TZVP basis set [93]. The lowest-energy conformations were used as initial geometries for optimization in solvent (water or ethanol) by the Gaussian 16 package. The optimization was conducted using the B3LYP functional [94,95] and 6-31++G(d,p) basis set [96] with the empirical Grimme correction DFT-D3BJ [97]. The solvent effects were taken into account using the SMD continuum solvation model [98]. 

#### 3.3.3. Prediction of ADMET, Physicochemical, and PAINS Profiles

Lipophilicity (LogP_ow_) and aqueous solubility (pS_aq_) were estimated by the ALogPS 3.0 neural network model implemented in the OCHEM platform [99]. The human intestinal absorption (HIA) [100], blood–brain barrier distribution/permeability (LogBB) [101,102] and hERG-mediated cardiac toxicity risk (channel affinity p*K_i_* and inhibitory activity pIC_50_) [103] were estimated using the integrated online service for the prediction of ADMET properties [104] that employs predictive QSAR models based on fragmental descriptors, artificial neural networks and accurate and representative training sets. The quantitative estimate of drug-likeness (QED) values [105] were calculated and the Pan Assay INterference compoundS (PAINS) alerts were checked using RDKit version 2021.09.2 software [106].

### 3.4. Statistical Analyses

All tests were performed with a minimum of three internal replicates in at least three independent experiments. Results are presented as mean ± SEM or mean ± SD, calculated using GraphPad Prism version 6.05 for Windows (San Diego, CA, USA). Plots, linear regressions, and IC_50_ values were determined using Origin 6.1 for Windows, OriginLab (Northampton, MA, USA). The statistical significance of differences between test and control means was determined by a standard one-way ANOVA and Dunnett’s post hoc test when the variances were statistically equivalent. When the variances were statistically different, the Brown–Forsythe and Welch one-way ANOVA and Dunnett’s T3 post hoc test were used. Results for inhibition of Aβ_42_ self-aggregation were analyzed by one-way ANOVA and the Šídák post hoc test with preselected contrasts between myricetin and propidium iodide vs. all other compounds. All ANOVA methods employed an alpha level of 0.05, and significance levels were designated as follows: * *p* < 0.033; ** *p* < 0.002; *** *p* < 0.001 (GraphPad Prism 10.1.1 for Windows, San Diego, CA, USA). 

## 4. Conclusions

In summary, we first synthesized two series of new conjugates of 4-amino-2,3-polymethylenequinolines and butylated hydroxytoluene linked together with alkylimine and alkylamine spacers. In contrast to our previous studies [44], the present work employed spacers of variable length. Next, we assessed biological activities of the compounds according to their potential as multi-target agents for AD treatment. These assessments included new or more detailed biological characterizations than those that were conducted in our previous investigations and revealed substantial improvements in anticholinesterase properties.

All conjugates were potent inhibitors of AChE and BChE, with selectivity toward BChE. In contrast, the compounds were weak inhibitors of off-target CES, thereby precluding certain unwanted drug–drug interactions in clinical applications. Hybrids with an alkylamine spacer (**16**–**18**) were more effective at inhibiting AChE and BChE than their alkylimine analogs (**13**–**15**). The maximum inhibition for AChE was achieved by compounds with a cyclohexaquinoline ring and for BChE by compounds with a cycloheptaquinoline ring. The increased spacer length increased activity against both AChE and BChE. Consequently, conjugates **14d** and **17d** showed a maximum activity against AChE (IC_50_ = 0.0171 and 0.0151 μM, respectively), which was ~40 times more active than tacrine. Conjugates **15d** and **18d** exhibited maximum anti-BChE activity (IC_50_ ~6 nM, which was 5 times more active than tacrine. It is also noteworthy that anticholinesterase potencies in the present study were markedly improved over those from the previous investigation. For example, with respect to AChE inhibition, conjugate **14d** from the current work was 350 times more potent than compound **14a** (corresponding to compound **7b** from the previous study).

Patterns of structure–activity relationships were in full agreement with the results of molecular docking. Moreover, kinetics revealed a mixed-type reversible inhibition of AChE and BChE by representative conjugates, and molecular docking results indicated dual binding to the CAS and PAS of AChE. These results, along with experimental data on propidium iodide displacement, suggest their potential to block the AChE-induced β-amyloid aggregation. Conjugates also demonstrated the ability to block β-amyloid self-aggregation; compounds **14d** and **17d** with a hexaquinoline ring (m = 2) and spacer n = 8 were the most active, which agrees with the results of molecular docking to Aβ_42_ for conjugate **17d**. 

High radical-scavenging activity was exhibited by the compounds in the ABTS test. Conjugates with an alkylamine spacer were more active than the alkylimine analogs. Maximum activity (TEAC = 1.2–1.5) was demonstrated by conjugates **16c**, **17b**, **17d**, **18b**, and **18d**. In the FRAP assay, conjugates **13**–**15** and **16**–**18** had a high iron-reducing ability, which, however, was lower (TE = 0.4–0.78) than the activity of BHT. In general, conjugates with an alkylimine spacer were more active than their alkylamine analogs. 

In mouse brain homogenates, conjugates demonstrated a high antioxidant activity. Those with imine spacers were 3–6 times more active than their amine analogs and more active than BHT when assessed by the CL assay. In contrast, in the spontaneous LP assay, AOA of imines was lower than that of amines or BHT.

Quantum–chemical calculations explained the variety of results obtained for the conjugates in various systems for assessing AOA.

Computed ADMET profiles of conjugates showed high predicted values for intestinal absorption enabling their oral administration and favorable blood–brain barrier permeability, suggesting the potential for CNS activity. Thus, pending further development and optimization, the conjugates could be considered promising multifunctional CNS agents for the potential treatment of AD.

## Figures and Tables

**Table 1:** Esterase profiles of the compounds and their ability to displace propidium from the peripheral anionic site of *Electrophorus electricus* AChE (*Ee*AChE).

No	m	n	Inhibitory Activity against AChE, BChE and CES IC_50_, μM or % Inhibition at 20 μM			Propidium Displacement, (%)
			AChE	BChE	CES	
**13a**	1	2	4.86 ± 0.01 ^a,^***	1.92 ± 0.11 ^a,^*	26.1 ± 0.7% ^a,^***	18.1 ± 1.6 ^a,^*^,§^
**13b**	1	4	1.30 ± 0.07 *	0.351 ± 0.001 ***	32.7 ± 1.9% ***	20.0 ± 1.6 ***^,§^
**13c**	1	6	0.210 ± 0.010	0.172 ± 0.017	31.3 ± 2.1% ***	16.5 ± 1.3
**13d**	1	8	0.107 ± 0.009 *^,‡^	0.0417 ± 0.0003 ***	26.3 ± 1.7% ***	15.1 ± 0.9
**14a**	2	2	5.98 ± 0.131 ^a,^***	1.61 ± 0.04 ^a,^**	28.6 ± 1.6% ^a,^***	18.2 ± 1.6 ^a,^*^,§^
**14b**	2	4	0.424 ± 0.022	0.385 ± 0.031 *	26.6 ± 2.2% ***	17.6 ± 1.4 *^,§^
**14c**	2	6	0.0712 ± 0.0012 *^,‡^	0.055 ± 0.005	25.1 ± 1.9% ***	19.7 ± 1.5 **^,§^
**14d**	2	8	**0.0171 ± 0.0016** *^,‡^	0.00939 ± 0.00042 ***^,‡^	28.8 ± 2.0% ***	14.8 ± 1.0
**15a**	3	2	4.03 ± 0.03 ^a,^***	0.419 ± 0.040 ^a^	30.1 ± 2.5% ^a,^***	16.3 ± 1.3 ^a^
**15b**	3	4	0.524 ± 0.020	0.131 ± 0.004 **	26.3 ± 0.4% ***	18.4 ± 1.4 **^,§^
**15c**	3	6	0.151 ± 0.013	0.0106 ± 0.0002 **	25.4 ± 0.9% ***	16.8 ± 1.2
**15d**	3	8	0.0260 ± 0.0024 *^,‡^	**0.00624 ± 0.00054** ***^,‡^	25.5 ± 1.1% ***	14.9 ± 1.2
**16a**	1	2	3.50 ± 0.33 ^a^	0.652 ± 0.05 ^a,^*	19.6 ± 1.3% ^a,^***	16.4 ± 1.4 ^a^
**16b**	1	4	0.912 ± 0.016	0.177 ± 0.017	17.2 ± 3.3% **	17.5 ± 1.5 *^,§^
**16c**	1	6	0.205 ± 0.012	0.0488 ± 0.0005 ***	18.7 ± 0.7% ***	15.8 ± 1.4
**16d**	1	8	0.094 ± 0.006 *^,‡^	0.0170 ± 0.0016	17.2 ± 0.2% **	13.9 ± 1.1
**17a**	2	2	2.88 ± 0.19 *	0.464 ± 0.041 *	18.4 ± 1.1% ***	13.4 ± 1.1
**17b**	2	4	0.279 ± 0.022	0.111 ± 0.009	15.8 ± 1.4% ***	15.4 ± 1.3
**17c**	2	6	0.0702 ± 0.0011 *^,‡^	0.0361 ± 0.023	18.5 ± 1.6% ***	16.1 ± 1.1
**17d**	2	8	**0.0151 ± 0.002** *^,‡^	0.00756 ± 0.00042 ***^,‡^	23.6 ± 2.1% ***	13.4 ± 1.2
**18a**	3	2	1.90 ± 0.26 ^a^	0.0838 ± 0.0082 ^a^	26.0 ± 3.9% ^a,^*	13.6 ± 1.2 ^a^
**18b**	3	4	0.436 ± 0.016	0.0678 ± 0.0061	21.4 ± 1.8% ***	15.7 ± 1.2
**18c**	3	6	0.103 ± 0.004 *^,‡^	0.0149 ± 0.0003 ***^,‡^	16.8 ± 0.5% ***	12.5 ± 0.9
**18d**	3	8	0.0308 ± 0.0002 *^,‡^	**0.00596 ± 0.00058** **^,‡^	13.9 ± 1.2% ***	12.3 ± 0.8
Tacrine			0.601 ± 0.047	0.0295 ± 0.0002	n.a.	4.4 ± 0.6 **
BHT			6.0 ± 1.5%	18.9 ± 1.7%	5.6 ± 0.2% ***	n.d.
BNPP			n.a.	n.a.	99.1 ± 0.9% ^b^	n.d.
Donepezil			n.d.	n.d.	n.d.	11.9 ± 0.9

^a^ Data from [43]. ^b^ BNPP IC_50_ CES = 1.80 ± 0.11 µM. Data are presented as mean ± SEM (*n* = 3); m + 4 = the cycloalkyl ring size; n = number of methylene units in the spacer; n.a.—not active; n.d.—not determined. AChE and BChE IC_50_ values, statistically significant differences from tacrine: * *p* < 0.033; **, *p* < 0.002; *** *p* < 0.001; ‡, more potent than tacrine (one-way ANOVA. CES % inhibition, statistically significant differences from BNPP: * *p* < 0.033; ** *p* < 0.002; ***, *p* < 0.001; all compounds were less potent than BNPP. Propidium iodide displacement (PID), statistically significant difference from Donepezil: * *p* < 0.033; ** *p* < 0.002; ***, *p* < 0.001; §, more potent than Donepezil. AChE, BChE and CES: Brown–Forsythe and Welch one-way ANOVA with Dunnett T3 post hoc test; PID: standard one-way ANOVA with Dunnett post hoc test.

**Table 2:** Inhibition constants of cholinesterases by conjugates **15a**,**c**,**d, 18c**,**d** ^a^.

Compound	AChE		eqBChE	
	*K*_i_, nM	α*K*_i_, nM	*K*_i_, nM	α*K*_i_, nM
**15a**	1580 ± 100 ^b,^*	4300 ± 20 ^b,^*	164 ± 5 ^b,^***	437 ± 10 ^b,^***
**15c**	69.8 ± 5.0 *	108 ± 3 *	5.13 ± 0.10 *	9.34 ± 0.62 *
**15d**	19.9 ± 0.8 *	31.4 ± 2.4 *	4.91 ± 0.42 *	5.68 ± 0.06 *
**18c**	83.1 ± 1.4 *	101 ± 5 *	7.83 ± 0.76 **	17.3 ± 0.2 *
**18d**	13.0 ± 1.0 *	25.1 ± 0.2 *	4.25 ± 0.33 *	6.06 ± 0.41 *
**Tacrine**	673.2 ± 50.2	1490.1 ± 89.4	27.4 ± 1.91	73.3 ± 6.61

^a^ Values for *K*_i_ (competitive inhibition constant) and α*K*_i_ (non-competitive inhibition constant) were determined from analyses of slopes of 1/V versus 1/S at various inhibitor concentrations. ^b^ Data from [43]. Values (means ± SEM) are from at least three separate experiments. AChE and eqBChE *K*_i_ and α*K*_i_ statistically significant differences from tacrine: * *p* < 0.033; ** *p* < 0.002; ***, *p* < 0.001 Brown–Forsythe and Welch one-way ANOVA with Dunnett T3 post hoc test. With the exception of compound **15a**, all values for the other compounds were more potent than those for tacrine.

**Table 3:** Inhibition of Aβ_42_ self-aggregation by selected conjugates.

No	m	n	Inhibition of Aβ_42_ Self- Aggregation, % ^a^
**13d**	1	8	49.4 ± 4.3
**14c**	2	6	62.4 ± 4.9 ^b^
**14d**	2	8	70.4 ± 5.6 ^b,c^
**15c**	3	6	43.4 ± 3.9
**15d**	3	8	63.5 ± 5.0 ^b^
**16d**	1	8	54.6 ± 3.9 ^b^
**17c**	2	6	64.1 ± 5.7 ^b^
**17d**	2	8	71.4 ± 4.9 ^b,c^
**18c**	3	6	47.8 ± 3.8
**18d**	3	8	59.6 ± 4.7 ^b^
Tacrine			5.9 ± 0.5
Myricetin			73.2 ± 5.8 ^c^
Propidium iodide			89.3 ± 7.1 ^b^

^a^ Inhibition of Aβ_42_ (50 µM) self-aggregation by the tested compound at 100 µM concentration. m, n—see footnote for Table 1. Values (means ± SEM) are from at least three separate experiments. ^b^ No statistically significant difference from Myricetin (*p* > 0.05); ^c^ No statistically significant difference from Propidium iodide (one-way ANOVA with preselected contrasts between Myricetin and Propidium iodide vs. all other compounds; Šídák post hoc test).

**Table 4:** Primary antioxidant activity of conjugates **13**–**18**.

No	m	n	ABTS^•+^-Scavenging Activity		Ferric-Reducing Antioxidant Power
			TEAC	IC_50_, µM	TE
**13a**	1	2	0.92 ± 0.03 ^a^	22.3 ± 1.5	0.51 ± 0.03 ^a,^***
**13b**	1	4	0.78 ± 0.04	25.4 ± 1.6	0.60 ± 0.02 ***
**13c**	1	6	0.98 ± 0.04	19.4 ± 0.9	0.59 ± 0.01 ***
**13d**	1	8	0.85 ± 0.03	22.7 ± 1.2	0.71 ± 0.03 ***
**14a**	2	2	1.13 ± 0.05 ^a^	17.8 ± 1.5 *	0.58 ± 0.03 ^a,^***
**14b**	2	4	1.00 ± 0.05	19.7 ± 0.9	0.72 ± 0.03 ***
**14c**	2	6	1.10 ± 0.05	18.6 ± 0.9	0.71 ± 0.02 ***
**14d**	2	8	1.06 ± 0.03	18.2 ± 0.7	0.70 ± 0.03 ***
**15a**	3	2	1.11 ± 0.04 ^a^	18.8 ± 0.8	0.52 ± 0.01 ^a,^***
**15b**	3	4	0.89 ± 0.04	22.5 ± 1.4	0.46 ± 0.01 ***
**15c**	3	6	0.90 ± 0.03	22.3 ± 1.1	0.52 ± 0.02 ***
**15d**	3	8	1.00 ± 0.03	19.6 ± 0.8	0.73 ± 0.02 ***
**16a**	1	2	1.39 ± 0.05 ^a,^***	14.6 ± 0.8 ***	0.57 ± 0.02 ***
**16b**	1	4	0.90 ± 0.04	23.6 ± 1.3	0.44 ± 0.02 ***
**16c**	1	6	1.50 ± 0.06 ***	13.4 ± 0.7 ***	0.51 ± 0.02 ***
**16d**	1	8	1.00 ± 0.03	21.3 ± 1.2	0.61 ± 0.01 ***
**17a**	2	2	1.20 ± 0.05 *	16.7 ± 0.8 **	0.52 ± 0.02 ***
**17b**	2	4	1.40 ± 0.06 ***	14.3 ± 0.6 ***	0.46 ± 0.01 ***
**17c**	2	6	1.32 ± 0.08 ***	15.7 ± 0.6 ***	0.44 ± 0.02 ***
**17d**	2	8	1.27 ± 0.05 **	15.2 ± 0.7 ***	0.57 ± 0.01 ***
**18a**	3	2	1.35 ± 0.06 ^a,^***	15.3 ± 0.6 ***	0.44 ± 0.06 ***
**18b**	3	4	1.36 ± 0.06 ***	14.6 ± 0.5 ***	0.38 ± 0.01 ***
**18c**	3	6	1.00 ± 0.03	21.6 ± 1.1	0.43 ± 0.02 ***
**18d**	3	8	1.20 ± 0.05 *	15.8 ± 0.6 ***	0.45 ± 0.01 ***
BHT			0.98 ± 0.03	22.4 ± 1.4	0.96 ± 0.02
Trolox			1.0	20.1 ± 1.2	1.0

^a^ Data from [43]. m, n—see footnote for Table 1. Values (means ± SEM) are from at least three separate experiments. Trolox is used as an internal standard in the antioxidant experiments; as such, it is assigned a value of 1.00 for TEAC and TE. Statistical comparisons (standard one-way ANOVA with the Dunnett post hoc test) are made in reference to BHT as the positive control. TEAC and IC_50_ values show statistically significant differences from BHT: * *p* < 0.033; ** *p* < 0.002; *** *p* < 0.001; these values were all more potent than that of BHT (one-way ANOVA; Dunnett post hoc test). TE values with statistically significant differences from BHT: * *p* < 0.033; ** *p* < 0.002; *** *p* < 0.001; these values were all less potent than that of BHT.

**Table 5:** Antioxidant activity of conjugates in biological system.

No	m	n	Radical Scavenging Capacity, Luminol Chemiluminescence Assay	Inhibition of Spontaneous Lipid Peroxidation in Mouse Brain Homogenate, TBARS Assay
			IC_50_, µM	IC_50_, μM
**14d**	2	8	1.9 ± 0.1 **^,‡^	20.4 ± 2.3 *^,<^
**15c**	3	6	2.3 ± 0.3 **^,‡^	27.6 ± 1.7 **^,<^
**15d**	3	8	3.0 ± 0.1 **^,‡^	29.1 ± 2.4 *^,<^
**17d**	2	8	11.3 ± 1.5 ***^,‡^	17.4 ± 2.1 *^,<^
**18c**	3	6	14.6 ± 0.8 **^,‡^	24.6 ± 2.8 *^,<^
**18d**	3	8	9.2 ± 0.6 **^,‡^	20.2 ± 3.3
Tacrine			n.a.	n.a.
BHT			70.4 ± 4.1	6.9 ± 0.3

m, n—see footnote for Table 1. n.a. = not active. Values (means ±SD) are from at least three separate experiments. Statistically significant differences from BHT: * *p* < 0.033; ** *p* < 0.002; *** *p* < 0.001 Brown–Forsythe and Welch one-way ANOVA with Dunnett T3 post hoc test. ^‡^ More potent than BHT; ^<^ Less potent than BHT.

**Table 6:** Calculated energetic characteristics of BHT, **14b_ts_** and **17b_ts_** in water, kcal/mol.

Compound	BDE^1^	IP	EA	PDE ^1^	PDE ^2^
BHT	75.1	105.3	−12.7	41.0	n.a
**14b_ts_**	83.7	113.9	38.1	26.7	31.4
**17b_ts_**	79.2	114.0	27.3	37.0	32.9

^1^ For the OH bond in the BHT moiety; ^2^ For the NH bond in the spacer.

**Table 7:** Predicted physicochemical and ADMET profiles of compounds **13**–**18**.

Compound	m	n	MW	LogP_ow_	pS_aq_	LogBB	HIA, %	hERG *pK_i_*	hERG *pIC_50_*	QED
**13a**	1	2	443.63	5.82	6.82	0.24	100	6.22	6.17	0.35
**13b**	1	4	471.69	6.35	7.39	0.48	100	6.18	6.55	0.28
**13c**	1	6	499.74	6.99	7.96	0.40	100	6.09	6.95	0.23
**13d**	1	8	527.79	7.38	8.24	0.77	100	6.45	7.39	0.19
**14a**	2	2	457.66	6.14	7.19	0.27	100	6.27	6.06	0.32
**14b**	2	4	485.71	6.64	7.65	0.51	100	6.22	6.43	0.27
**14c**	2	6	513.77	7.31	8.10	0.43	100	6.13	6.82	0.22
**14d**	2	8	541.82	7.55	8.56	0.79	100	6.49	7.26	0.19
**15a**	3	2	471.69	6.40	7.44	0.30	100	6.27	6.17	0.23
**15b**	3	4	499.74	6.98	7.96	0.54	100	6.22	6.55	0.20
**15c**	3	6	527.79	7.36	8.32	0.46	100	6.13	6.95	0.17
**15d**	3	8	555.85	7.70	8.72	0.82	100	6.49	7.39	0.14
**16a**	1	2	445.65	5.33	5.68	0.22	100	5.87	5.83	0.39
**16b**	1	4	473.70	5.89	6.23	0.49	100	6.43	6.29	0.31
**16c**	1	6	501.76	6.42	6.80	0.48	100	6.23	6.43	0.25
**16d**	1	8	529.81	6.88	7.30	0.84	100	6.60	6.81	0.21
**17a**	2	2	459.68	5.65	5.96	0.25	100	5.92	5.74	0.36
**17b**	2	4	487.73	6.18	6.51	0.52	100	6.47	6.19	0.29
**17c**	2	6	515.78	6.67	7.06	0.51	100	6.27	6.33	0.24
**17d**	2	8	543.84	7.11	7.50	0.87	100	6.64	6.71	0.20
**18a**	3	2	473.70	5.92	6.28	0.28	100	5.93	5.82	0.27
**18b**	3	4	501.76	6.40	6.81	0.55	100	6.47	6.28	0.22
**18c**	3	6	529.81	6.85	7.28	0.54	100	6.28	6.43	0.18
**18d**	3	8	557.86	7.39	7.68	0.90	100	6.64	6.82	0.15

MW—molecular weight, LogP_ow_—octanol–water partition coefficient, pS_aq_—aqueous solubility [−log(M)], LogBB—blood–brain barrier distribution, HIA—human intestinal absorption [%], hERG *pKi*—hERG potassium channel affinity [−log(M)], hERG *pIC_50_*—hERG potassium channel inhibitory activity [−log(M)], abd QED—quantitative estimate of drug-likeness.

## Data Availability

Data are contained within the article and supplementary materials.

## Supplementary Materials

The following supporting information can be downloaded at: https://www.mdpi.com/article/10.3390/molecules29020321/s1: Figures S1–S33: ^1^H and ^13^C spectra of compounds **13b**–**d**, **14b**–**d**, **15b**–**d**, **16b**–**d**, **17a**–**d**, **18b**–**d**; Quantum–Chemical Calculations of AOA.
