# Supplementary material for: Combining Experimental and Computational Methods to Produce Conjugates of Anticholinesterase and Antioxidant Pharmacophores with Linker Chemistries Affecting Biological Activities Related to Treatment of Alzheimer’s Disease
Source: Molecules. 2024 Jan 9;29(2):321. doi: 10.3390/molecules29020321 (PMC10820264; doi:10.3390/molecules29020321)
Supplement: Supplementary file 1 [file molecules-29-00321-s001.zip › molecules-2724156-supplementary.pdf]

Table of Contents

NMR spectra of synthesized compounds 3

Figure S1. ^{1}H spectrum of compound **13b**....3

Figure S2. ^{1}H NMR spectrum of compound **13c**....4

Figure S3. ^{13}C NMR spectrum of compound **13c**....5

Figure S4. ^{1}H spectrum of compound **13d**....6

Figure S5. ^{13}C NMR spectrum of compound **13d**....7

Figure S6. ^{1}H NMR spectrum of compound **14b**....8

Figure S7. ^{13}C NMR spectrum of compound **14b**....9

Figure S8. ^{1}H NMR spectrum of compound **14c**....10

Figure S9. ^{1}H NMR spectrum of compound **14d**....11

Figure S10. ^{13}C NMR spectrum of compound **14d**....12

Figure S12. ^{13}C NMR spectrum of compound **15b**....14

Figure S13. ^{1}H NMR spectrum of compound **15c**....15

Figure S14. ^{13}C NMR spectrum of compound **15c**....16

Figure S15. ^{1}H NMR spectrum of compound **15d**....17

Figure S16. ^{13}C NMR spectrum of compound **15d**....18

Figure S17. ^{1}H NMR spectrum of compound **16b**....19

Figure S18. ^{1}H NMR spectrum of compound **16c**....20

Figure S19. ^{13}C NMR spectrum of compound **16c**....21

Figure S20. ^{1}H NMR spectrum of compound **16d**....22

Figure S21. ^{1}H NMR spectrum of compound **17a**....23

Figure S22. ^{13}C NMR spectrum of compound **17a**....24

Figure S23. ^{1}H NMR spectrum of compound **17b**....25

Figure S24. ^{13}C NMR spectrum of compound **17b**....26

Figure S25. ^{1}H NMR spectrum of compound **17c**....27

Figure S26. ^{1}H NMR spectrum of compound **17d**....28

Figure S27. ^{13}C NMR spectrum of compound **17d**....29

Figure S28. ^{1}H NMR spectrum of compound **18b**....30

Figure S29. ^{13}C NMR spectrum of compound **18b**....31

Figure S30. ^{1}H NMR spectrum of compound **18c**....32

Figure S31. ^{13}C NMR spectrum of compound **18c**....33

Figure S32. ^{1}H NMR spectrum of compound **18d**....34

Figure S33. ^{13}C NMR spectrum of compound **18d**....35

Quantum-Chemical Calculations of AOA....36

Estimation of protonation state....36

Table S1. p*K*a values according to Marvin prediction....36

Table S2. PA in water, kcal/mol.....36

Table S3. AOA characteristics in water, kcal/mol.....37

Table S4. AOA characteristics in ethanol, kcal/mol.....37

Luminol chemiluminescence 38

Table S5. Change in free energy, $\Delta G^{(i)}$, for reactions (S1)-(S7) in kcal/mol. 39

Figure S34. Change in free energy, ΔG as a result of reaction (S1) - (S7) for **BHT**, **$14b_t$**, **$14b_{ts}$**, and **$17b_{ts}$** compounds. 39

Inhibition of spontaneous lipid peroxidation 40

Figure S35. Reaction pathway for quenching the $HO^{\bullet}$ radical via the H-atom transfer from the antioxidant molecule. 40

Table S6. Inhibition of spontaneous lipid peroxidation in mice brain homogenate (TBARS Assay), BDE and reactant energies at all stages of reaction (S8). 40

ABTS and FRAP tests 41

Figure S36. Visualization of frontier molecular orbitals: antioxidants HOMO. 41

References 41

NMR spectra of synthesized compounds

13b

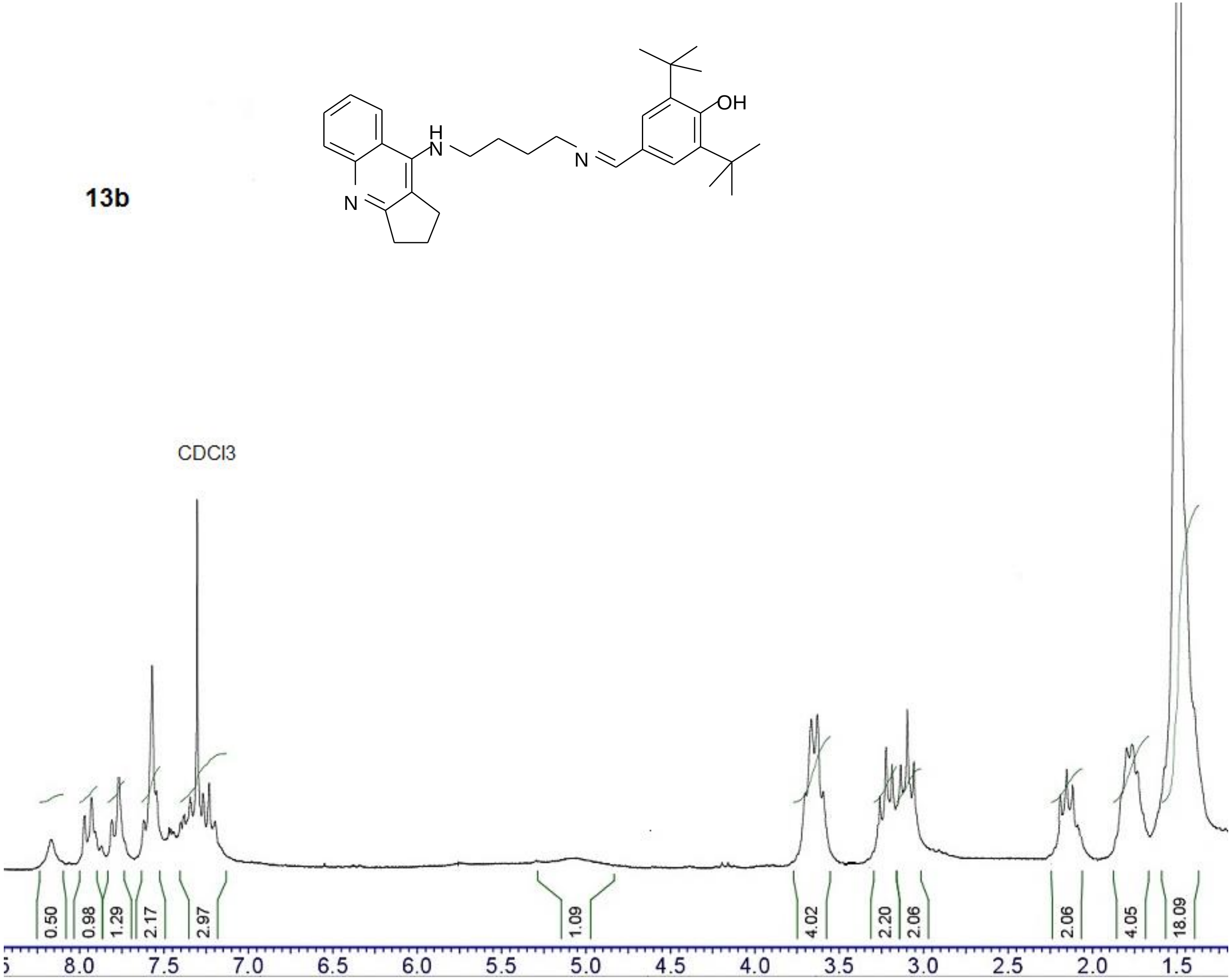

Figure S1. ^{1}H spectrum of compound **13b**

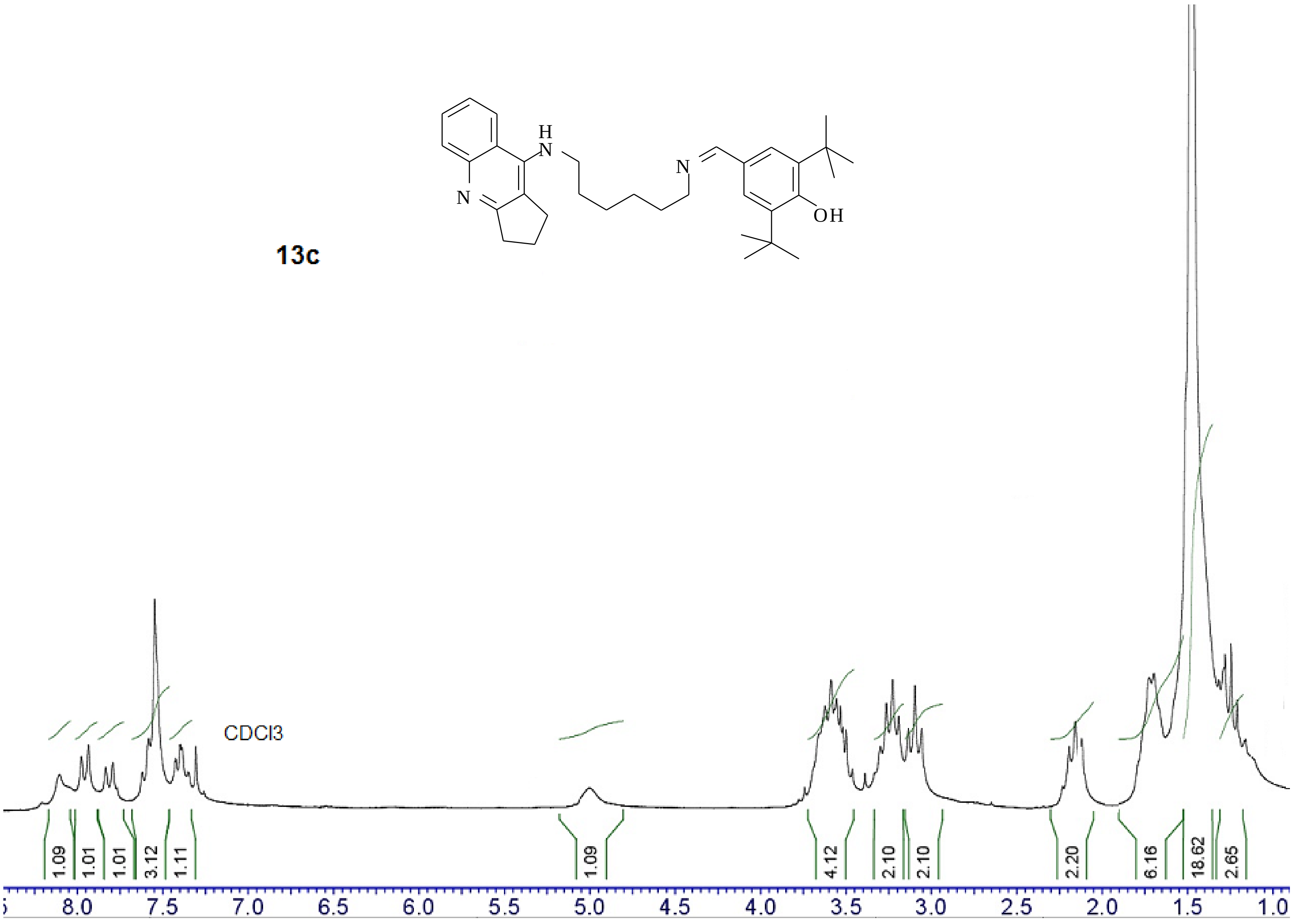

Figure S2. ^{1}H NMR spectrum of compound **13c**

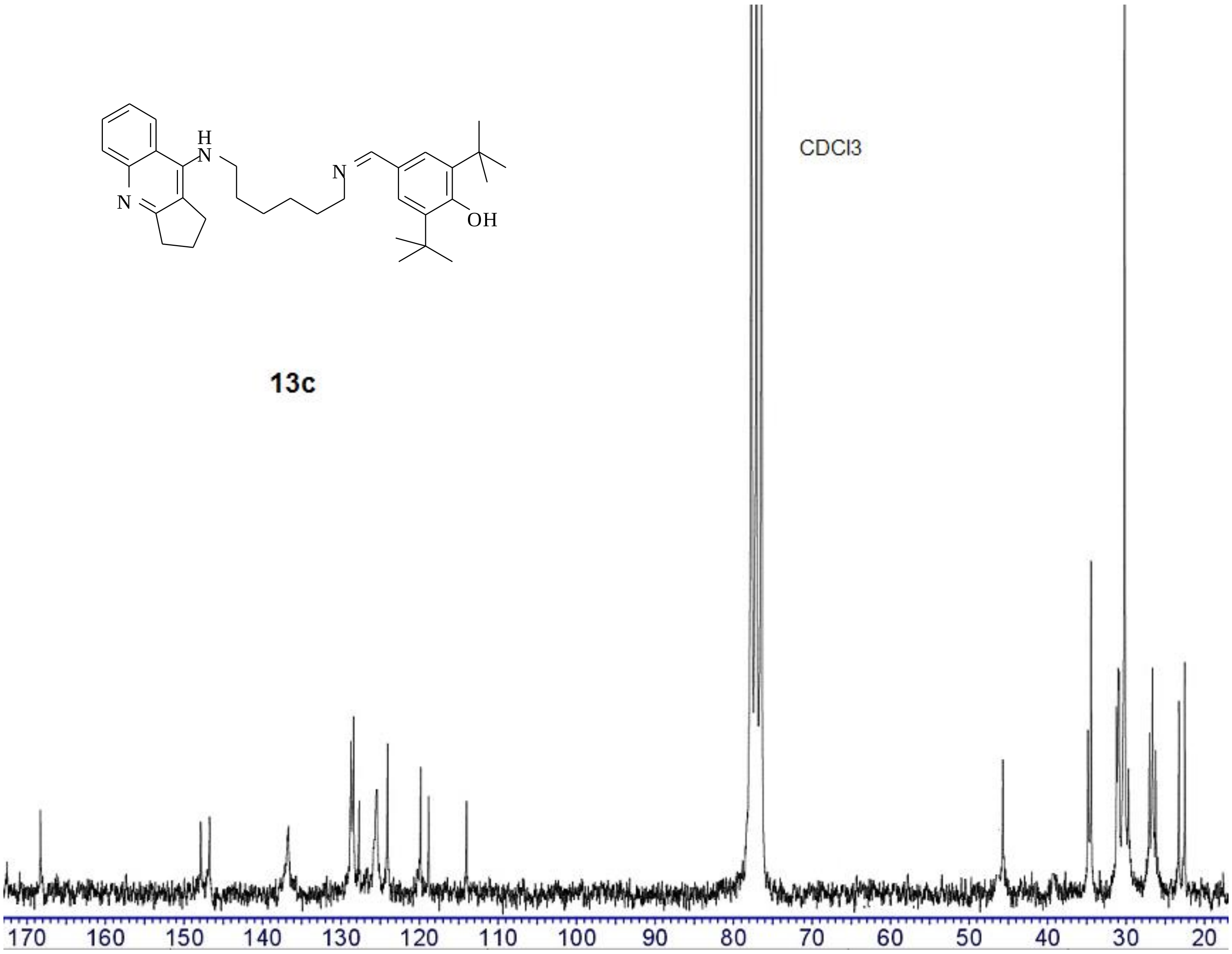

Figure S3. ^{13}C NMR spectrum of compound **13c**

13d

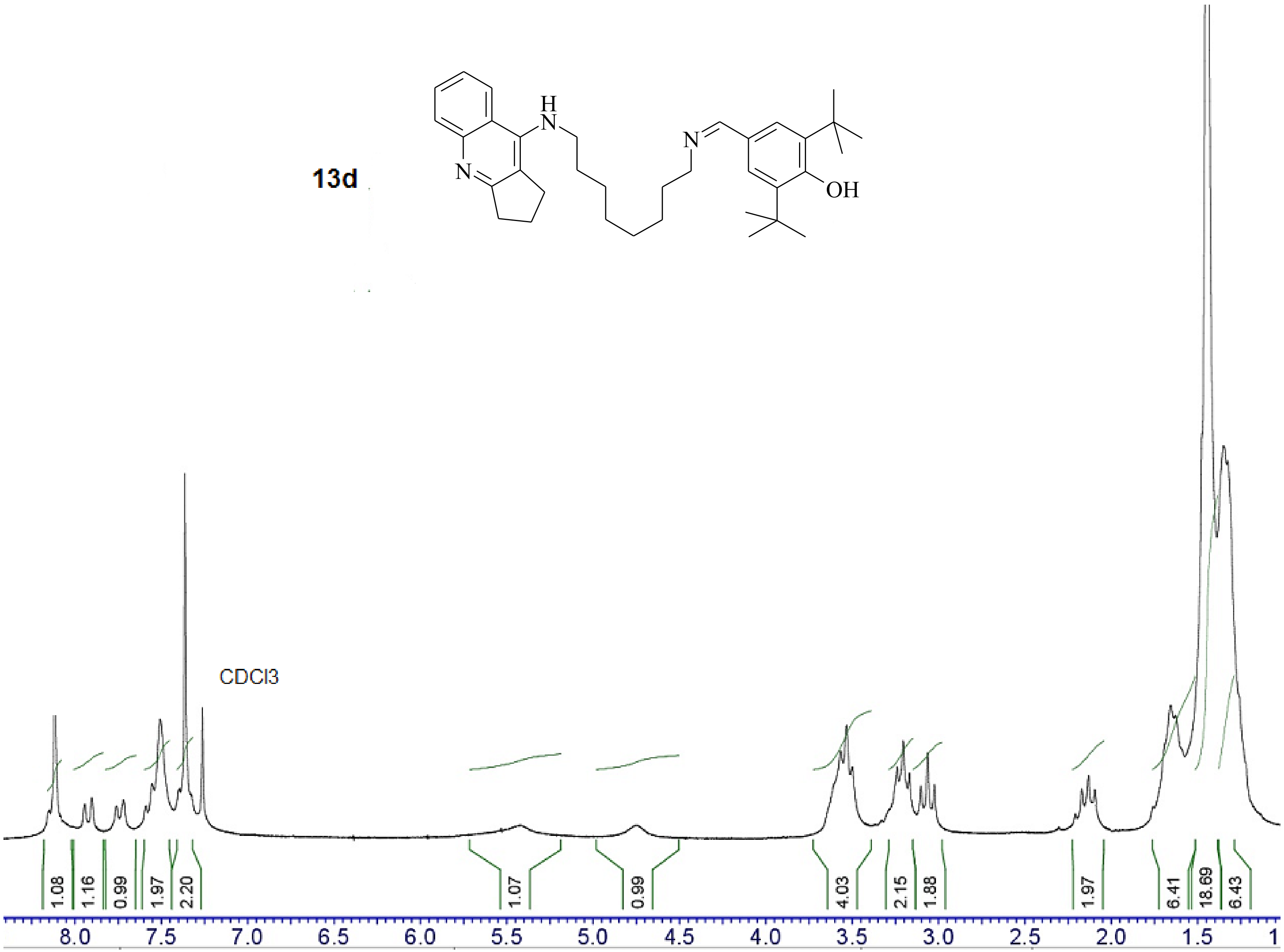

Figure S4. ¹H spectrum of compound **13d**

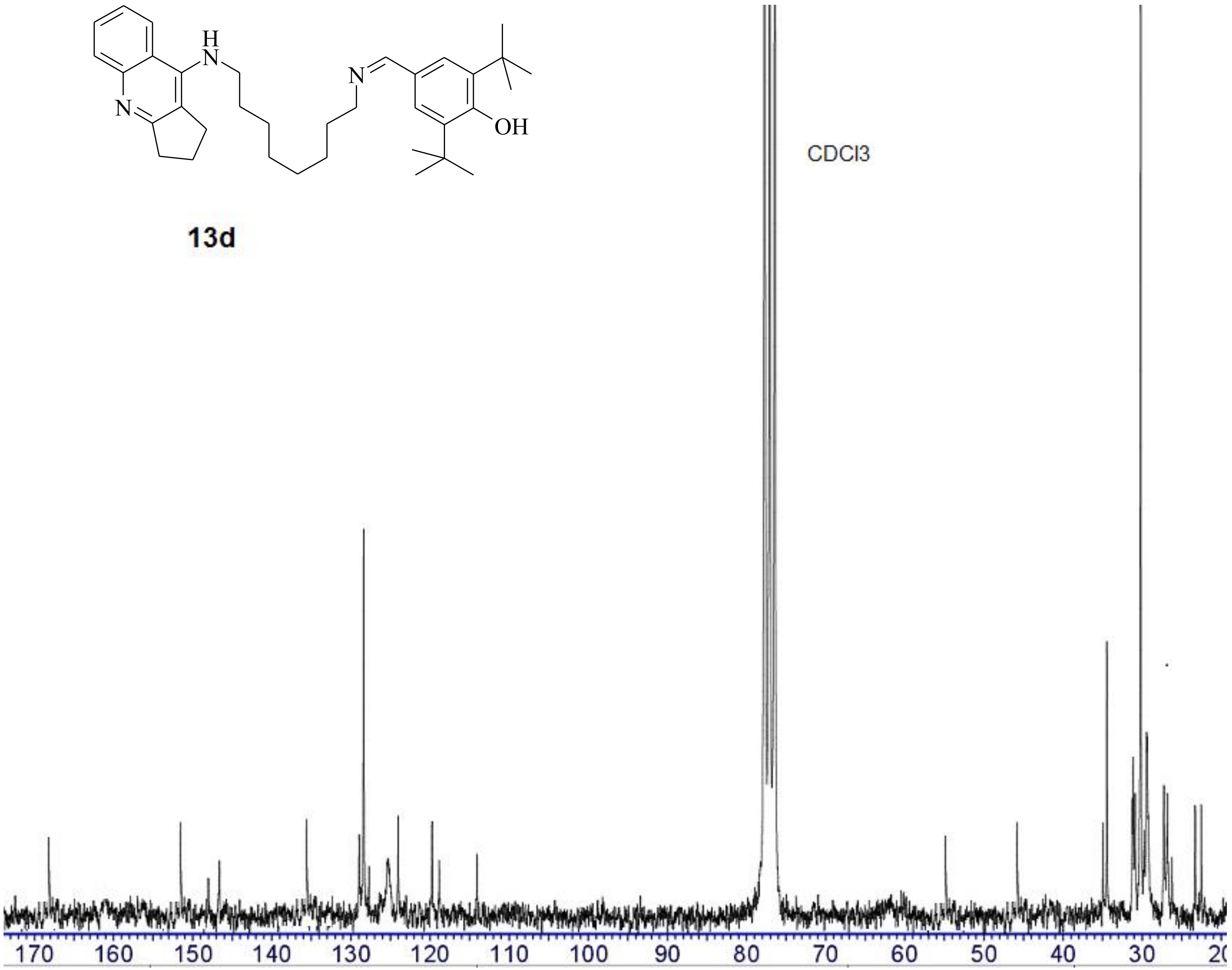

Figure S5. ^{13}C NMR spectrum of compound **13d**

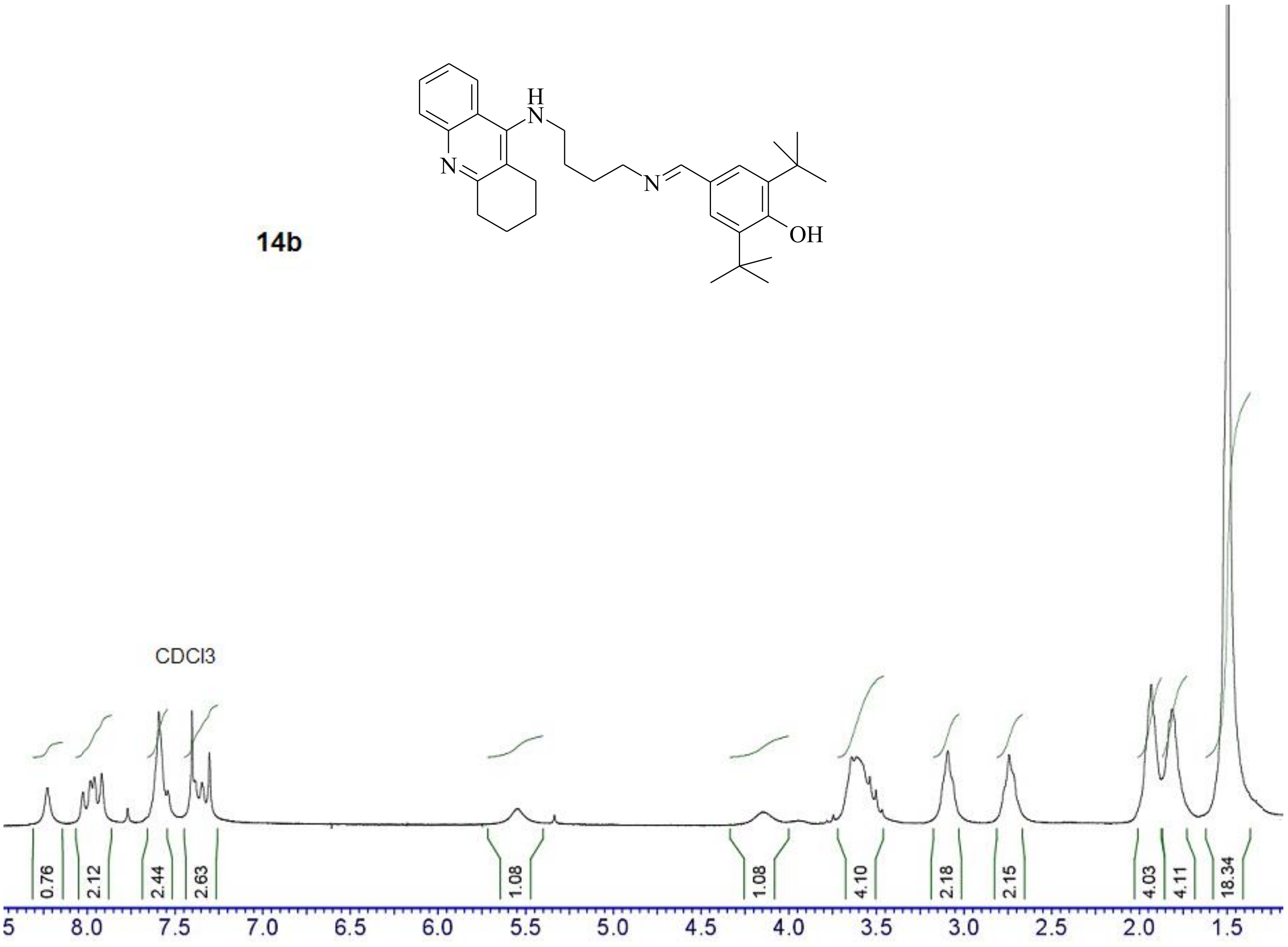

Figure S6. ^{1}H NMR spectrum of compound **14b**

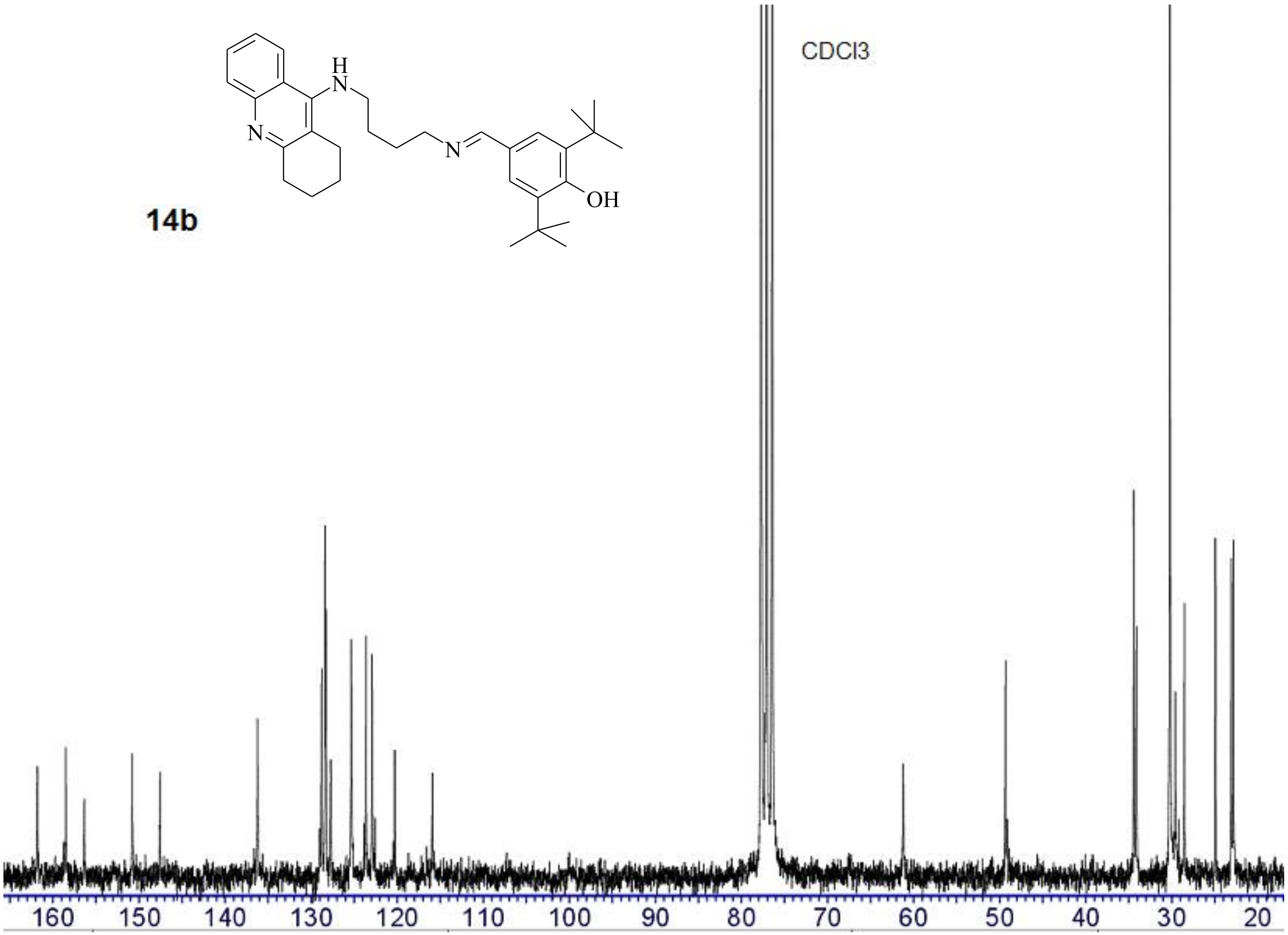

Figure S7. ^{13}C NMR spectrum of compound **14b**

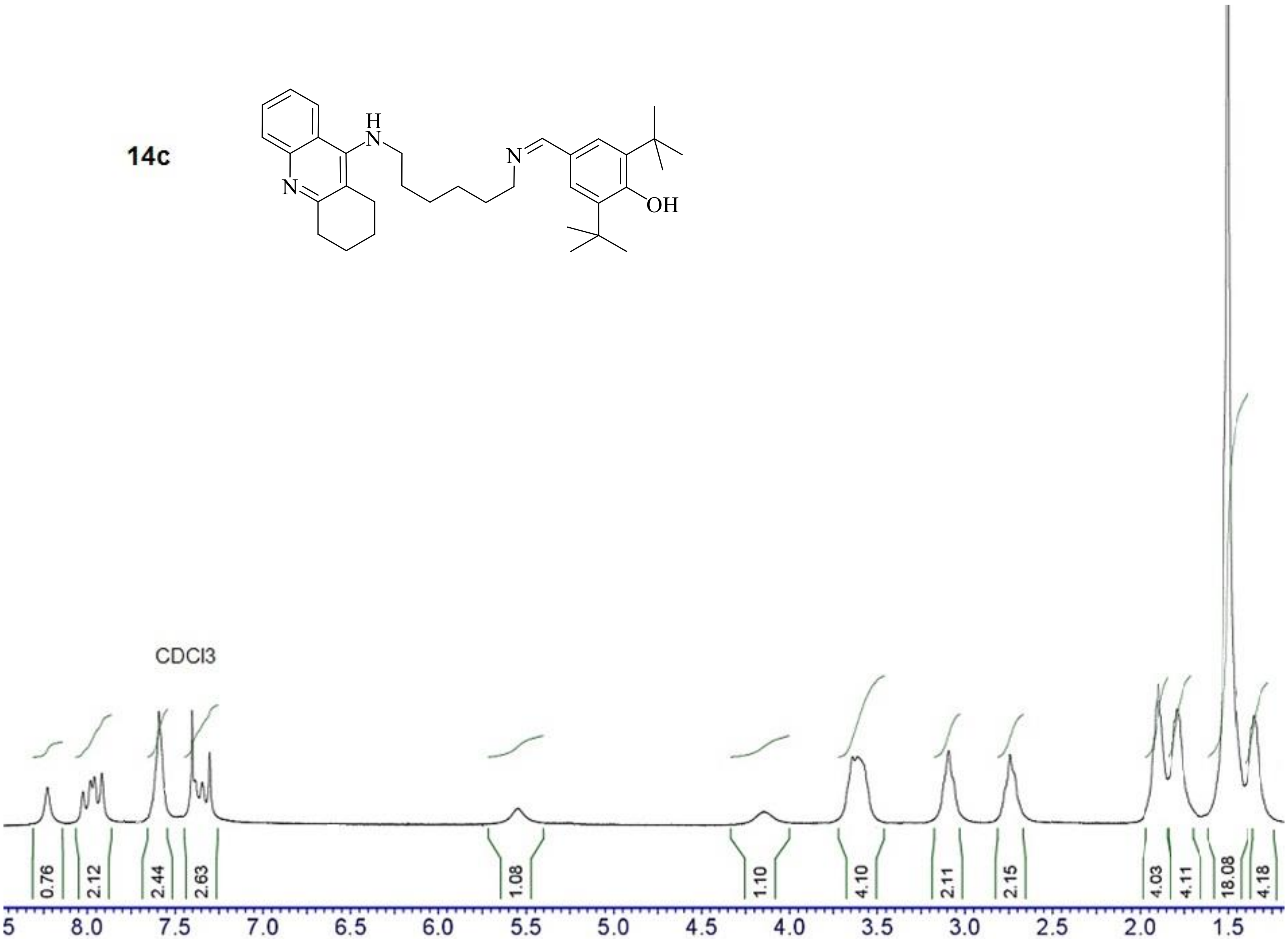

Figure S8. ^{1}H NMR spectrum of compound **14c**

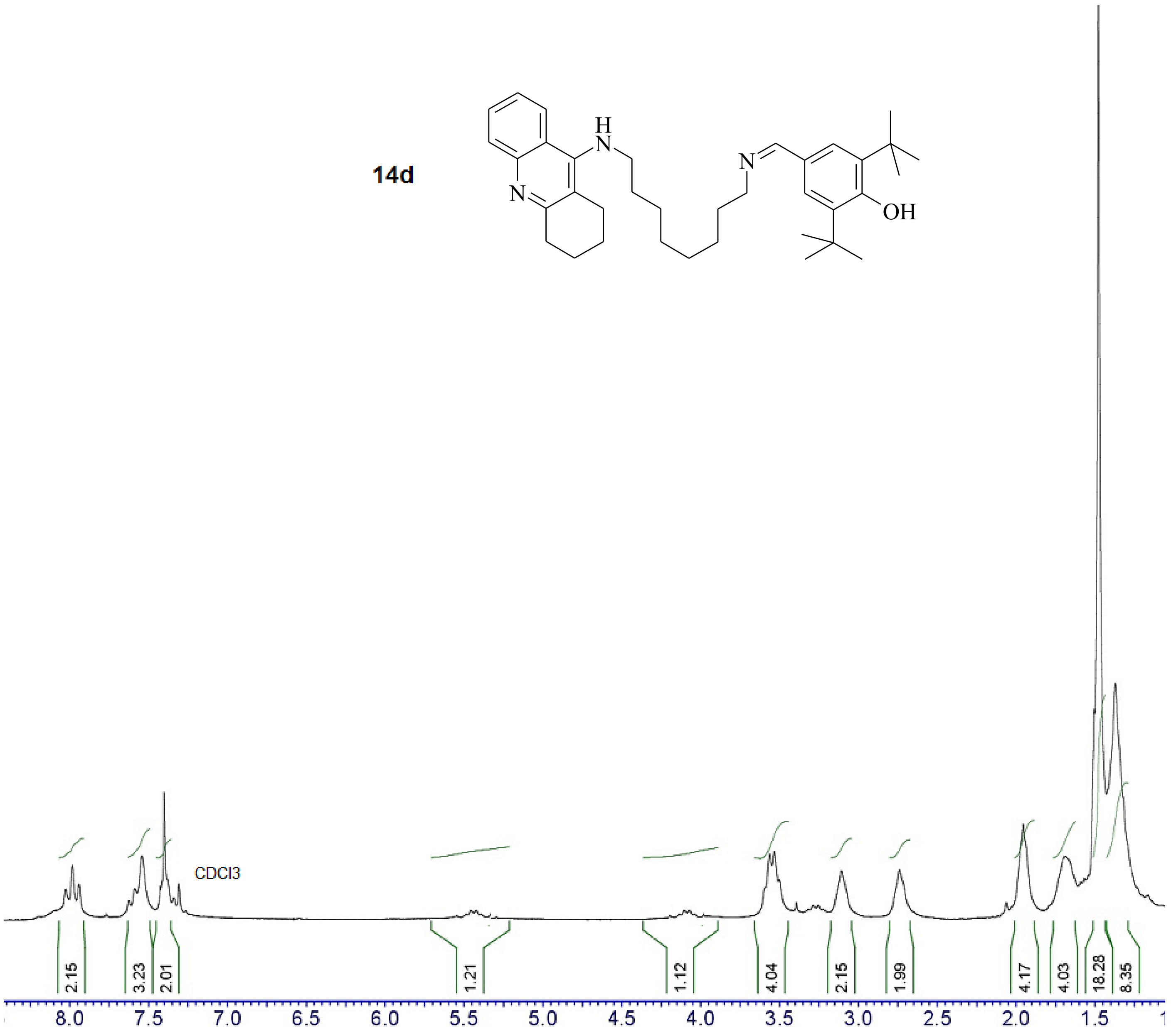

Figure S9. ^{1}H NMR spectrum of compound **14d**

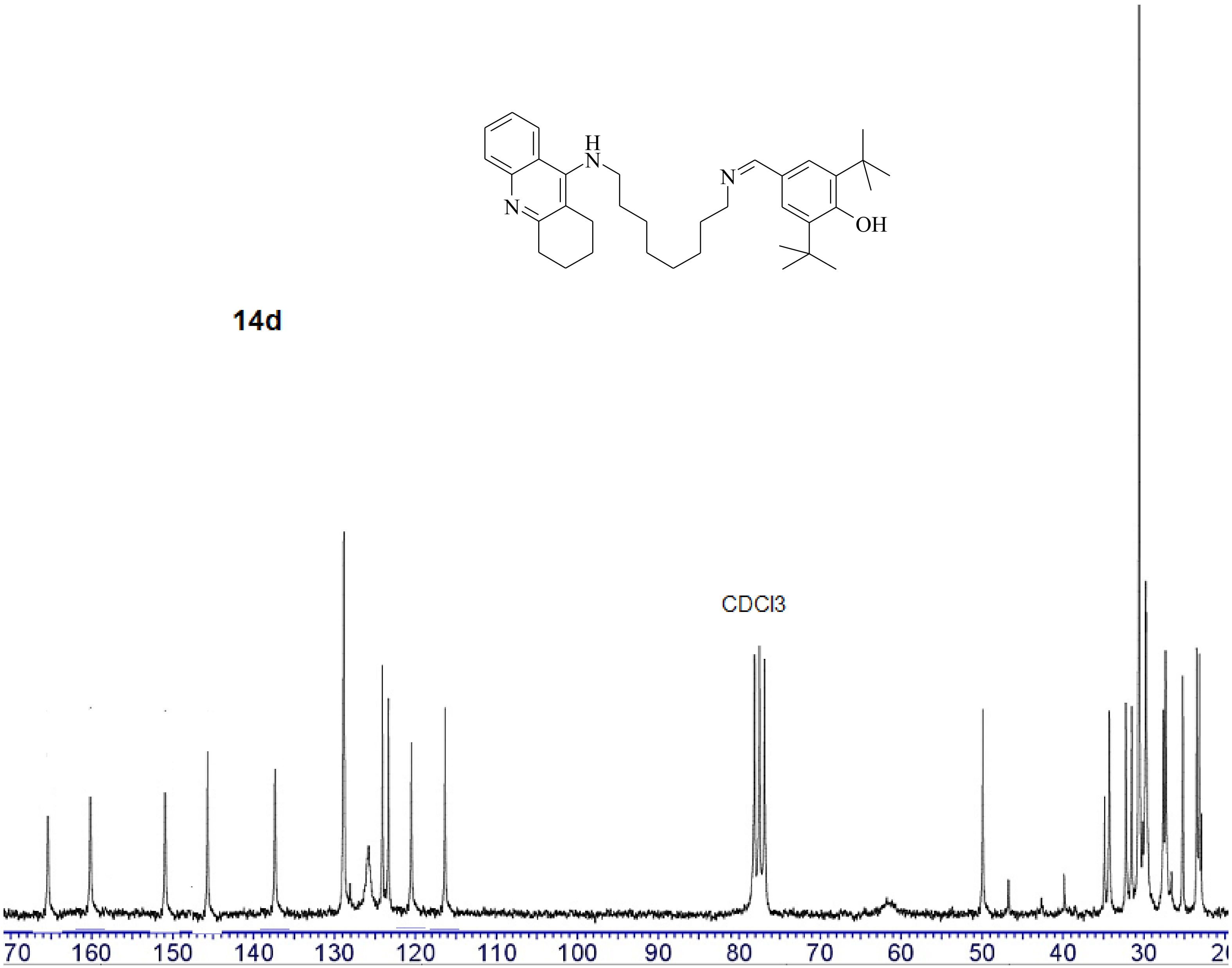

Figure S10. ^{13}C NMR spectrum of compound **14d**

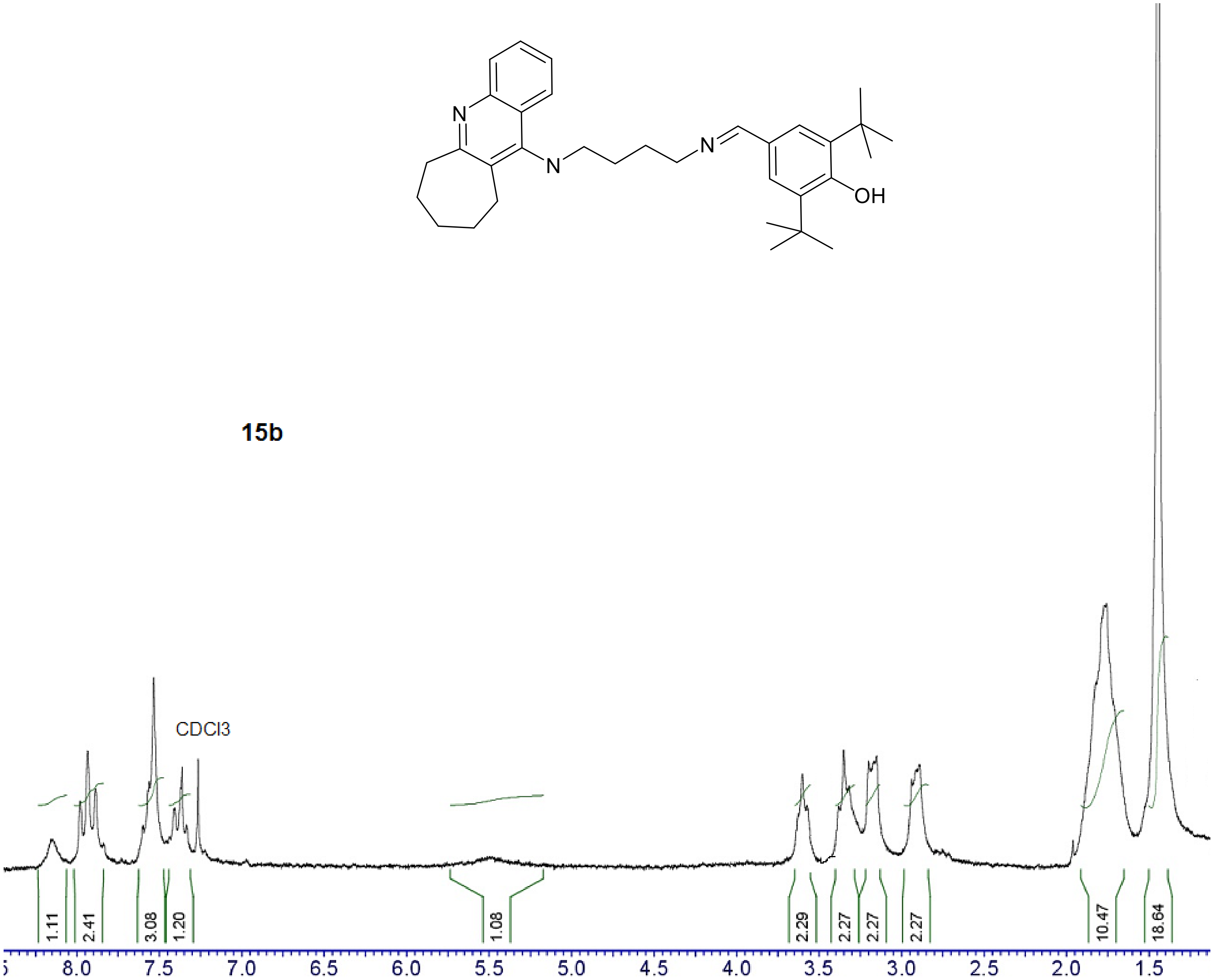

Figure S11. ^{1}H NMR spectrum of compound **15b**

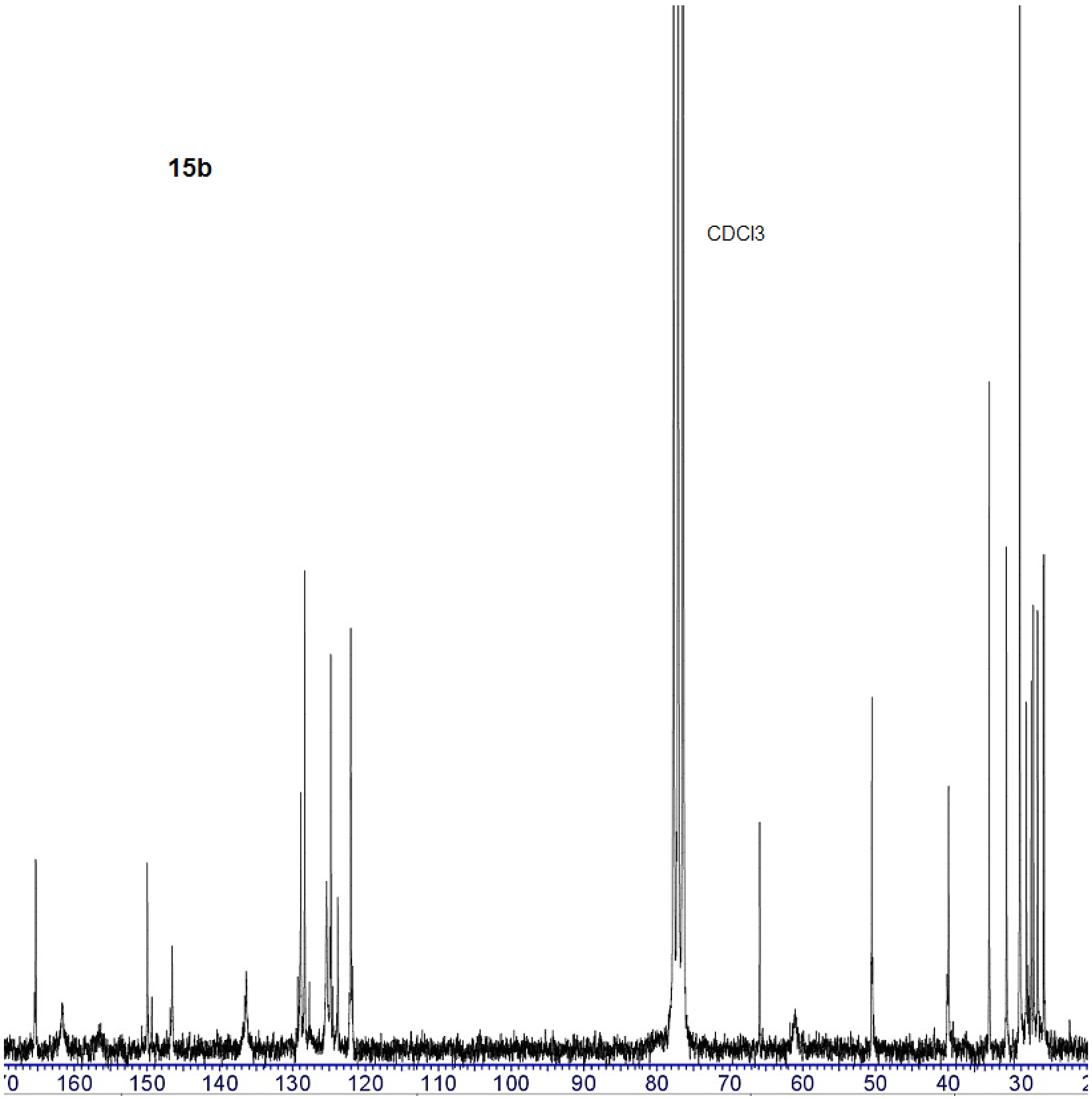

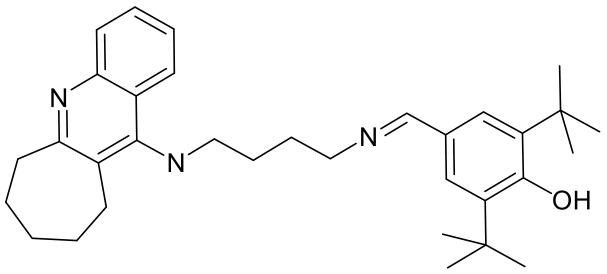

Figure S12. ^{13}C NMR spectrum of compound **15b**

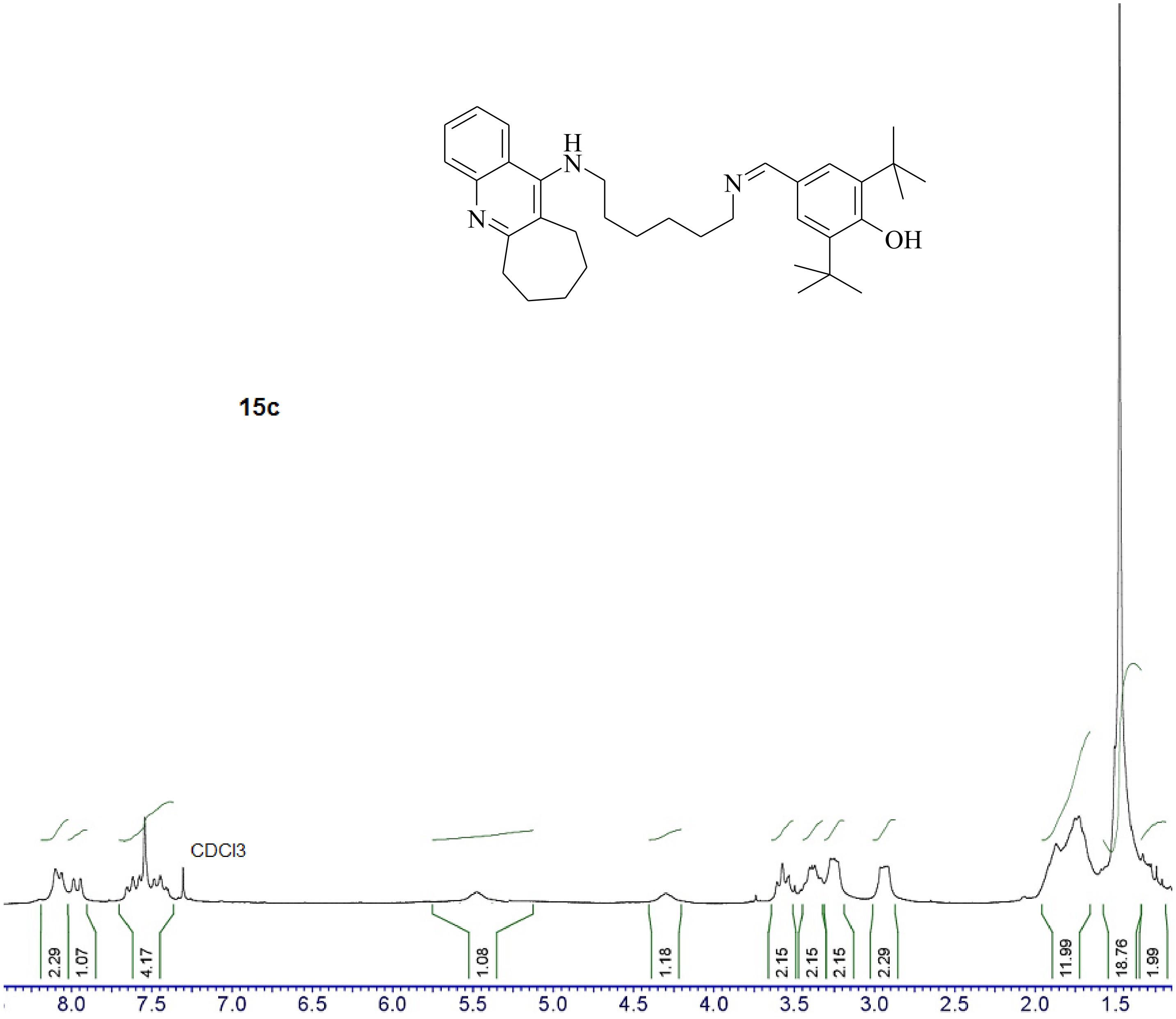

Figure S13. ^{1}H NMR spectrum of compound **15c**

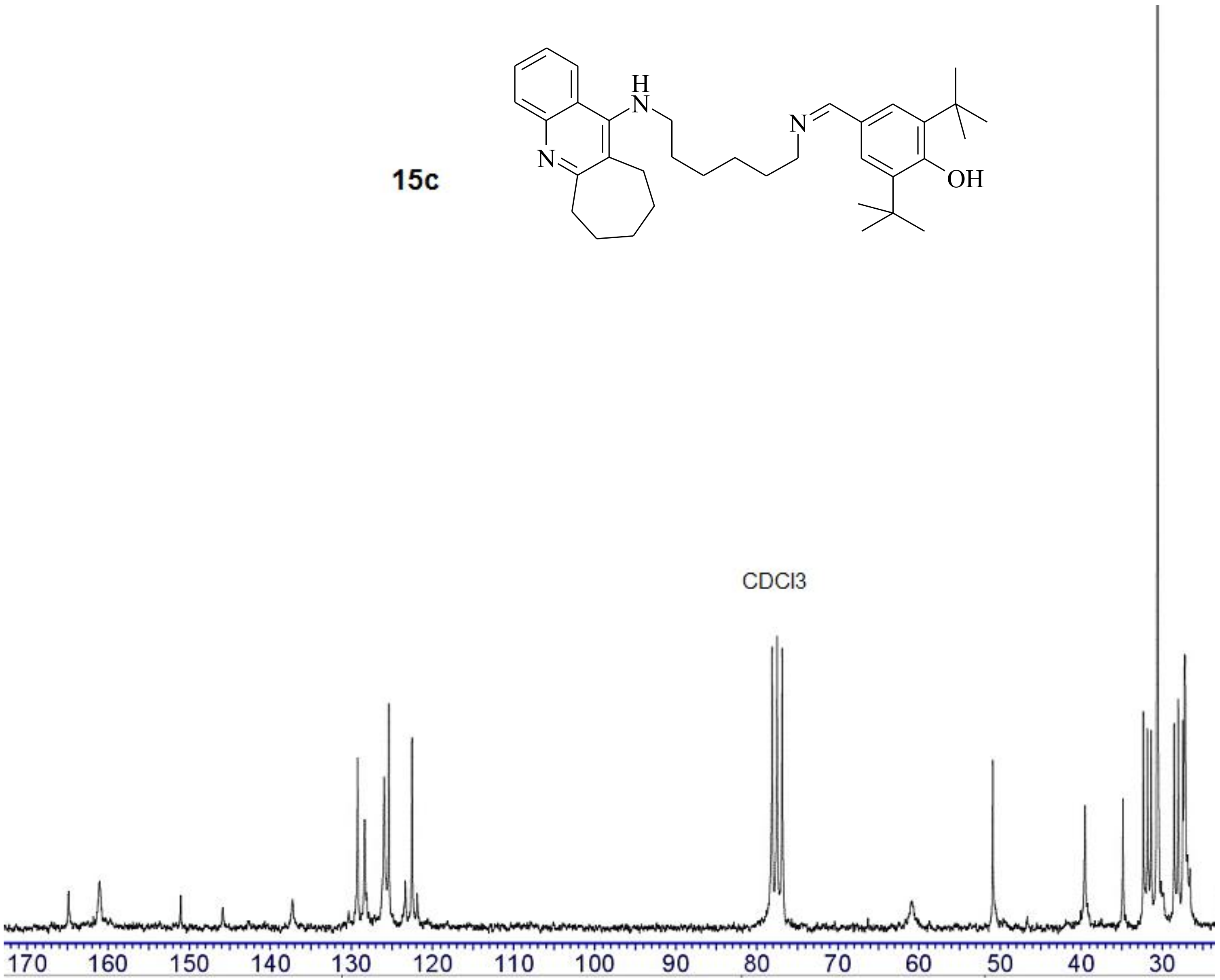

Figure S14. ^{13}C NMR spectrum of compound **15c**

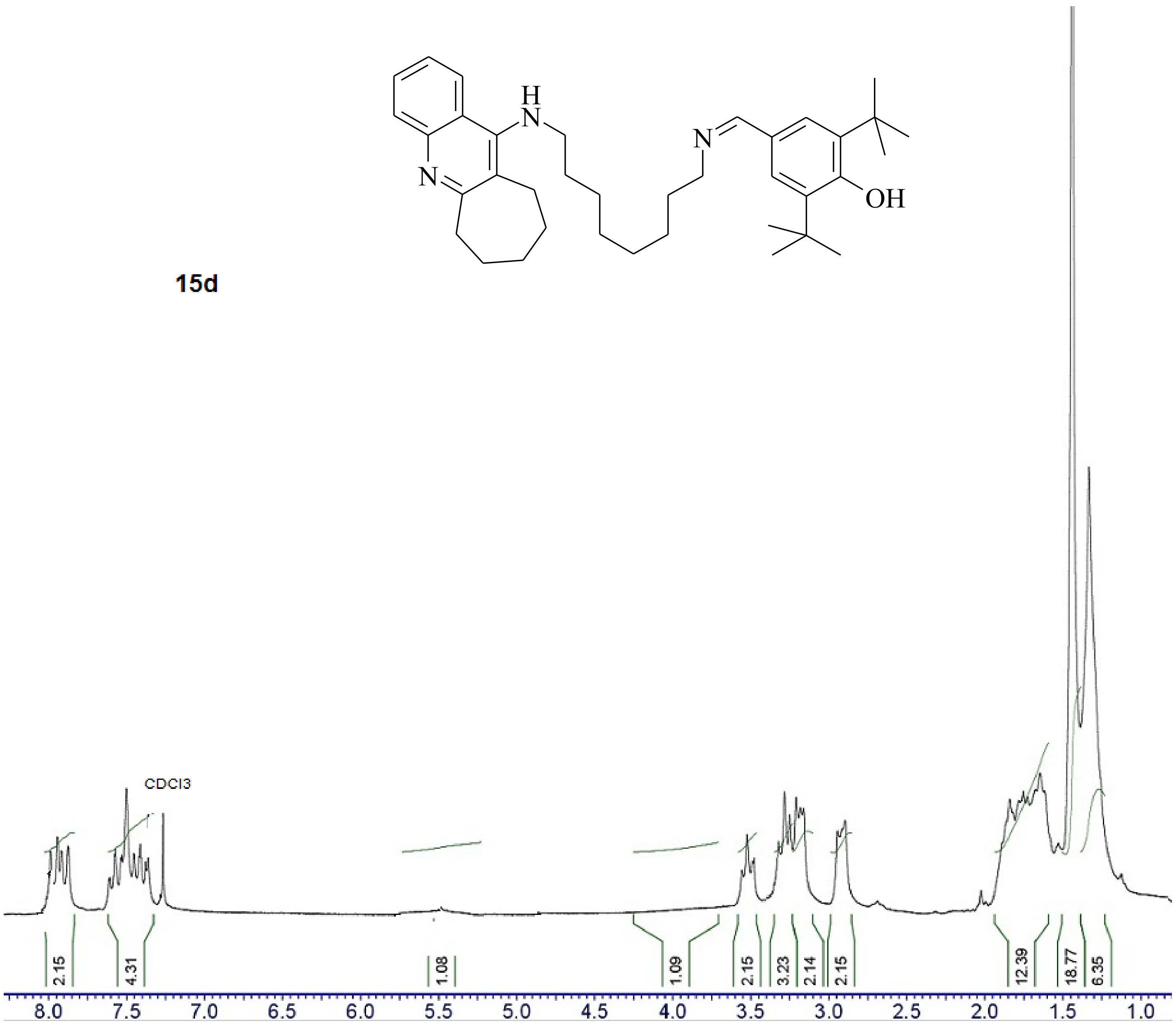

Figure S15. ^{1}H NMR spectrum of compound **15d**

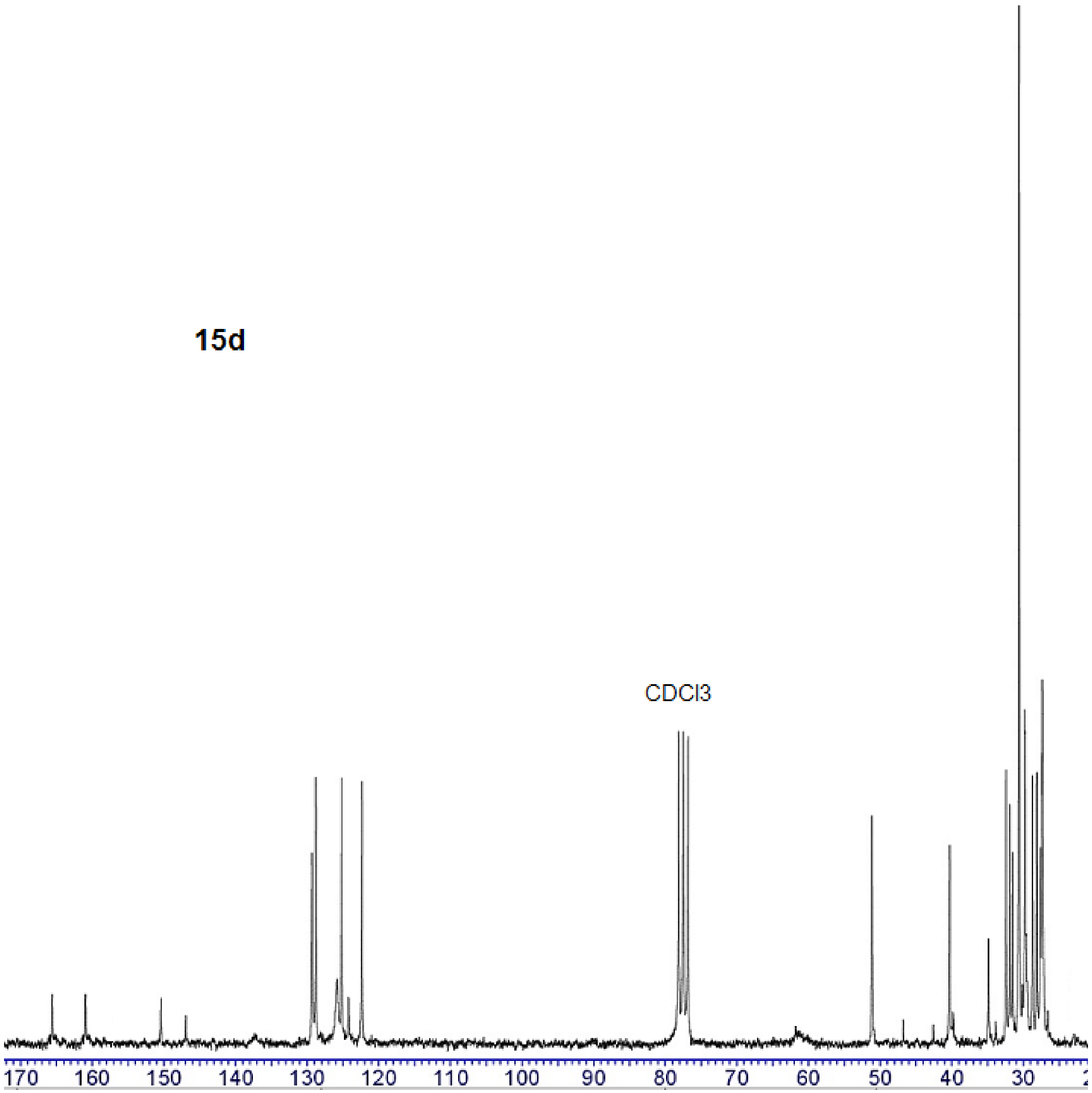

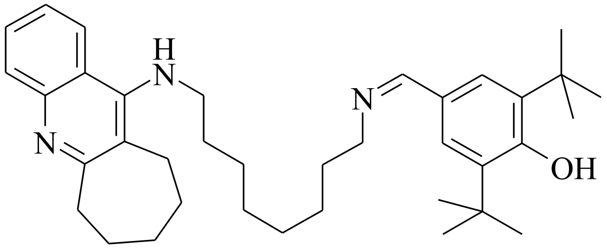

Figure S16. ^{13}C NMR spectrum of compound **15d**

16b

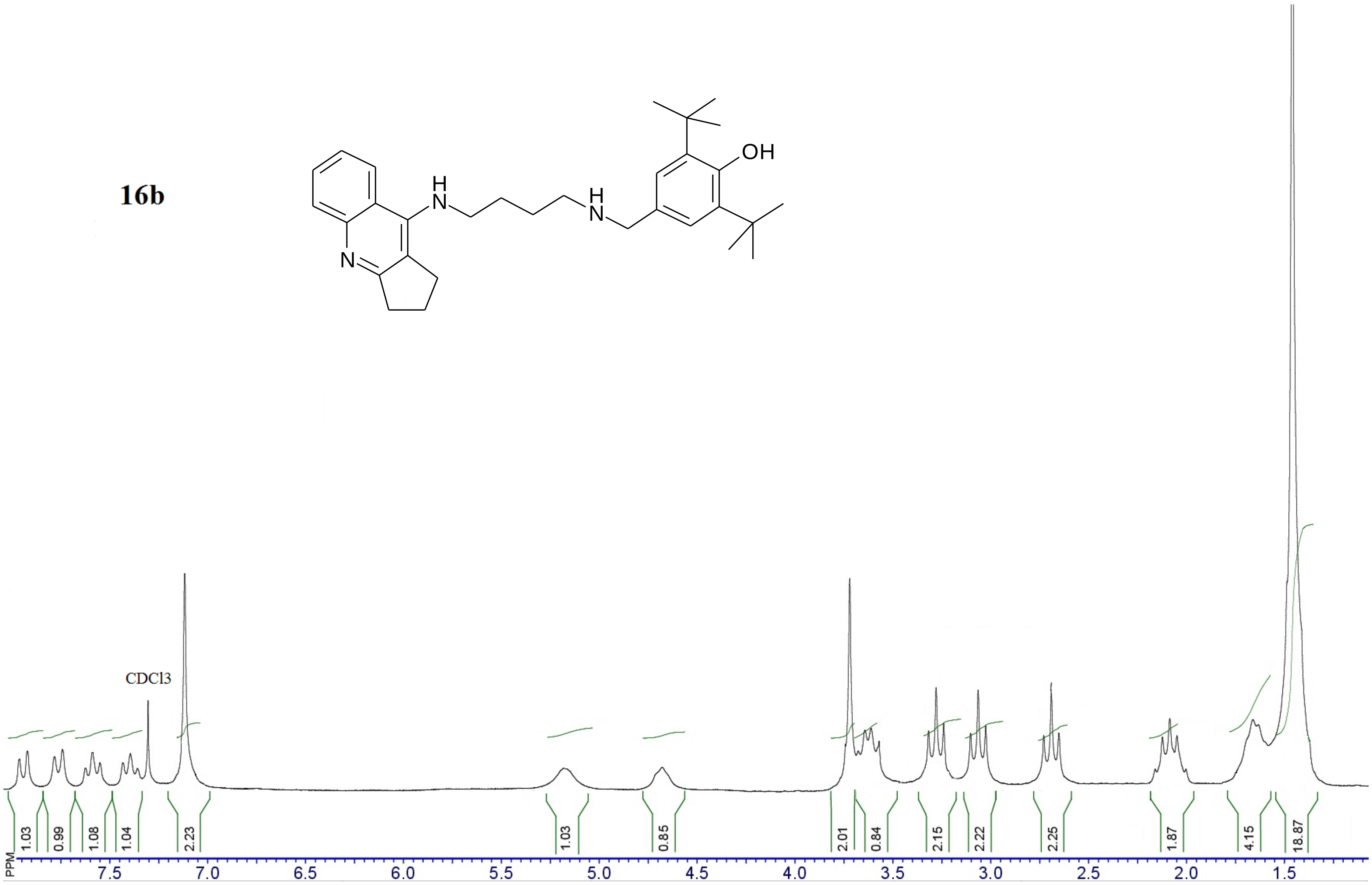

Figure S17. ^{1}H NMR spectrum of compound **16b**

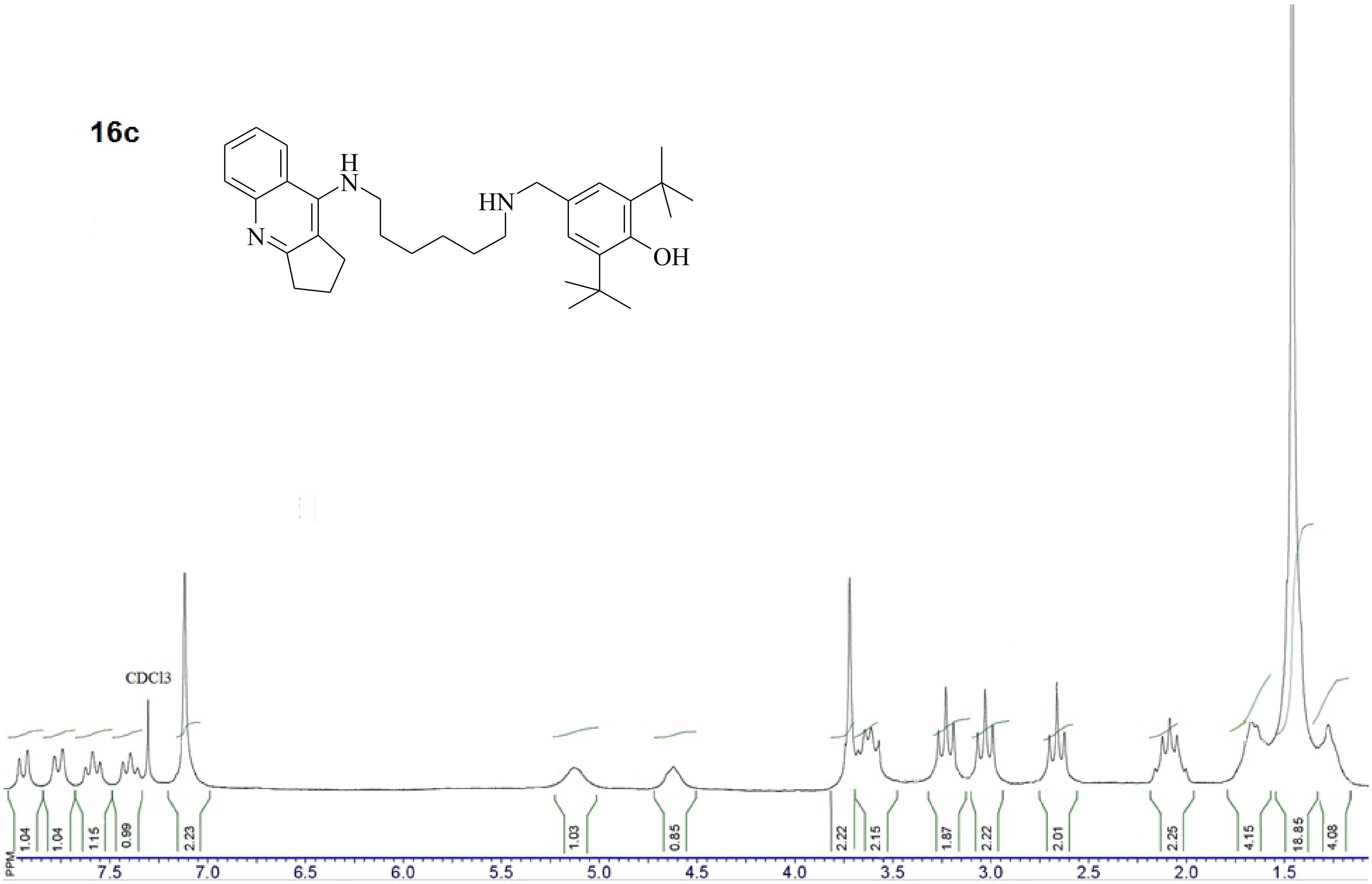

Figure S18. ^{1}H NMR spectrum of compound **16c**

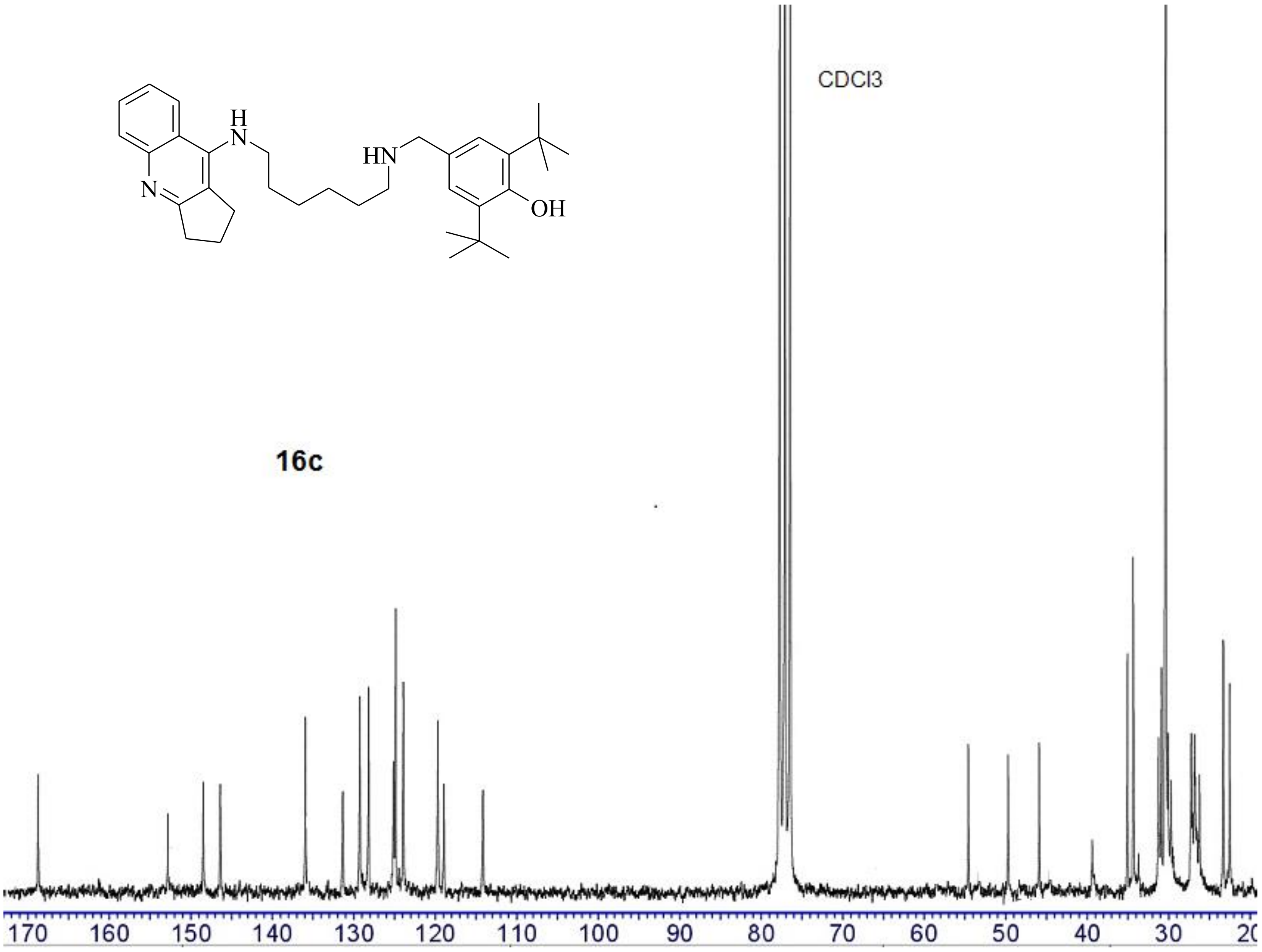

Figure S19. ^{13}C NMR spectrum of compound **16c**

16d

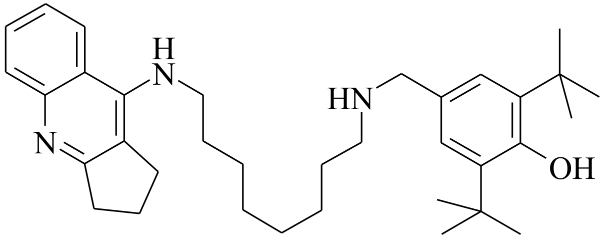

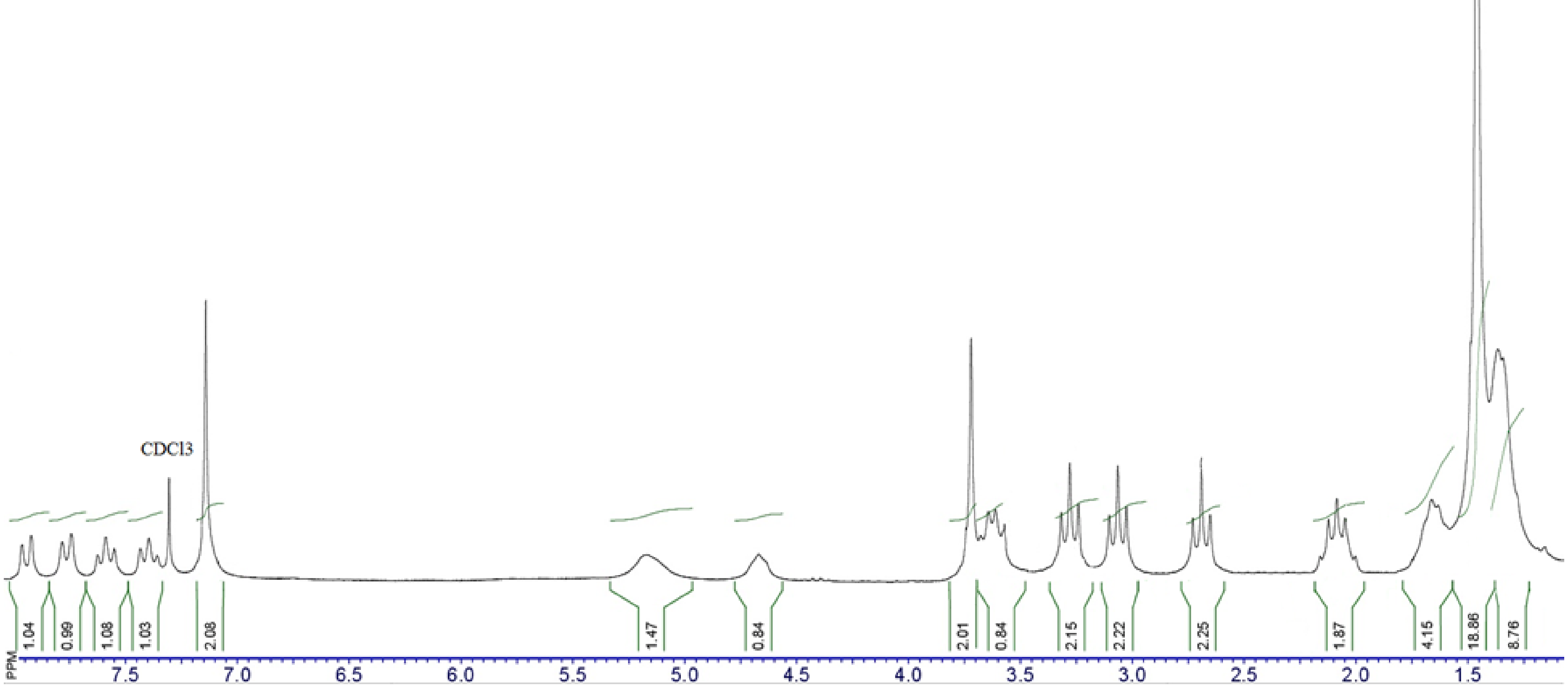

Figure S20. ^{1}H NMR spectrum of compound **16d**

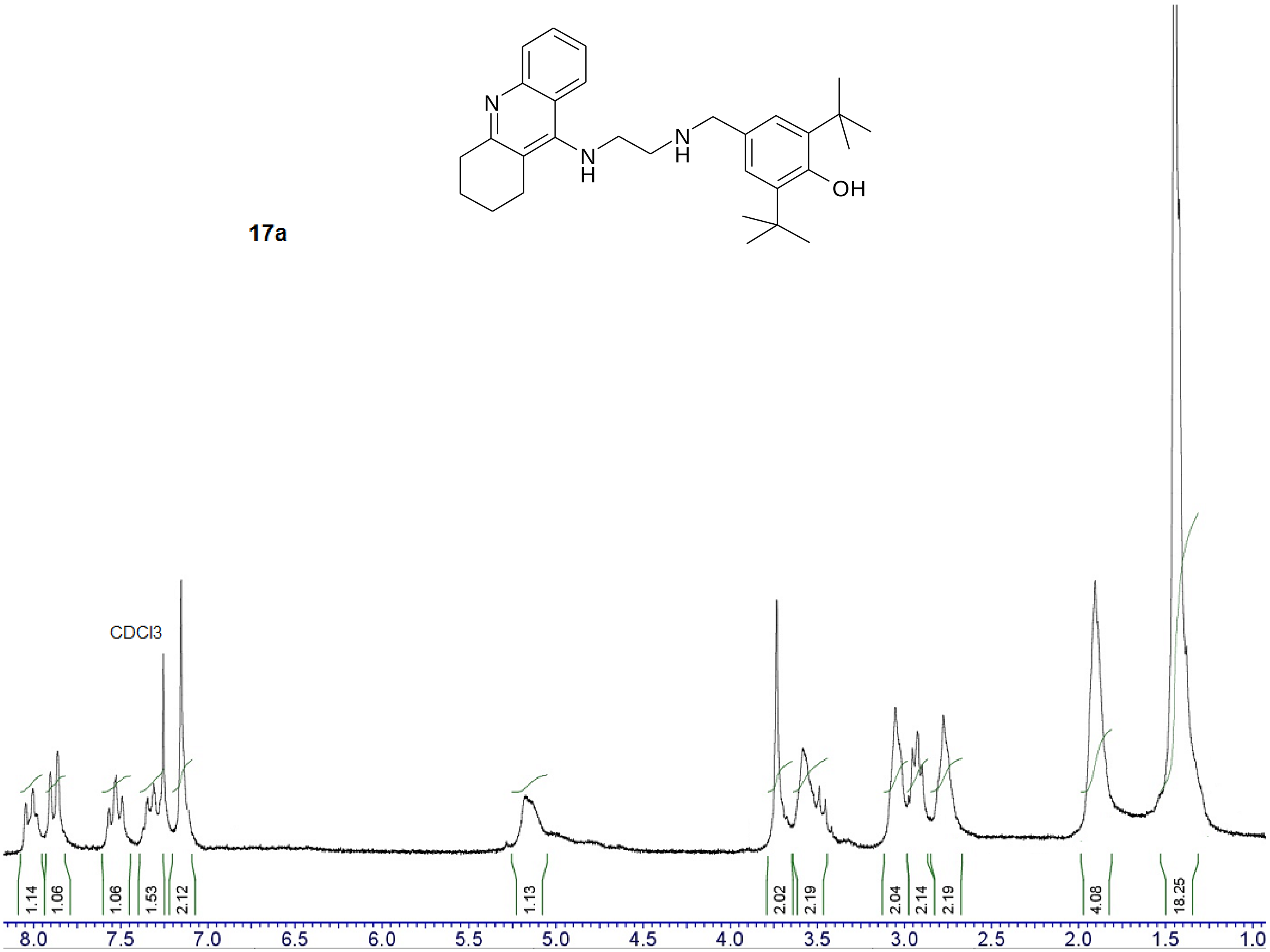

Figure S21. ^{1}H NMR spectrum of compound **17a**

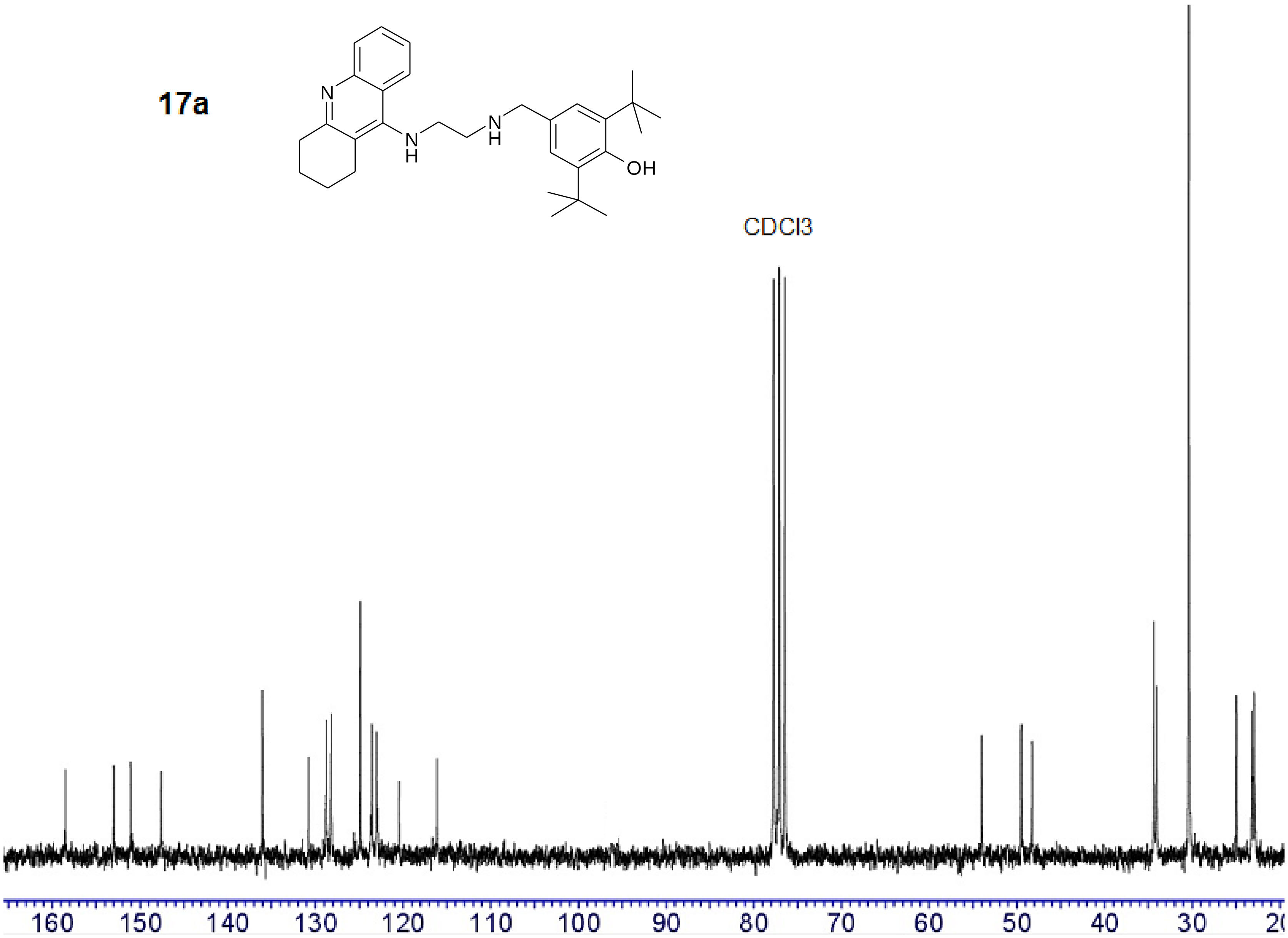

Figure S22. ^{13}C NMR spectrum of compound **17a**

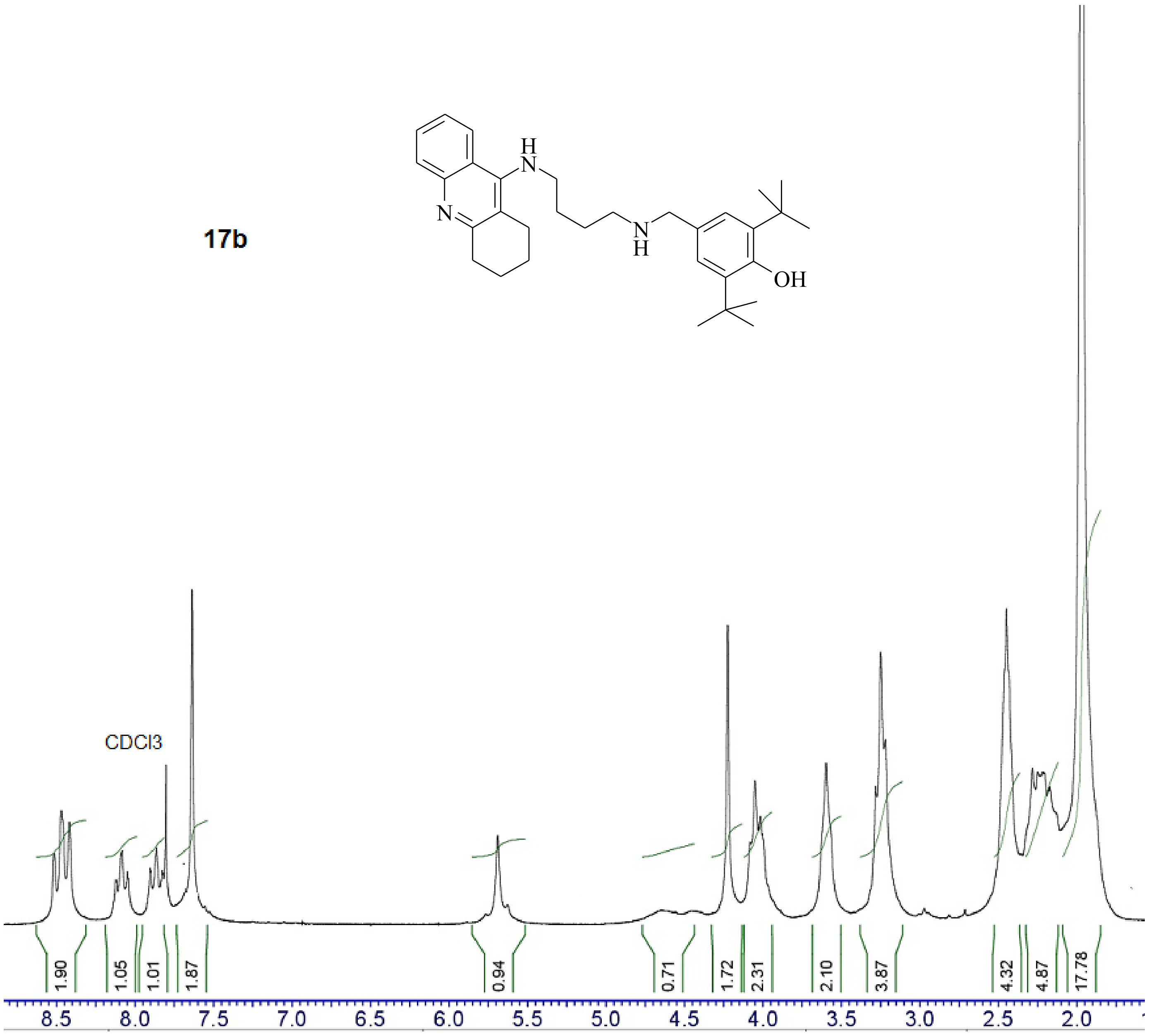

Figure S23. ^{1}H NMR spectrum of compound **17b**

17b

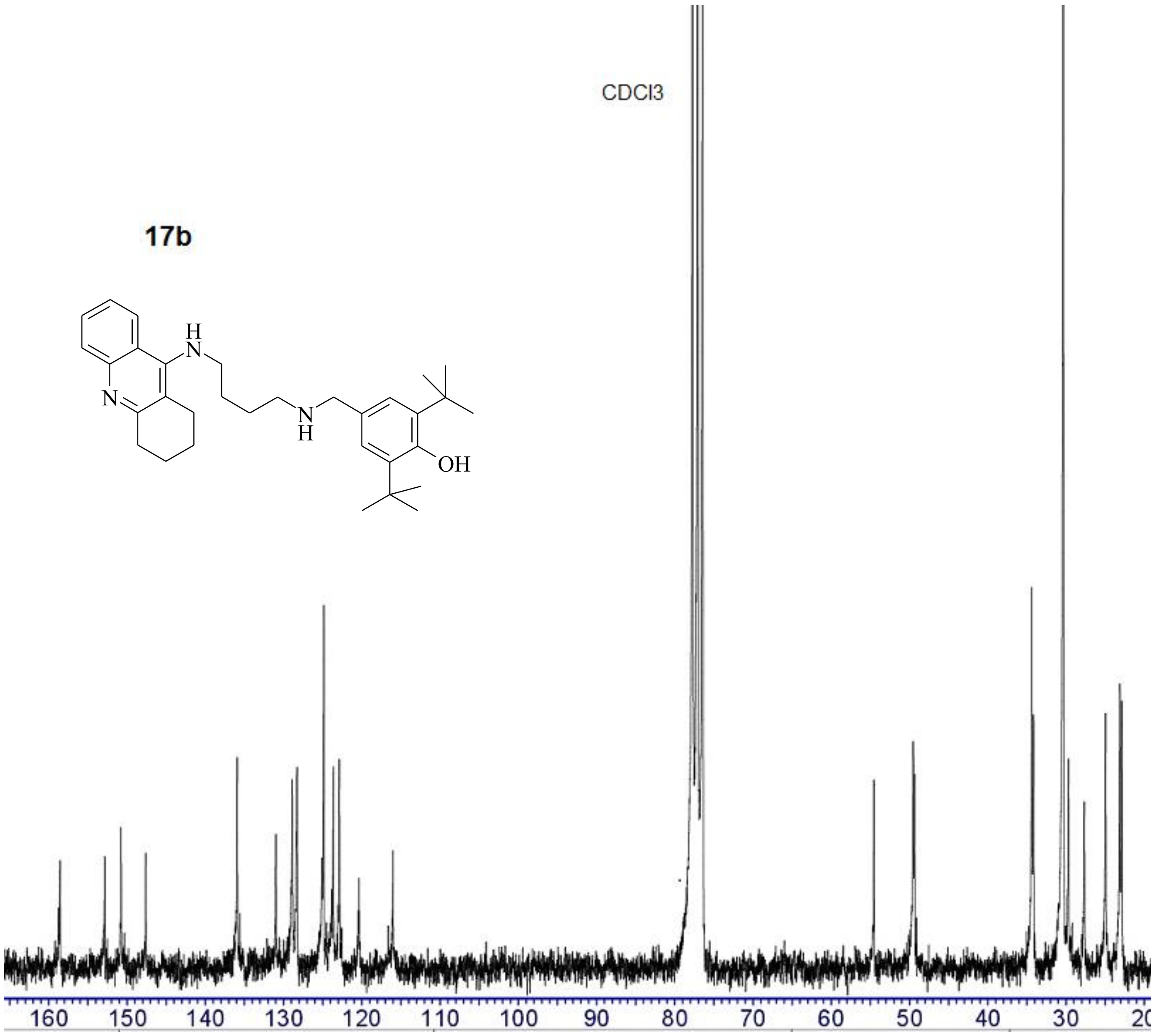

Figure S24. ^{13}C NMR spectrum of compound **17b**

17c

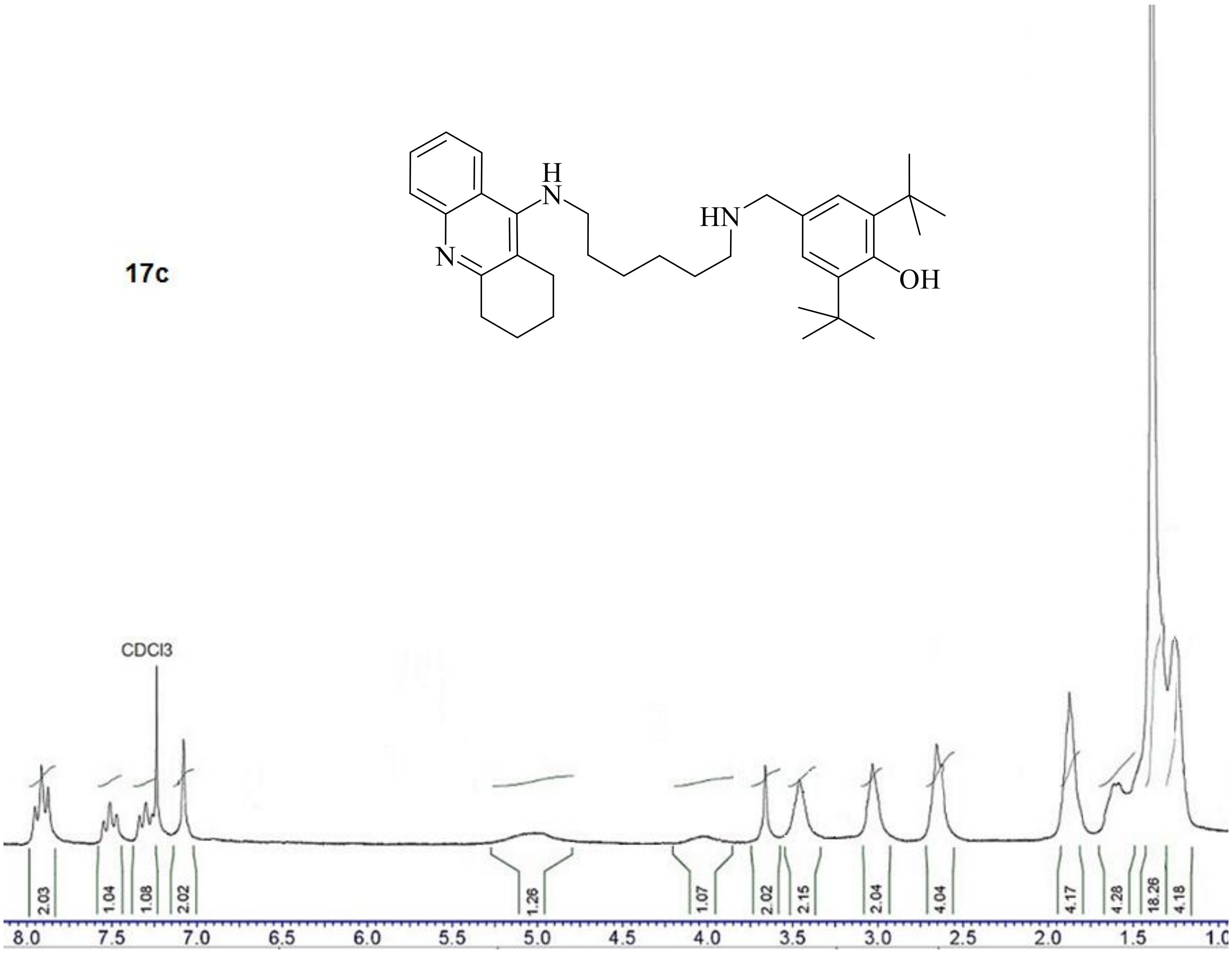

Figure S25. ^{1}H NMR spectrum of compound **17c**

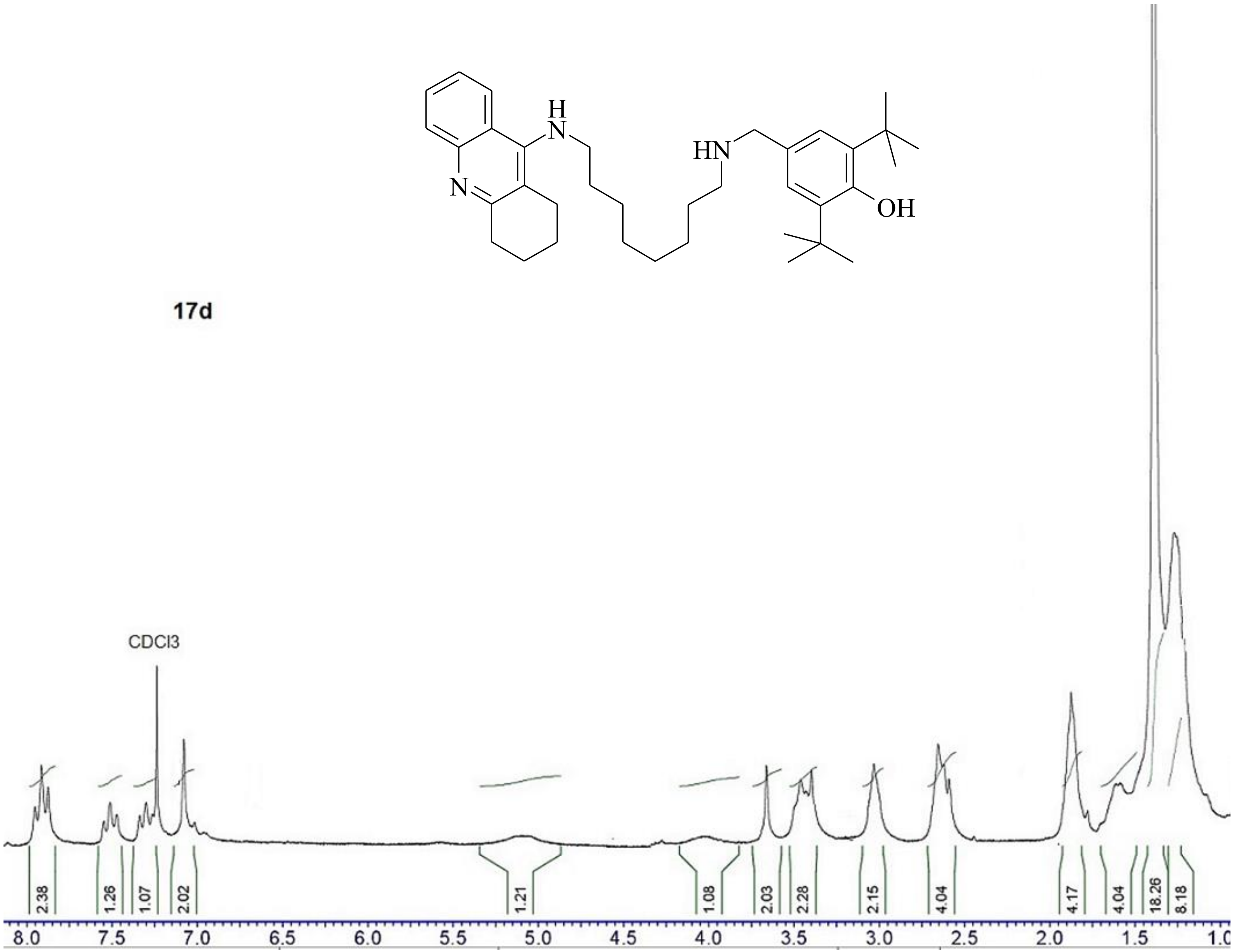

Figure S26. ^{1}H NMR spectrum of compound **17d**

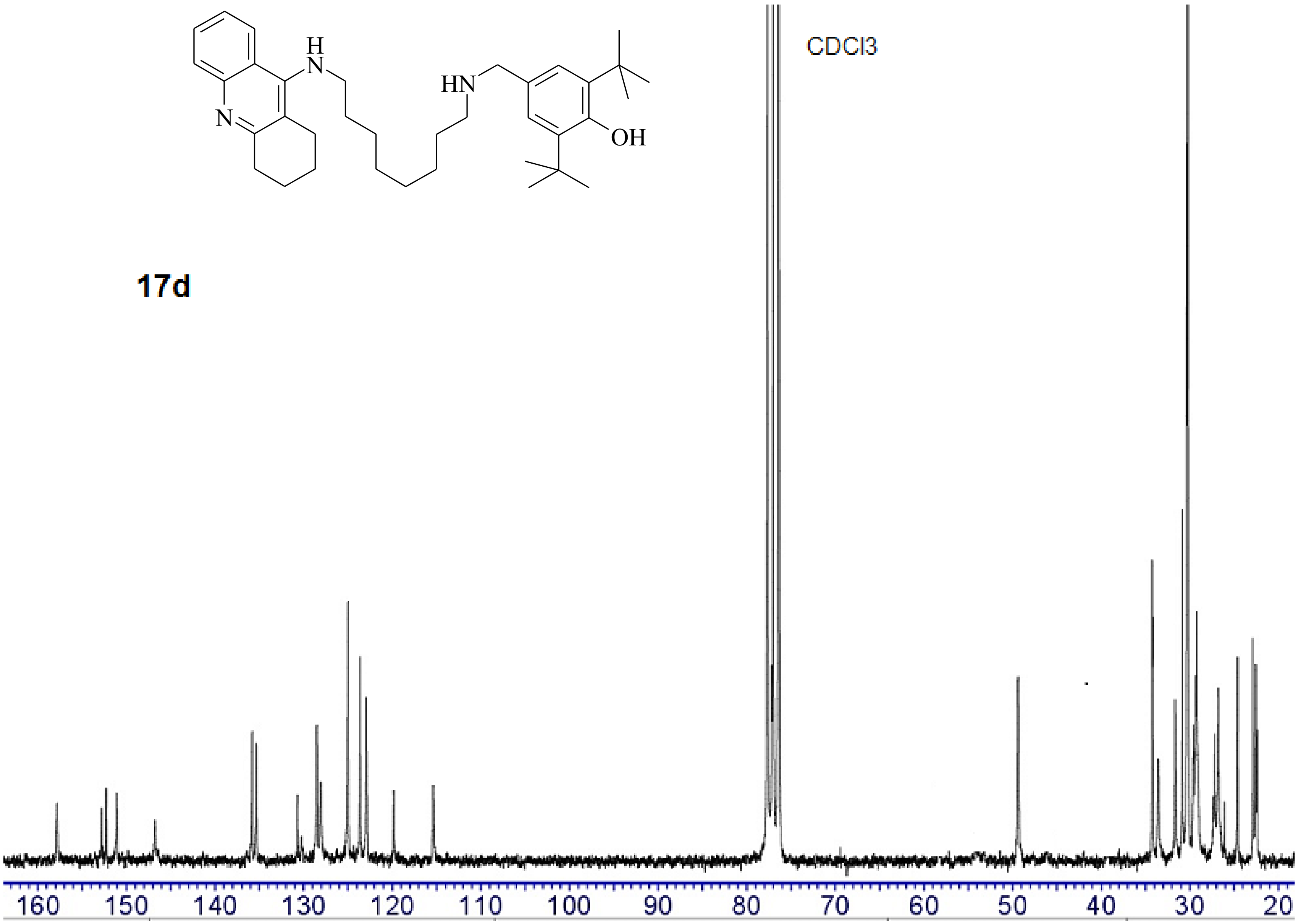

Figure S27. ^{13}C NMR spectrum of compound **17d**

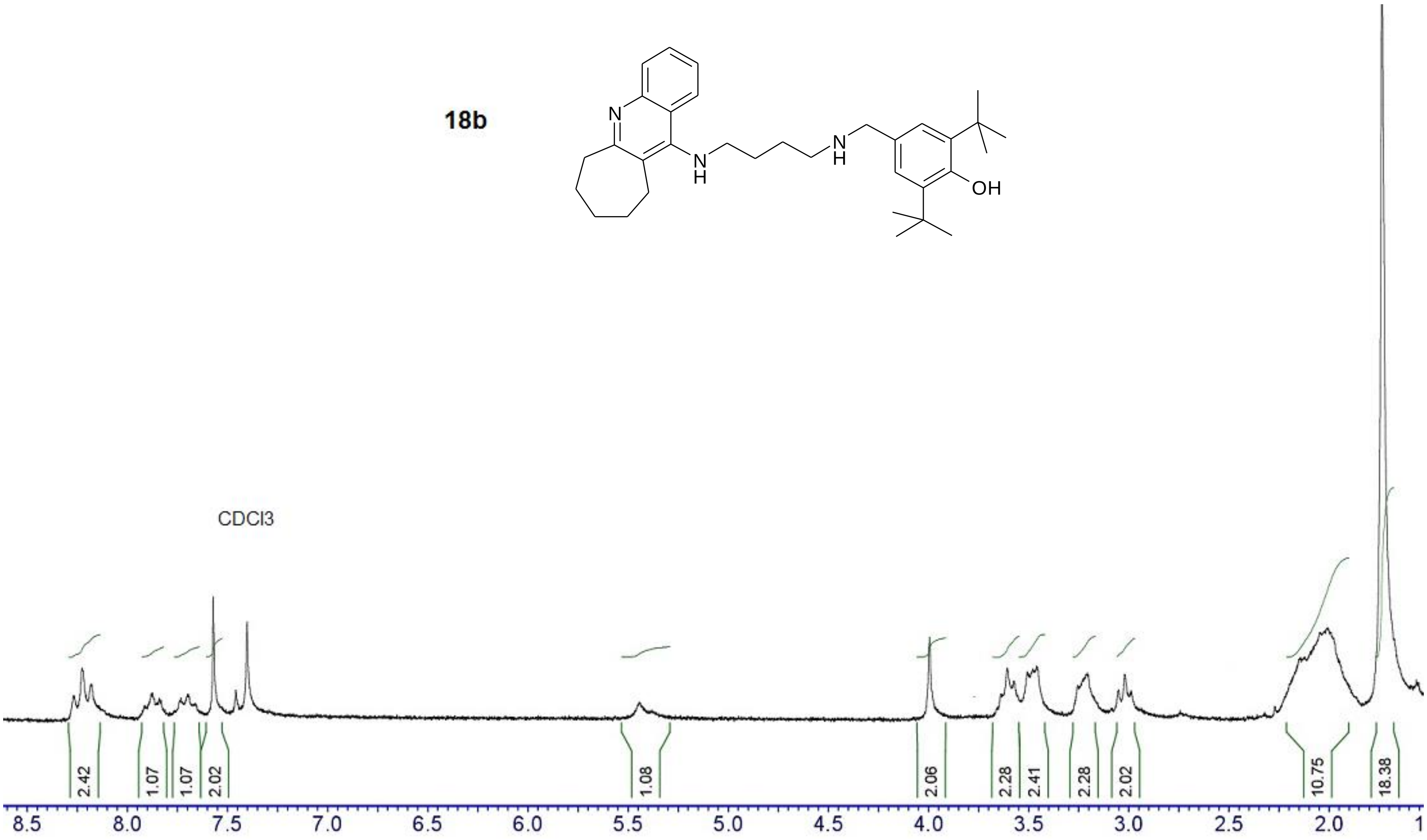

Figure S28. ^{1}H NMR spectrum of compound **18b**

18b

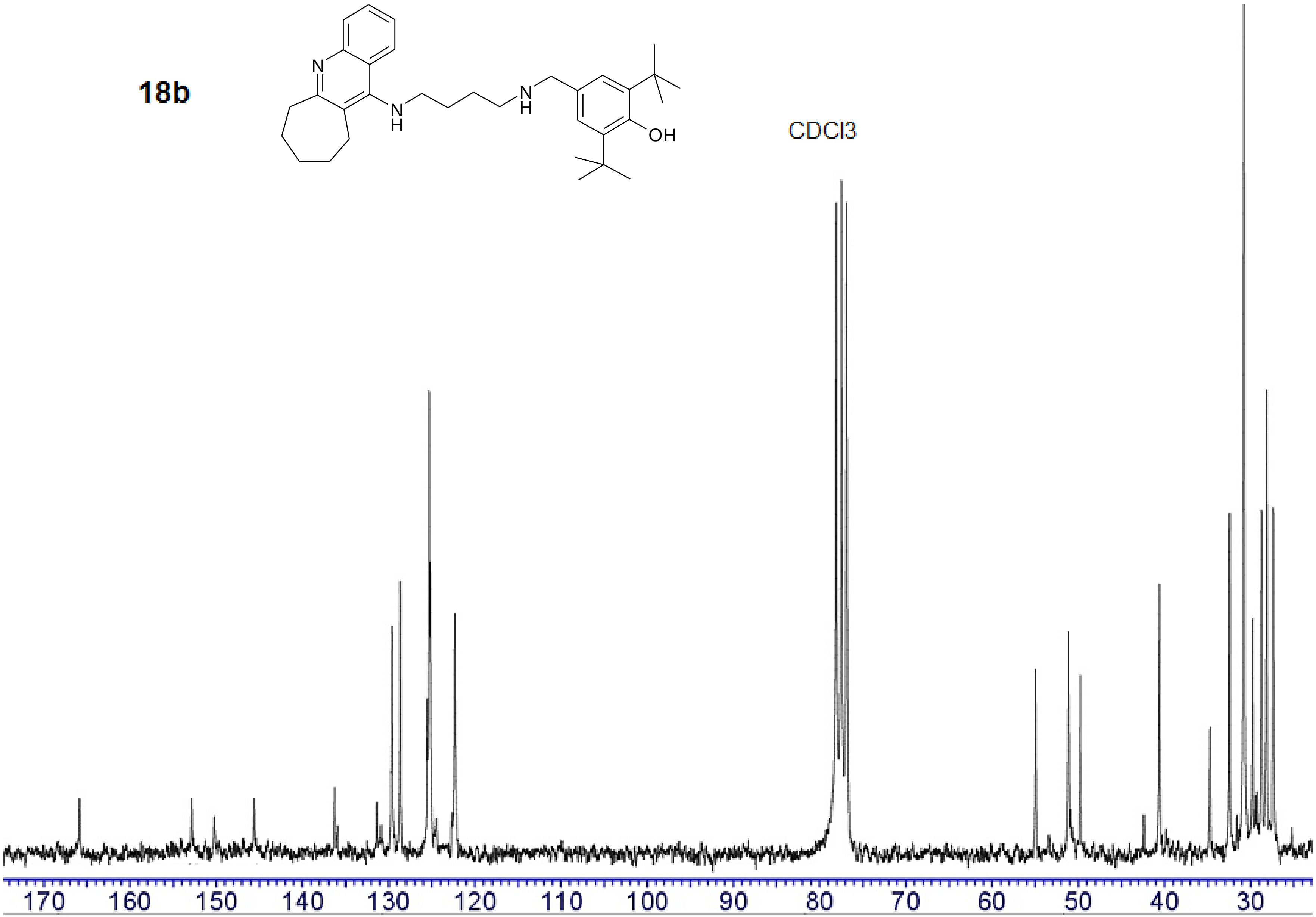

Figure S29. ^{13}C NMR spectrum of compound **18b**

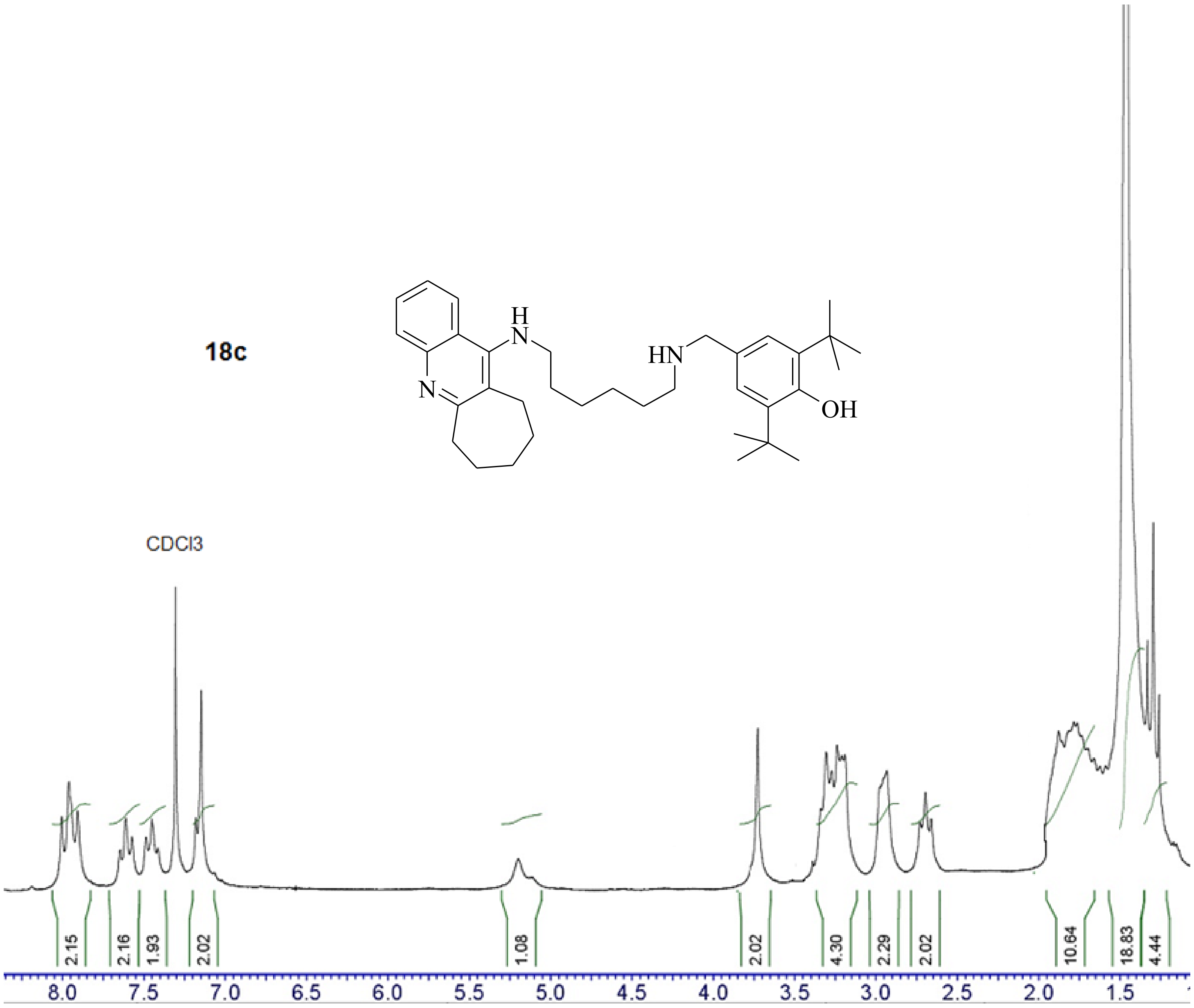

Figure S30. ^{1}H NMR spectrum of compound **18c**

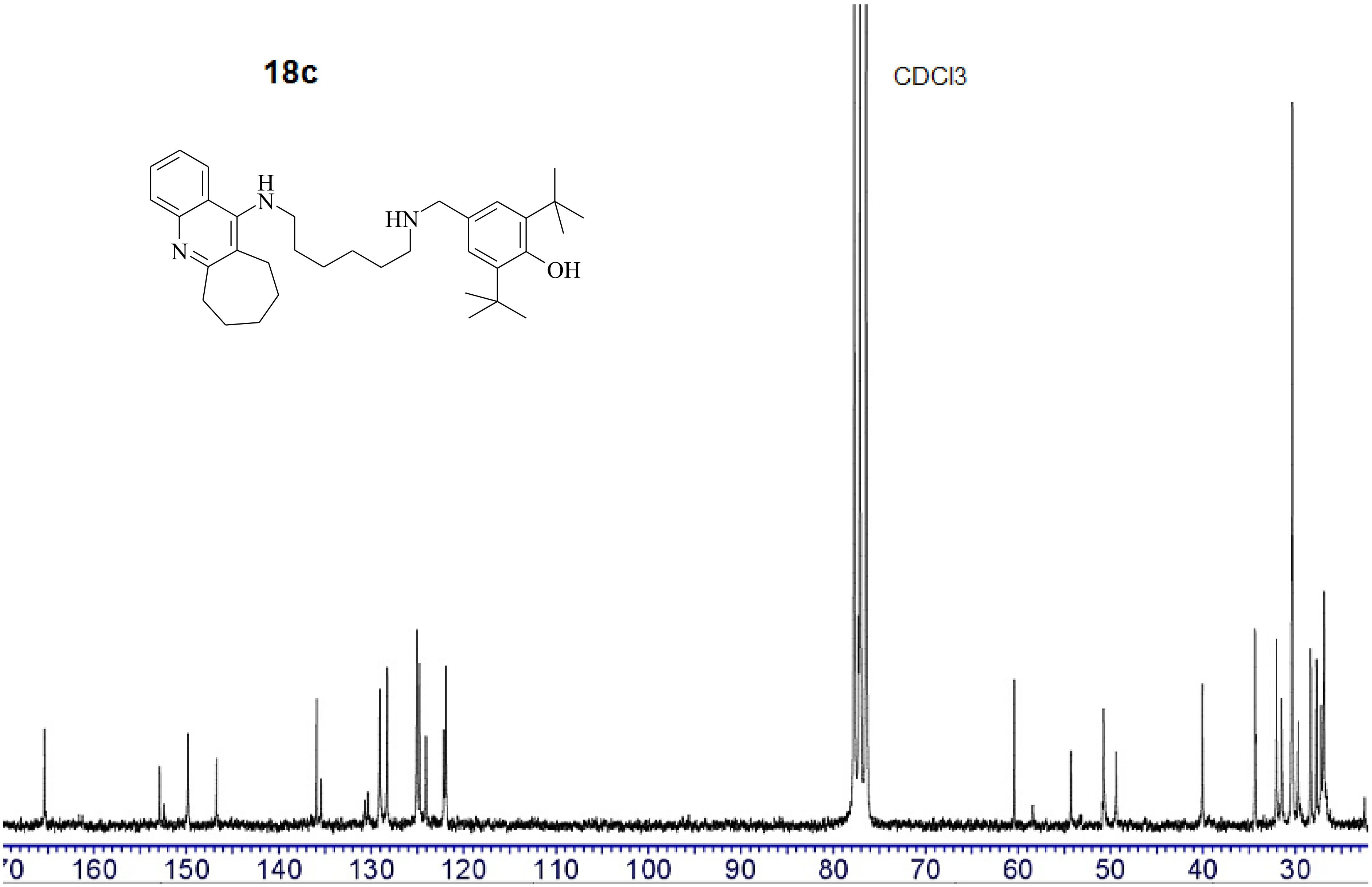

Figure S31. ^{13}C NMR spectrum of compound **18c**

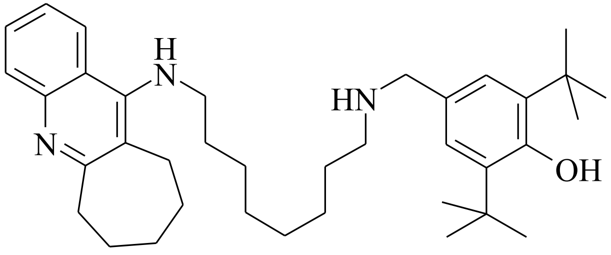

18d

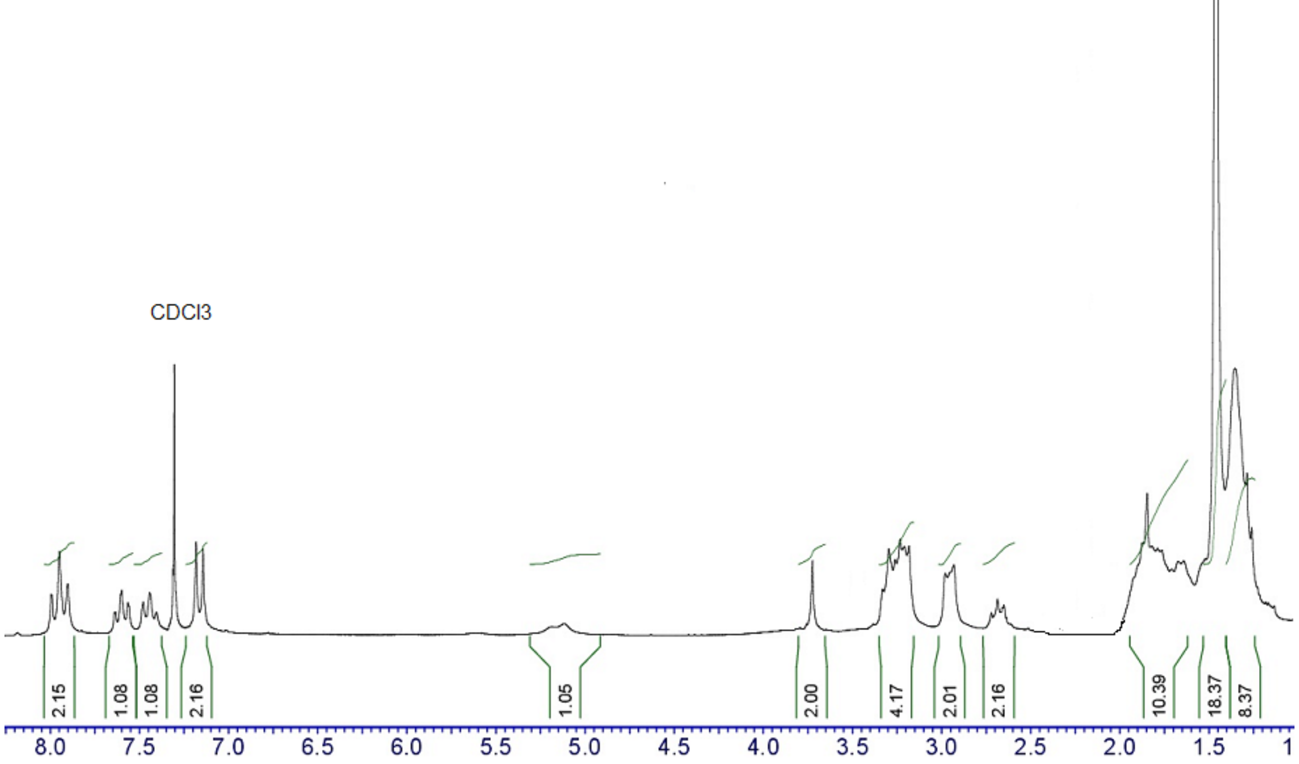

Figure S32. ^{1}H NMR spectrum of compound **18d**

18d

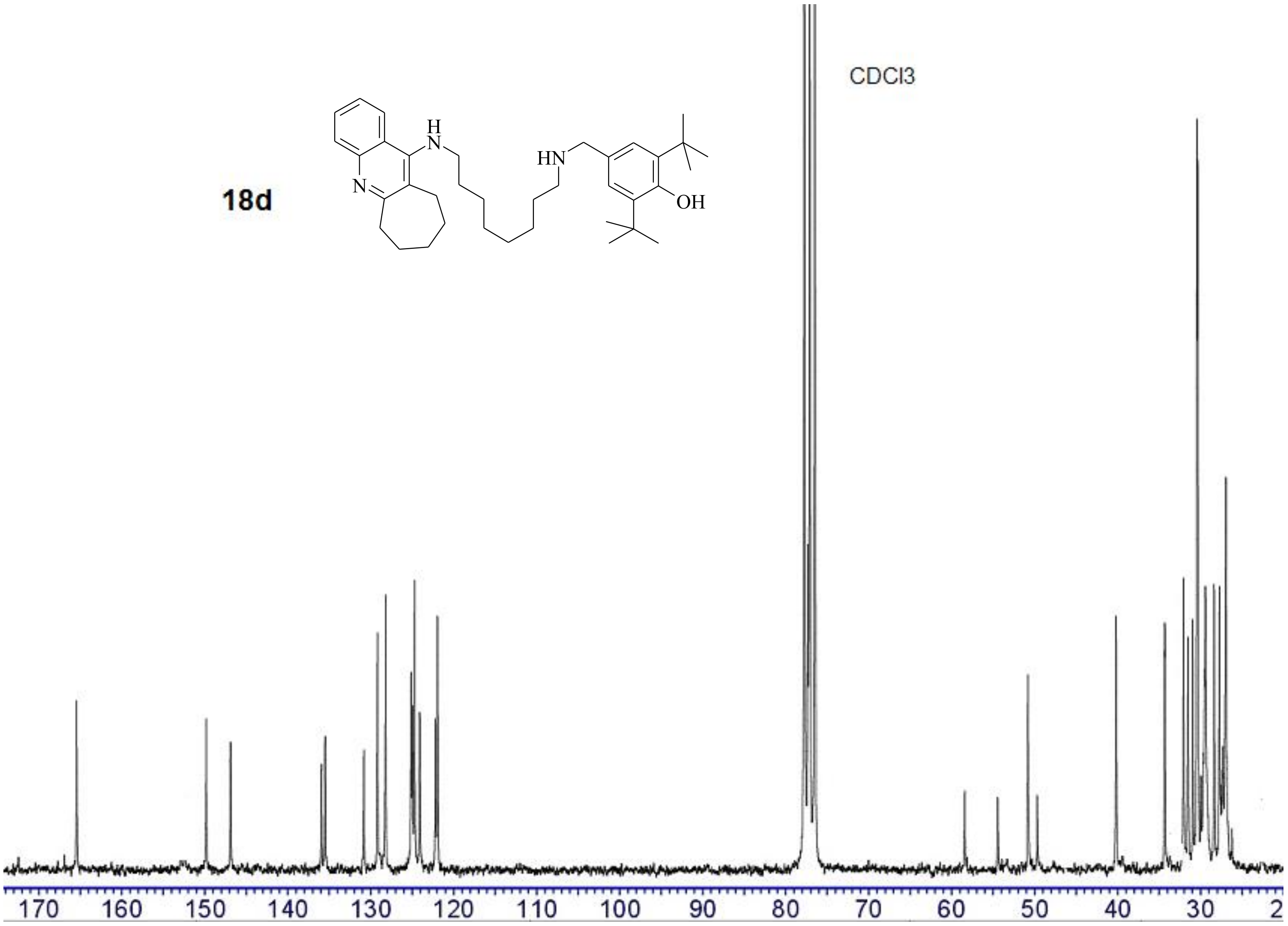

Figure S33. ^{13}C NMR spectrum of compound **18d**

Quantum-Chemical Calculations of AOA

Estimation of protonation state

To explain the AOA, it is necessary first of all to determine the protonation state of all compounds under consideration in each experiment. Table S1 presents p*K*a values of **14b**, **17b**, **BHT** as well as **tacrine** as a reference, according to Marvin prediction. The experimental pKa of tacrine is 9.95. Thus, Marvin slightly underestimates the p*K*a of tacrine. It can be said with certainty that in the TEAC and FRAP experiments both **14b** and **17b** are doubly protonated at the N atoms in the tacrine moiety and spacer. In chemiluminescence (CL) and inhibition of spontaneous lipid peroxidation (LP) experiments at pH 7.4, **17b** is also doubly protonated. As for **14b**, it is definitely protonated at N in the tacrine moiety, but its protonation state at N in the spacer is questionable. For **14b**, it is necessary to calculate the antioxidant characteristics for both doubly and singly protonated states and check which characteristics are in better agreement with experiment. Below, the state of protonation in the tacrine moiety and the spacer is denoted by the subscripts t and s, respectively.

Table S1. p*K*a values according to Marvin prediction

Structure	N in tacrine moiety	N in spacer	OH in BHT moiety
**14b (imine)**	8.9	7.5	10.5
**17b (amine)**	8.8	9.6	10.8
**Tacrine**	9.0	n/a	n/a
**BHT**	n/a	n/a	11.6

n/a is not available

We also calculated proton affinity (PA), which generally correlates with p*K*a. The results are presented in Table S2. The calculated PA correlates with Marvin's prediction that amines are protonated at N in the spacer much better than imines. The calculated PA values show that imines and amines first protonate in the tacrine moiety and then, if this occurs, protonate in the spacer.

Table S2. PA in water, kcal/mol.

Here: 'neu→t' means protonation of the neutral molecule at N in tacrine moiety; 'neu→s' means protonation of the neutral molecule at N in the spacer; 't→ts' means protonation of the protonated tacrine moiety at N in the spacer.

	neu→t	neu→s	t→ts
**14b (imine)**	35.1	32.3	31.4
**17b (amine)**	35.4	33.5	32.9
**Tacrine**	34.9	n/a	n/a

n/a is not available

To evaluate the antioxidant activity of compounds **14b**, **17b**, and **BHT**, the bond dissociation enthalpy (BDE), vertical ionization potential (IP), vertical proton affinity (EA_v) and proton dissociation enthalpy (PDE) were calculated as is commonly done [S1-S3]. The solvation enthalpies of the proton and electron in water and ethanol were taken from the literature [S4].

The calculated antioxidant characteristics in water and ethanol are given in Tables S3 and S4, respectively. The lowest BDE value is given for each compound, which is for OH bond in the BHT moiety. PDE values were also calculated for the OH bond in the BHT moiety.

Table S3. AOA characteristics in water, kcal/mol.

Protonation state	compound	BDE[1]	IP	EA	PDE[1]
neutral	**BHT**	75.1	105.3	-12.7	41.0
	**14b (imine)**	76.9	99.2	12.7	36.1
	**17b (amine)**	76.1	98.9	12.5	40.4
protonated, s	**$14b_s$ (imine)**	83.7	99.9	37.6	27.1
	**$17b_s$ (amine)**	79.2	100.1	13.4	37.4
protonated, t	**$14b_t$ (imine)**	77.1	108.4	26.7	35.9
	**$17b_t$ (amine)**	76.1	106.1	26.6	40.0
double-protonated, ts	**$14b_{ts}$ (imine)**	83.7	113.9	38.1	26.7
	**$17b_{ts}$ (amine)**	79.2	114.0	27.3	37.0

[1]For OH bond in BHT moiety

Table S4. AOA characteristics in ethanol, kcal/mol.

Protonation state	compound	BDE1	IP	EA	PDE1
neutral	BHT	74.0	112.6	-8.1	41.1
	14b (imine)	76.4	105.7	17.0	35.9
	17b (amine)	76.0	105.5	16.9	40.0
protonated, s	14bs (imine)	85.4	107.5	45.0	24.1
	17bs (amine)	78.8	107.7	18.7	35.3
protonated, t	14bt (imine)	76.5	116.3	33.8	34.9
	17bt (amine)	75.9	114.0	33.7	39.3
double-protonated, ts	14bts (imine)	83.6	124.6	46.3	23.0
	17bts (amine)	78.9	124.8	35.4	34.6

[1]For OH bond in BHT moiety

The following conclusions are drawn from **Tables S3** and **S4**:

- Antioxidant characteristics are highly dependent on the protonation state.
- The trends in water and ethanol are the same.
- Protonation in the tacrine moiety has little effect on BDE and increases IP.
- Protonation in the spacer has little effect on IP and increases BDE. Moreover, BDE of imines increases much more than the BDE of amines. The reason is a distortion of conjugation in the BHT moiety. As the imine N, in contrast to the amine N, is involved in conjugation in the BHT moiety, a change in the electronic structure at the N atom in imines affects the conjugation much more than in amines.
- Increasing the protonation degree from single protonation in the tacrine moiety to double protonation in both the tacrine moiety and the spacer impairs electron donation and increases electron acceptability. Moreover, the ability to accept an electron in imines increases much more than in amines.
- Protonation in the spacer significantly decreases PDE. Moreover, the PDE of imines decreases much more than the PDE of amines. The reason is the same as in the case of the BDE.

The results of the four different tests are summarized in Table 7 of the main text. CL and LP were performed under close conditions (pH 7.4) and with a similar set of free radicals in the mouse brain homogenate. However, the results of CL and LP are fundamentally different. In CL, imines show much higher AOA than amines. In LP, the AOA of amines is slightly better than that of imines. In addition, BHT

shows almost no AOA in CL and good AOA in LP. This suggests that LP and CL have different mechanisms of antioxidant actions.

Luminol chemiluminescence

Superoxide plays a key role in luminol chemiluminescence [S5]. It does not react directly with luminol, but reacts with the luminol radical ($L^{\bullet -}$) formed in the reactions of luminol with many radicals (e.g., $^{\bullet}OH$ and $CO_3^{\bullet -}$). The reaction between the luminol radical and superoxide results in the formation of 3-aminophthalate in the excited state. The luminescence of the latter is detected in the experiment [S6]. Thus, in CL test, the luminol luminescence can be reduced by quenching not only free radicals in the mouse brain homogenate, but superoxide as well. The fundamentally different results of the CL and LP tests suggest that it is superoxide radical quenching that is responsible for the decrease in luminol luminescence in CL.

We estimated the ability of the superoxide quenching for four compounds in water. They are **14b**$_{ts}$, **14b**$_{t}$, **17b**$_{ts}$, and **BHT**. We considered the double protonated amine predicted by Marvin at pH 7.4, and the singly and doubly protonated imine, as we do not know exactly its protonation state at pH 7.4.

For the compounds under consideration seven reactions of the superoxide reduction are possible [S7]:

$$O_2^{\bullet -} + AH \rightarrow O_2 + AH^{\bullet -} \quad (S1)$$

$$O_2^{\bullet -} + AH \rightarrow O_2^{-2} + AH^{\bullet +} \quad (S2)$$

$$2O_2^{\bullet -} + 2AN_tH^+N_sH^+OH \rightarrow H_2O_2 + O_2 + 2AN_tH^+N_sOH \quad (S3)$$

$$2O_2^{\bullet -} + AN_tH^+N_sH^+OH \rightarrow H_2O_2 + O_2 + AN_tN_sOH \quad (S4)$$

$$2O_2^{\bullet -} + 2AN_tH^+N_sOH \rightarrow H_2O_2 + O_2 + 2AN_tN_sOH \quad (S5)$$

$$2O_2^{\bullet -} + 2AOH \rightarrow H_2O_2 + O_2 + 2AO^- \quad (S6)$$

$$O_2^{\bullet -} + 2AOH \rightarrow H_2O_2 + AO^{\bullet} + AO^- \quad (S7)$$

In reactions (S1), (S2), (S6) and (S7) AH is the antioxidant molecule, $AH^{\bullet -}$ is the antioxidant anion radical, $AH^{\bullet +}$ is the antioxidant cation radical, $AO^{\bullet}$ is the antioxidant radical after H-atom abstraction, and AO^- is the deprotonated antioxidant. In reactions (S3) - (S5), $AN_tH^+N_sH^+OH$ is double protonated form of **14b** or **17b**, $AN_tH^+N_sOH$ is **14b** or **17b** protonated by N in the tacrine moiety, AN_tN_sOH is neutral **14b** or **17b**.

Electron transfer (ET) reactions (S1) and (S2) are available for all compounds. Reactions (S3) and (S4) are available for doubly protonated amines and imines. Reaction (S5) is available for imines protonated at the N atom in the tacrine moiety. Reactions (S6) and (S7) are available for all compounds, in imines and amines, and an H-atom or proton is abstracted in the **BHT** moiety.

Reactions (S1), (S2), (S6), (S7) refer to basic modes of action for the superoxide radical [S7,S8].

Reactions (S3)-(S6) are similar to the dismutation of the superoxide radical ($O_2^{\bullet -}$) into hydrogen peroxide (H_2O_2) and oxygen (O_2) in biological systems [S9]. In reactions (S3)-(S6), the proton source is deprotonation of antioxidant molecules. Reaction (S7) is similar to that proposed for flavonoids and other compounds where an H-atom can be abstracted [S10]and references therein.

For all reactions, the change in free energy, $\Delta G^{(i)}$, was calculated, where *i* indicates the reaction number (from S1 to S7). The change in free energy was calculated as the difference between the total free energy of the reactants on the right- and left-hand sides of Eqs. (S1) - (S7). The free energies of reactants were calculated for ground-state geometries in water at T = 298 K. The resultant $\Delta G^{(i)}$ values are presented in Table S5. An increase in free energy is shown in red, and a decrease in free energy is shown in green. The change in free energy of the most favorable reaction for each compound is shown in bold. For clarity, $\Delta G^{(i)}$ values are illustrated in Figure S34.

Table S5. Change in free energy, $\Delta G^{(i)}$, for reactions (S1)-(S7) in kcal/mol.

compound	$\Delta G^{(1)}$	$\Delta G^{(2)}$	$\Delta G^{(3)}$	$\Delta G^{(4)}$	$\Delta G^{(5)}$	$\Delta G^{(6)}$	$\Delta G^{(7)}$
**BHT**	73.0	71.2	n/a	n/a	n/a	3.0	5.0
**14b$_t$**	30.4	76.5	n/a	n/a	-3.5	-2.5	1.4
**14b$_{ts}$**	21.7	84.0	-7.9	-5.7	n/a	-12.0	-2.0
**17b$_{ts}$**	30.0	81.6	-5.3	-4.5	n/a	-0.4	5.4

n/a is not available

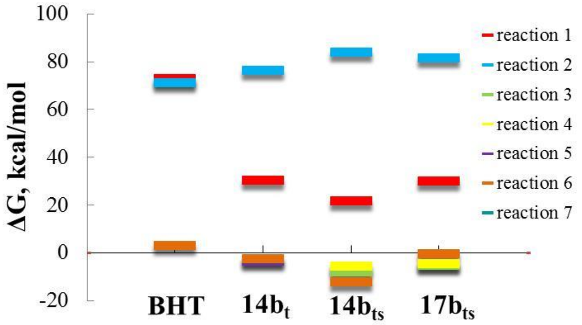

Figure S34. Change in free energy, ΔG as a result of reaction (S1) - (S7) for **BHT**, **14b$_t$**, **14b$_{ts}$**, and **17b$_{ts}$** compounds.

The following conclusions can be drawn from Table S5 and FigureS34:

- Both ET reactions (reactions (S1) and (S2)) for all the compounds under consideration proceed with a large increase in free energy, which means that the superoxide reduction in the CL test does not occur via the ET mechanism.
- For **BHT**, all reactions proceed with an increase in free energy, which means that **BHT** reduces the superoxide radical poorly.
- For imines and amines, proton-donating reactions (reactions (S3) - (S6)) proceed with a decrease in free energy. The mixed H-atom and proton-donating reaction (S7) slightly increases the free energy in **14b$_t$** and **17b$_{ts}$** and slightly decreases the free energy in **14b$_{ts}$**.
- The largest decrease in free energy is shown by doubly protonated imines, for which all proton-donating reactions proceed with a decrease in free energy.
- For doubly protonated imines, the most favorable reaction is (S6), where the antioxidant donates the proton from the BHT moiety.
- For doubly protonated amines, the most favorable reaction is (S3), where the antioxidant donates the proton from the spacer.
- Analysis of Table S5 and Figure S34 shows that to explain the much higher AOA of imines than amines in the CL test, it is necessary to assume that **both imines and amines are doubly protonated under experimental conditions (pH 7.4)**.

Thus, the decrease in the luminol luminescence in the CL test occurs due to the reduction of the superoxide radical. The reduction proceed through proton donation by the antioxidant, which is similar to the dismutation of the superoxide radical ($O_2^{\bullet-}$) into hydrogen peroxide (H_2O_2) and oxygen (O_2) in biological systems. In the CL test, the AOA of the compounds under consideration is determined by their PDE. The lower the PDE value, the higher the AOA. A difference in PDE values of 6 kcal/mole decreases the IC_{50} value by about six times.

Inhibition of spontaneous lipid peroxidation

The hydroxyl radical quenching by H-atom transition from the antioxidant to the radical (reaction (2) of the main text) proceeds through the following stages:

$$AOH + HO^{\bullet} \rightarrow [AOH - HO]^{\bullet} \rightarrow [AO - HOH]^{\bullet} \rightarrow AO^{\bullet} + H_2O \quad (S8)$$

The reaction pathway is illustrated in Figure S35.

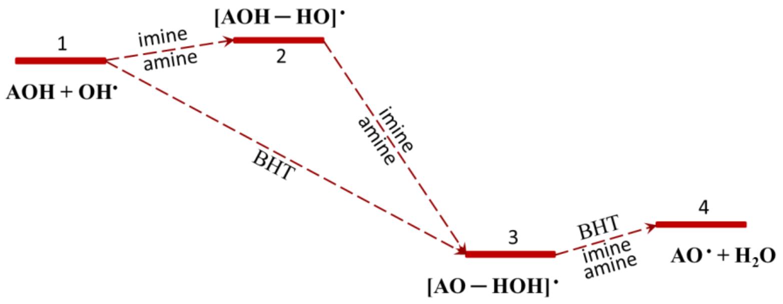

Figure S35. Reaction pathway for quenching the $HO^{\bullet}$ radical via the H-atom transfer from the antioxidant molecule.

The analysis of AOA of amines and imines in the CL test shows that both are doubly protonated at pH 7.4. As the pH value in the thiobarbituric acid reactive substances (TBARS) assay is the same as in the CL method, we assume that amines and imines are also doubly protonated in the CL test.

Table S6. Inhibition of spontaneous lipid peroxidation in mice brain homogenate (TBARS Assay), BDE and reactant energies at all stages of reaction (S8).

	IC_{50}, μM	BDE, kcal/mol	1: $AOH + HO^{\bullet}$, kcal/mol	2: $[AOH - HO]^{\bullet}$, kcal/mol	3: $[AO - HOH]^{\bullet}$, kcal/mol	4: $AO^{\bullet} + H_2O$, kcal/mol
**BHT**	7	75.1	0	-	-46.7	-44.3
**17b$_{ts}$**	20	79.2	0	0.5	-42.5	-40.1
**14b$_{ts}$**	25	83.7	0	0.8	-37.4	-35.4

The sum of the energies of reactants at four stages of reaction (S8) are given in Table S6. It can be seen that the inhibition of spontaneous lipid peroxidation in mouse brain homogenate correlates with the ability of the antioxidant to donate an H atom.

In Table S6, the total energy of the initial reagents (stage 1) is assumed to be zero. For **14b$_{ts}$** and **17b$_{ts}$,** the reaction proceeds through formation of two intermediate complexes $[AOH - HO]^{\bullet}$ (stage 2) and $[AO - HOH]^{\bullet}$ (stage 3). The $[AOH - HO]^{\bullet}$ energy only slightly exceeds the summed energies of the initial reagents. For **BHT**, the $[AOH - HO]^{\bullet}$ complex does not exist at all. This means that at room temperature the formation of the $[AO - HOH]^{\bullet}$ complex proceeds without a barrier. The energy of the $[AO - HOH]^{\bullet}$ complex is much lower than the energy of the initial reactants. The energy gain in the $[AO - HOH]^{\bullet}$ complex formation also correlates with the AOA of the considered compounds.

All the above considerations confirm that the inhibition of spontaneous lipid peroxidation in mouse brain homogenate proceeds mainly through H-atom transfer from antioxidant molecules to radicals. The slightly higher AOA of amines than imines is due to easier H-atom abstraction in the BHT moiety, which is reflected in a lower BDE of amines in doubly protonated form (see Table S3).

ABTS and FRAP tests

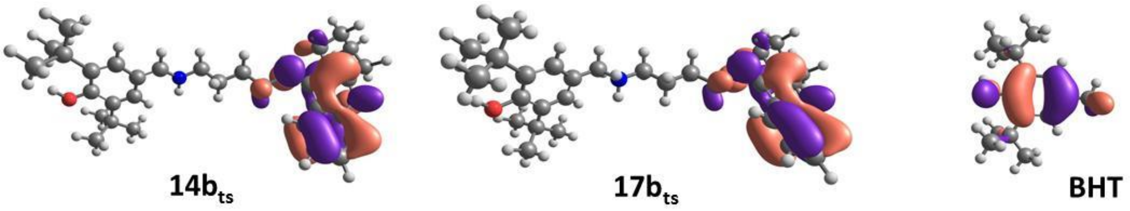

Figure S36. Visualization of frontier molecular orbitals: antioxidants HOMO.

Figure S36 presents the HOMO of antioxidants **14b**$_{ts}$, **17b**$_{ts}$, and BHT. The HOMO of **14b**$_{ts}$ and **17b**$_{ts}$, are located on the tacrine moiety. This means that the complexes formed during the ET are different for conjugates **14b**$_{ts}$**, 17b**$_{ts}$ and for BHT. That is why there is no direct correlation between the IP values of **14b**$_{ts}$, **17b**$_{ts}$ and BHT and their AOA in the ABTS and FRAP tests.

References

S1. Baj, A.; Cedrowski, J.; Olchowik-Grabarek, E.; Ratkiewicz, A.; Witkowski, S. Synthesis, DFT Calculations, and In Vitro Antioxidant Study on Novel Carba-Analogs of Vitamin E. *Antioxidants (Basel)* **2019**, *8*, doi:10.3390/antiox8120589.

S2. Xue, Y.; Zheng, Y.; An, L.; Dou, Y.; Liu, Y. Density functional theory study of the structure-antioxidant activity of polyphenolic deoxybenzoins. *Food Chem.* **2014**, *151*, 198-206, doi:10.1016/j.foodchem.2013.11.064.

S3. Hossen, J.; Pal, T.K.; Hasan, T. Theoretical investigations on the antioxidant potential of 2,4,5-trihydroxybutyrophenone in different solvents: A DFT approach. *Results Chem.* **2022**, *4*, doi:10.1016/j.rechem.2022.100515.

S4. Marković, Z.; Tošović, J.; Milenković, D.; Marković, S. Revisiting the solvation enthalpies and free energies of the proton and electron in various solvents. *Comput. Theor. Chem.* **2016**, *1077*, 11-17, doi:10.1016/j.comptc.2015.09.007.

S5. Bedouhène, S.; Moulti-Mati, F.; Hurtado-Nedelec, M.; My-Chan Dang, P.; El-Benna, J. Luminol-amplified chemiluminescence detects mainly superoxide anion produced by human neutrophils. *Am. J. Blood Res.* **2017**, *7*, 41-48.

S6. Villaverde, A.; Netherton, J.; Baker, M.A. From Past to Present: The Link Between Reactive Oxygen Species in Sperm and Male Infertility. *Antioxidants (Basel)* **2019**, *8*, doi:10.3390/antiox8120616.

S7. Raghuvanshi, R.S. Superoxide Anion Radical, A Multipotent Reagent: A Review *Nat. Volatiles & Essent. Oils* **2020**, *7* 75-82.

S8. Galano, A.; Vargas, R.; Martinez, A. Carotenoids can act as antioxidants by oxidizing the superoxide radical anion. *Phys. Chem. Chem. Phys.* **2010**, *12*, 193-200, doi:10.1039/b917636e.

S9. Zhao, H.; Zhang, R.; Yan, X.; Fan, K. Superoxide dismutase nanozymes: an emerging star for anti-oxidation. *J. Mater. Chem. B* **2021**, *9*, 6939-6957, doi:10.1039/d1tb00720c.

S10. Ahmed, S.; Shakeel, F. Antioxidant activity coefficient, mechanism, and kinetics of different derivatives of flavones and flavanones towards superoxide radical. *Czech J. Food Sci.* **2012**, *30*, 153-163, doi:10.17221/447/2010-cjfs.
